# Evidence for selection at HIV host susceptibility genes in a West Central African human population

**DOI:** 10.1186/1471-2148-12-237

**Published:** 2012-12-06

**Authors:** Kai Zhao, Yasuko Ishida, Taras K Oleksyk, Cheryl A Winkler, Alfred L Roca

**Affiliations:** 1 Department of Animal Sciences, University of Illinois at Urbana-Champaign, Urbana, Il, 61801, USA; 2 Department of Biology, University of Puerto Rico at Mayaguez, Mayaguez, Puerto Rico , 00681; 3 Basic Research Laboratory, Center for Cancer Research, NCI, SAIC-Frederick, Frederick National Laboratory for Cancer Research, Frederick, , MD, 21702, USA; 4 Institute for Genomic Biology, University of Illinois at Urbana-Champaign, Urbana, , IL, 61801, USA

**Keywords:** HIV dependency factors, Single nucleotide polymorphisms, Biaka pygmies, Mbuti pygmies

## Abstract

**Background:**

HIV-1 derives from multiple independent transfers of simian immunodeficiency virus (SIV) strains from chimpanzees to human populations. We hypothesized that human populations in west central Africa may have been exposed to SIV prior to the pandemic, and that previous outbreaks may have selected for genetic resistance to immunodeficiency viruses. To test this hypothesis, we examined the genomes of Biaka Western Pygmies, who historically resided in communities within the geographic range of the central African chimpanzee subspecies (*Pan troglodytes troglodytes*) that carries strains of SIV ancestral to HIV-1.

**Results:**

SNP genotypes of the Biaka were compared to those of African human populations who historically resided outside the range of *P. t. troglodytes*, including the Mbuti Eastern Pygmies. Genomic regions showing signatures of selection were compared to the genomic locations of genes reported to be associated with HIV infection or pathogenesis. In the Biaka, a strong signal of selection was detected at *CUL5*, which codes for a component of the vif-mediated APOBEC3 degradation pathway. A *CUL5* allele protective against AIDS progression was fixed in the Biaka. A signal of selection was detected at *TRIM5,* which codes for an HIV post-entry restriction factor. A protective mis-sense mutation in *TRIM5* had the highest frequency in Biaka compared to other African populations, as did a protective allele for *APOBEC3G*, which codes for an anti-HIV-1 restriction factor. Alleles protective against HIV-1 for *APOBEC3H*, *CXCR6* and *HLA-C* were at higher frequencies in the Biaka than in the Mbuti. Biaka genomes showed a strong signal of selection at *TSG101,* an inhibitor of HIV-1 viral budding.

**Conclusions:**

We found protective alleles or evidence for selection in the Biaka at a number of genes associated with HIV-1 infection or progression. Pygmies have also been reported to carry genotypes protective against HIV-1 for the genes *CCR5* and *CCL3L1.* Our hypothesis that HIV-1 may have shaped the genomes of some human populations in West Central Africa appears to merit further investigation.

## Background

Variation in human genes is known to affect susceptibility to HIV-1 and disease progression following infection [1,2]. Hypothesis-based candidate gene studies have been conducted on natural history HIV cohorts established in the 1980s consisting of HIV-infected individuals or individuals at risk of HIV exposure by their inclusion in an HIV risk group [1]. This strategy has been highly productive and identified a number of gene variants associated with rate of HIV progression or resistance to infection: the *CCR5*-Δ32 mutation was shown to block HIV acquisition, and HLA class I genes were shown to be strongly associated with HIV progression and control of viral replication [3-6]. Common variants in the genes encoding ligands for the major HIV co-receptors, immune modifiers (HLA and cytokines) and post-entry restriction factors have been associated with a positive or negative effect on HIV pathogenesis [1,2]. More recently, genome wide association studies (GWAS) have been used to identify variants associated with infection, control of viral replication, and elite controller status [1,7-9].

In addition to genetic association studies, human host genes potentially required for HIV-1 infection have been identified using small interfering (si) RNA knockdown screens conducted on cell lines infected with HIV-1. Several siRNA studies have been independently conducted, each of which involved the knock-down of almost every human gene [10-12]. Each of the studies found over 200 human genes that were candidates for involvement in HIV-1 infection, designated HIV dependency factors (HDFs). However, there was little overlap in genes found across the studies [13], with only three human genes identified by all three knock-down studies, and 40 other genes detected by at least two of the studies [10-12].

HIV-1 derives from simian immunodeficiency viruses (SIV) infecting the common chimpanzee, *Pan troglodytes*[14]. HIV-1 sequences are polyphyletic in humans: each of the four HIV-1 groups present in humans (M, N, O, P) is closer in sequence to strains of SIV present in the central African subspecies (*P. t. troglodytes*) of the common chimpanzee than they are to HIV-1 sequences belonging to other human HIV-1 clades (Additional file 1: Figure S1) [14]. This suggests that HIV-1 originated in four or more independent cross-species transmissions from the *P. t. troglodytes* subspecies to humans [14]. The natural range of the central African chimpanzee is the Congolian forest block of Central Africa, west of the Congo River (Additional file 1: Figure S1), suggesting that each of the HIV-1 groups may have first infected humans living in this region, subsequently giving rise to the world-wide pandemic [14].

Archival medical samples collected in Leopoldville (now Kinshasa) during 1959 and 1960 are the earliest documented evidence of HIV-1 infections in humans [15]. The diversity of HIV-1 present in these and in subsequently collected samples has permitted the date of cross-species transmission for HIV-1 clade M viruses to be estimated as having occurred between 1884 and 1924 [15], with the other major clades originating within similar time frames [16]. By contrast the coalescence date for SIV strains in chimpanzees may be older than 20,000 years [15]. Since SIV has recently crossed the species barrier from chimpanzees to humans multiple times [14], we considered whether a virus known to have repeatedly entered human populations would only begin to do so in the past century or two. We hypothesized that the virus may also have repeatedly crossed the species barrier into local human populations before the current pandemic began. Simulation studies have suggested that SIV would be unlikely to have generated persistent outbreaks in humans in Central Africa before the appearance of large cities during the colonial era [17]. Additionally, it is possible that outbreaks prior to the current pandemic would have been extinguished due to the quick susceptibility of immunodeficient individuals to formerly pervasive infectious diseases (e.g., smallpox).

If immunodeficiency viruses had repeatedly affected human populations locally before the current pandemic, this may have generated selection pressure for resistance, which could be reflected in genomic signatures in the chromosomes of the living descendants of the affected populations. In considering this hypothesis, we found that a similar hypothesis had been independently formulated previously [18], but to our knowledge had never been tested. A number of difficulties would be encountered that make it difficult to test our hypothesis. First, any of the methods available to identify regions of the genome under selection would be likely to generate some false positive signals, and there would be uncertainty in the determination of regions of the genome under selection since some signals may result from other demographic factors or from drift. Methods to detect selection provide insight into putative regions under selection as an exploratory test, but would not be completely definitive [19,20]. Second, there is a degree of uncertainty regarding the identification of genes as human genes associated with HIV-1 (HGAHs) [1] or as HDFs. In particular (as noted above) the genes identified as HDFs show little overlap across different studies [1,10-12]. Third, many of the regions of the genome that show signatures of selection may contain multiple genes, and any of these could be responsible for a signal of selection. Fourth, selective pressure on host genes that interact with retroviruses would not necessarily be due to HIV-1, but could have been driven by other pathogens, such as other retroviruses known to affect humans or other primates within the African tropical forest [21-27].

Despite these caveats, we sought to test the hypothesis that previous outbreaks of immunodeficiency viruses may have shaped the genomes of some modern African populations. We found that the diverse populations intensively genotyped (~650,000 SNPs) [28] as part of the human genome diversity panel (HGDP) included the Biaka Western Pygmies of the Central African Republic. The Biaka have historically resided in communities within the forest range of *P. t. troglodytes*[29]. The Biaka and other pygmy groups diverged from their Bantu neighbors approximately 60–70,000 years ago [29,30]. Archeological evidence has suggested that the Western Pygmies have been in the Congo River basin for at least 18,000 years [31,32]. It is also likely that the Biaka or their ancestors were present in the Western Congolian rainforest since at least 2800 years, the time at which current Western pygmy populations are estimated to have separated genetically [33], concurrent with the Neolithic expansion of nonpygmy agriculturalists [34].

We compared Biaka genomes to those of HGDP African populations who live outside the range of *P. t. troglodytes*, including the Mbuti, an Eastern Pygmy population in the Democratic Republic of Congo. Eastern and Western pygmy groups diverged genetically ca. 20,000 years ago [29,30,35]. The chimpanzee subspecies *P.t. schweinfurthii* found in the forests inhabited by the Mbuti carries strains of SIV that fall outside the clades that gave rise to strains of HIV (Additional file 1: Figure S1) [14]. The other HGDP African populations live outside the geographic range of chimpanzee populations that carry SIV.

We examined SNP data for signatures of selection in the genomes of the Biaka around host genes shown to be associated with HIV disease or host genes that appear to interact with HIV in studies using cell lines (HDFs). We found that the genomic region surrounding the gene *CUL5*, encoding cullin 5, one of the strongest risk predictors of AIDS progression yet identified by candidate gene analysis [36], displayed a strong signature of recent selection in the Biaka. We also found signatures of selection at other HIV-associated genes in the Biaka.

## Results

**Figure 1:**
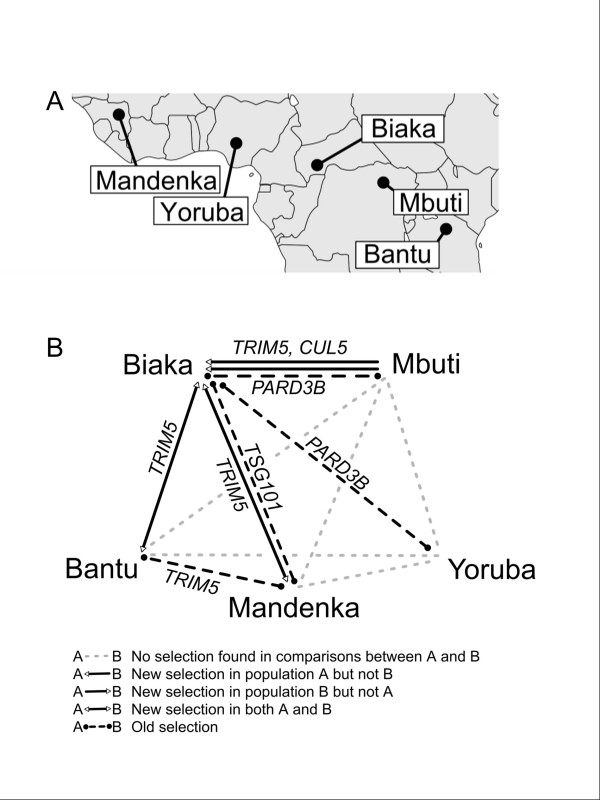
**Human populations examined for evidence of selection, and host genes associated with HIV-1 found to be under potential selection.** (**A**) Map showing locations of five human populations in sub-Saharan Africa that are part of the Human Genome Diversity Panel [37] and for which pairwise population genomic comparisons were conducted to identify regions potentially under selection. The Biaka are a Western Pygmy group in the Central African Republic that historically resided in a region where the strains of SIV that gave rise to HIV-1 are endemic in non-human primates. The Mbuti are an Eastern Pygmy group in the Democratic Republic of Congo. The Mbuti historically have resided where SIV strains that gave rise to HIV-1 are not present, as have the other non-Pygmy African populations listed. (**B**) Host genes associated with HIV-1 (HGAHs) under potential selection were revealed by genomic comparisons of each pair of African populations. The genomic locations of HGAHs were compared to regions of the genome identified using SNPs as potentially under selection [19] (regions of low heterozygosity within and/or high variance of *F*_*ST*_ between populations). HGAHs potentially under selection are indicated by the different lines or arrows connecting each pair of populations, with the type of selection indicated by the key. “New” selection in one or both populations indicates selection after the divergence of the two populations. “Old” selection is selection that occurred prior to the divergence of the two populations. There was evidence for selection in the Biaka for *CUL5*, *PARD3B, TRIM5*, and *TSG101*.

We looked for evidence of selection by comparing public SNP datasets between Biaka Western Pygmies and Mbuti Eastern Pygmies. We also ran selection scans using three other African populations (Figure 1A), running genomic comparisons between each pair of African populations. To look for genomic signatures of selection, we applied a method (previously developed for datasets unrelated to the current study) that relied on multi-locus heterozygosity within and *F*_*ST*_ variance between populations (Additional file 1: Figure S2) [19]. The proportion of the genome found to display signatures of selection for each pairwise comparison of populations is listed in Additional file 1: Table S1. Due in part to the way that selection tests were conducted, proportions of the genome identified as being under potential selection were similar across pairwise comparisons of different populations, ranging from 1.6% to 2.6% for autosomes. The comparison between Biaka and Mbuti Pygmy groups produced the lowest estimate for proportion of the genome showing signatures of selection, a total of 1.6% (Additional file 1: Table S1), perhaps reflecting the genetic affinity of the two Pygmy groups [29]. In this comparison, new selection in Biaka totaled 0.33% of the autosomes, new selection in Mbuti 0.40%, new selection in both populations 0.22%, and old selection 0.63% (Additional file 1: Table S1).

We examined genomic regions that demonstrated signatures of selection for the presence of host genes associated with HIV-1 (HGAHs), in which polymorphisms are known to affect HIV infection or outcome [1]. These genes had been found using candidate-gene or GWAS studies. For GWAS studies, only those with genome wide significance of *p* < 5 × 10^-8^ were further considered, in order to minimize the number of false positives, as suggested by [38]. There were 26 HGAH loci, although some loci included tightly linked gene clusters, so the total number of HGAHs was 45 clustered at the 26 loci, as listed and described in Additional file 1: Table S2. Across the five sub-Saharan African populations examined (Bantu, Biaka, Mandenka, Mbuti, Yoruba), only five of the ten pairwise comparisons detected any region with signatures of selection overlapping a HGAH (Figure 1B). These involved four distinct HGAHs (*CUL5*, *PARD3B, TRIM5*, and *TSG101*) that were detected as under putative selection a total of eight times across pairwise comparisons (Figure 1B). Remarkably, seven of the eight times in which signatures of selection overlapped with the genomic position of one of these genes involved evidence for old or new selection occurring in the Biaka population (Figure 1B).

We examined the degree to which the number of genes with signatures of selection detected among the HGAH listing was unusual relative to genes drawn at random, running a permutation test in which 26 genes at different loci were drawn at random and examined using the same test of selection in ten pairwise comparisons of the 5 African populations. We found that the probability that randomly drawn genes would overlap 7 or more signals of selection in a single population (the same number detected in Figure 1) across the pairwise population comparisons was 0.0458. The probability that among 26 genes drawn randomly 3 or more (the same number detected in Figure 1) would overlap a signal of selection in at least one of the pairwise comparisons (as occurred for HGAHs in the Biaka-Mbuti comparison) was p < 0.05.

**Figure 2:**
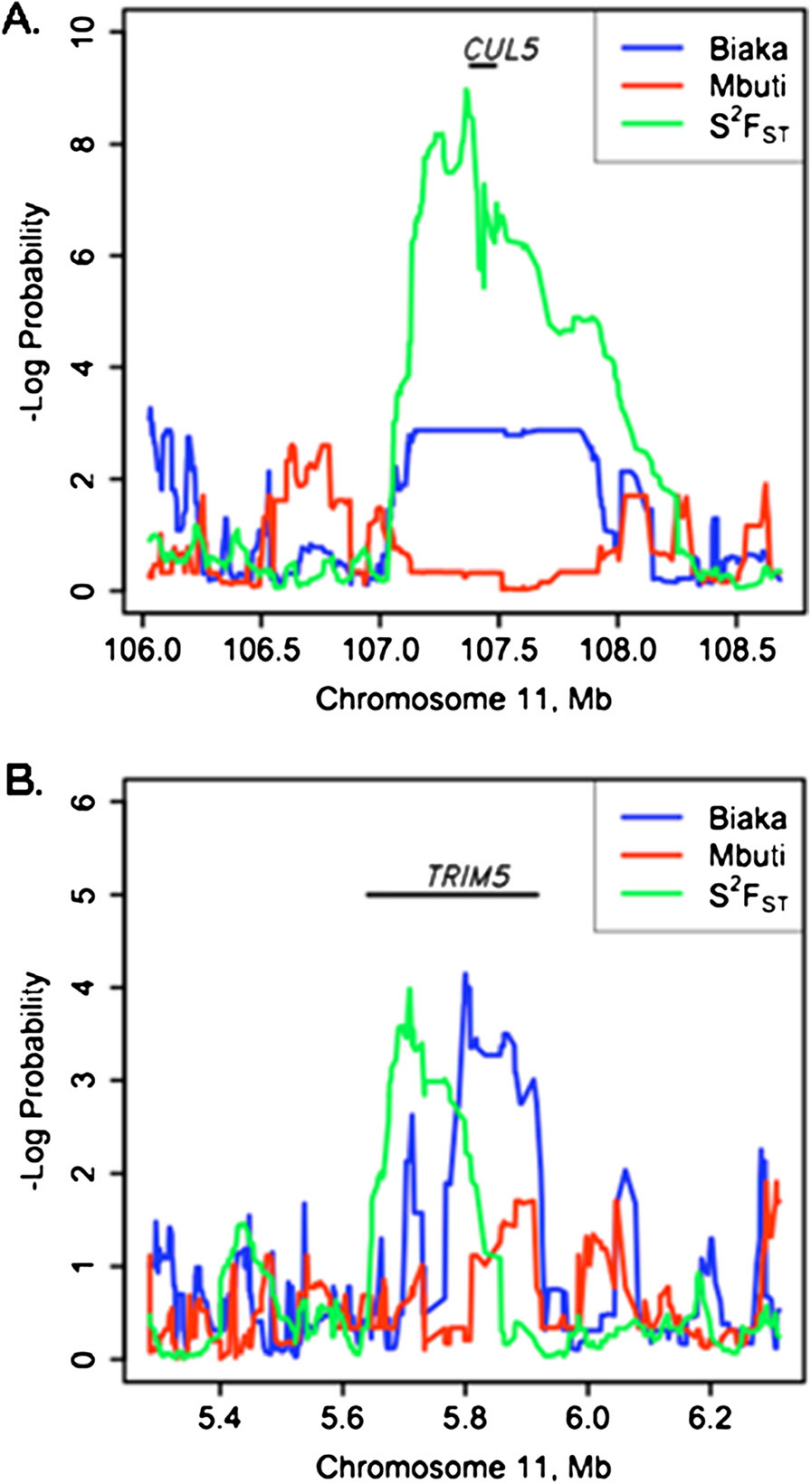
**Signature plots for two host genes associated with HIV-1, under potential recent selection in the Biaka when compared to the Mbuti.** The *x*-axes show chromosomal positions for *CUL5* (panel A) and *TRIM5* (panel B). The *y*-axes map *λ* scores across moving windows of SNPs, used to examine the genome for signatures of selection [19]. Note that the *x*- and *y*-axes for the two genes use different scales. The lines represent values calculated for heterozygosity in Biaka (blue) and Mbuti (red) within populations (low heterozygosity when lines are at high values), and for the variance of *F*_*ST*_ between populations (green; high variance of *F*_*ST*_ when lines are at high values). Signatures of “new” or recent selection for *CUL5* and *TRIM5* in the Biaka are indicated by the overlapping peaks for Biaka heterozygosity and for variance of *F*_*ST*_[19]. The positions of the genes are indicated in the plots.

Both *CUL5* and *TRIM5* showed low values of heterozygosity (plotted as high peaks in Figure 2) in the Biaka, with high values (high peaks in Figure 2) for the variance of *F*_*ST*_ in the genomic regions around each gene in the Biaka-Mbuti comparison (Figure 2). The genomic region around *CUL5*[36] displayed the tenth strongest signal of new selection in Biaka in the pairwise comparison involving the two Pygmy groups. Furthermore, the region under putative selection that included *CUL5* was among the longest (in kb or SNPs) detected using our method (Additional file 1: Figure S3). This may be a further indication of strong or recent selection affecting this genomic region, since strong selection can produce a signature across a longer region of the genome [20]. The genomic region under putative selection around *CUL5* did not appear to have unusually low or high SNP coverage given the length of the region (Additional file 1: Figure S3), an indication that this signal of selection was not distorted by unusual SNP densities. We also looked for previously published SNPs [39] in *CUL5* linked to HIV-1 risk. The protective allele of the *CUL5* SNP rs11212495, located between exons 4 and 5, which is associated with delayed AIDS progression in African Americans [36], was found to be fixed across the Biaka (Table 1).

**Table 1:** Frequencies of alleles protective against HIV-1 in Biaka and Mbuti Pygmies

**Gene**	**SNP**	**Coding Variant**	**Protective Allele**	**Biaka**	**Mbuti**
Non-synonymous coding SNP					
*APOBEC3G*	rs8177832a	H186R	A	0.86**	0.65
*APOBEC3H*	rs139298	K121E	A	**0.25**	0.12
*CXCR6*	rs2234355	E3K	A	0.38*	0.12
*TRIM5*	rs10838525	R136Q	T	**0.11**	0.00
*PARD3B*	rs10185378b	T- > I	T	0.18	0.19
Associated with HIV-1 infection/AIDS progression in an African American cohort					
*CUL5*	rs11212495		A	**1.00**	0.96
*HLA-C*	rs9264942		C	**0.46**	0.27
Associated with HIV-1 infection/AIDS progression in a European cohort					
*RPA12 (ZNRD1)*	rs9261174		C	**0.32**	0.31

Human genes are listed for which a SNP reported to be associated with HIV-1 outcome was genotyped in the human genome diversity panel (HGDP). For each SNP, allele frequencies are listed for the protective allele in Biaka and Mbuti.

Boldface indicates protective alleles for which the Biaka had a higher allele frequency than the Mbuti. For coding variants, the amino acid listed after the position is the protective variant. Within each pygmy population, all alleles were at Hardy-Weinberg equilibrium after Bonferroni correction (*p* < 0.00625).

*Indicates that the allele frequency difference between Biaka and Mbuti is significant (*p* < 0.05) using Fisher's exact test.

**Indicates that the allele frequency difference between Biaka and Mbuti is significant after Bonferroni correction for multiple tests (*p* < 0.00625).

*TRIM5*[40] was also found in a genomic region demonstrating the signature of ‘new’ selection in the Biaka when compared to the Mbuti, as well as when the Biaka were compared with Bantu or Mandenka. *TRIM5* was also in a genomic region displaying a signature of ‘old’ selection when Bantu was compared with Mandenka, which was the only case of a HGAH under potential selection among comparisons that did not involve the Biaka (Figure 1B). For *TRIM5*, in the Biaka-Mbuti comparison the length of the region displaying a signature of selection was shorter and the signature of selection was not as strong as for *CUL5* (Figure 2 and Additional file 1: Figure S3). We looked for previously published SNPs [39] in *TRIM5* associated with HIV-1 risk. We found that a protective T allele in the *TRIM5* SNP rs10838525, which results in a protective codon-changing mutation in the TRIM5-alpha protein [39], was present in 11.4% of Biaka chromosomes (Table 1). This was the highest frequency among African populations, although this allele was more common among non-African than African populations [28].

*PARD3B*[8] was in a genomic region showing the signature of ‘old’ selection when Biaka were compared with Mbuti or Yoruba. For *PARD3B*, a significant correlation has been found between the rare T allele for SNP rs10185378 (a missense mutation in the coding region) and slower AIDS progression [8]. However, this allele was not more common in Biaka (18%) than in other African populations (e.g., 19% in Mbuti; Table 1).

The regions identified as under putative selection in comparisons between Biaka and Mbuti were also examined to identify which of the 2142 genes previously identified as HDFs or as genes that potentially interact with HIV in host cells [10-12] would also overlap genomic signatures of selection. A total of 55 HDFs were found to overlap regions under potential selection in the Biaka, as determined by the Biaka-Mbuti comparison. These genes are listed in Additional file 1: Table S3. HGAHs and HDFs under regions of the genome showing signatures of selection for pairwise comparisons across all five African populations are shown in Additional file 1: Figure S4. In order to minimize the impact of false positives, we had not considered as HGAHs those genes identified by GWAS that were below a genome-wide significance of *p* < 5 × 10^-8^[38]. However, we included all genes identified by GWAS, even those below this cutoff, in a separate analysis. We examined 64 genes found by GWAS to be associated with HIV-1 susceptibility, infection, control and viral set-point as well as AIDS progression from 9 studies [8,9,38,41-45], including genes that did not meet our criteria for HGAHs, and list those genes that overlapped with regions under putative selection between the ten pair-wise comparisons in Additional file 1: Table S4.

We examined other host genes in which SNPs previously associated with protection against HIV-1 had also been genotyped in the HGDP (Table 1). Including the genes mentioned above, there were five genes in which the SNPs were part of the coding region (changing the protein), two genes in which a non-coding protective SNP was associated with a protective effect in African Americans (Table 1); and one gene (*RPA12*) in which a non-coding SNP was associated with a protective effect in Europeans. Of these 8 genes, *PARD3B* was the only one in which Mbuti Pygmies had a greater frequency of protective alleles than the Biaka (Table 1). The protective allele for the non-synonymous coding variant in *APOBEC3G* (rs8177832) [46] was among African populations most common in Biaka (86%) and significantly (*p* < 10^-4^) higher in frequency in Biaka than Mbuti, even after Bonferroni correction. Among sub-Saharan populations, the Biaka had the highest frequencies of alleles associated with protection against HIV-1 for *CUL5* and for *TRIM5*, the two genes showing signatures of new selection in Biaka (Figure 1), as well as for *APOBEC3G*. The protective alleles were also at higher frequencies (though individually not significantly higher) for Biaka than Mbuti for: the non-synonymous coding variant in *APOBEC3H* (rs139298), for an allele in *HLA-C* associated with protection against HIV-1 in both African and European Americans [7,8,47], for an allele in *RPA12* associated with protection against HIV-1 in European Americans [47], and for the non-synonymous protective coding variant rs2234355 of *CXCR6*[48] (Table 1). For 7 of the 8 genes, the SNPs protective against HIV-1 were higher in Biaka than in Mbuti; however, the difference was significant only for *APOBEC3G* and *CXCR6*, and after Bonferroni correction only *APOBEC3G* frequencies were significantly different (Table 1).

We examined results from other tests of selection conducted previously on Biaka genomes. Sabeti et al. have suggested that genomic scans for different signatures of selection are valid across different time scales: tests of selection that examine heterozygosity or population differences can detect more ancient selection than tests relying on linkage disequilibrium [20,49]. Given that signatures of selection persist for different lengths of time, we did not expect a high degree of overlap in the genes detected by our study and those that relied on linkage disequilibrium. With this caveat in mind, we identified HGAHs and HDFs among the genes reported to be under potential selection by Pickrell *et al.*[50] and Lopez Herraez *et al.*[51], who identified genomic signatures of selection in Biaka based on linkage disequilibrium. None of the genes identified by Lopez Herraez *et al.*[51] as under potential selection in the Biaka was a HGAH (Additional file 1: Table S5). However, Pickrell *et al.*[50] had identified *TSG101* as having the eighth strongest signal of potential selection among genes in the Biaka (Additional file 1: Table S5). It is interesting that our own survey also found that *TSG101* was in a genomic region showing the signature of old selection when the Biaka were compared to Mandenka (Figure 1). Variation in *TSG101* has been associated with differences in AIDS progression rates [52], although the SNPs used in that study did not overlap with those used by the current study, so that beneficial or detrimental alleles could not be identified in the Biaka (or other HGDP populations).

Finally, DNA from five individuals each of the Biaka Western Pygmies and the Mbuti Eastern Pygmies was available for sequencing. Regions of five host genes associated with HIV-1 (*CCR2*, *CCR5*, *CUL5*, *TRIM5* and *TSG101*) and two HDFs (*ITGAX* and *OPRM1*) were sequenced in these samples. The sample sizes used would only be sufficient for finding high-frequency polymorphisms; however, we did not detect any novel amino acid variants. Nonetheless, a high degree of sequence diversity at these genes was evident for both Pygmy groups, and we found a novel mutation replacing a rare codon in *CCR5* (heterozygote in one Biaka individual), and numerous SNPs in the promoter regions of each of the HGAHs examined, including novel SNPs and SNPs that would affect transcription factor binding sites (Additional file 1: Table S6). The *CCR2*-64I variant, which is associated with a delay in AIDS progression was found as a heterozygote in one Biaka and one Mbuti individual, although the *CCR5*-Δ32 variant that is in strong linkage disequilibrium with *CCR2*-64I in northern Europeans and their descendants [53] was, as expected, not present in Pygmies.

## Discussion

The prevalence of HIV-1 tends to be lower in African Pygmies than in neighboring communities [54,55], although Pygmies are susceptible to HIV-1, which derives from contact with other human groups [54-57]. Direct transmission of immunodeficiency viruses from non-human primates has not been detected among bushmeat hunters [25,58-60]. But these findings do not rule out historical interspecies transmissions of immunodeficiency viruses from chimpanzees to humans, as at least four independent interspecies transmissions within the past two centuries have occurred (Additional file 1: Figure S1) [14].

Signals of putative selection around four human genes associated with HIV-1 (HGAHs) were detected eight times in pairwise comparisons among five sub-Saharan African populations. Seven of the eight signals entailed comparisons involving the Biaka Pygmy population (Figure 1B). Of the four HGAHs detected by our method as being under putative selection in the Biaka [20], *CUL5* demonstrated the strongest signal of selection (Figure 2 and Additional file 1: Figure S3). *CUL5* codes for the cullin 5 protein, which is recruited by HIV-1 viral infectivity factor (vif) to form a protein complex that functions as an ubiquitin ligase. The complex that includes CUL5 targets and suppresses the anti-viral activity of human apolipoprotein B mRNA editing enzyme APOBEC3G, which is a crucial inhibitor of HIV-1 [61]. *CUL5* polymorphisms in African Americans have been associated with more rapid CD4+ T cell loss following HIV-1 infection [36]. Two SNPs in this gene have been associated with accelerated progression to AIDS while one SNP has been associated with delayed progression to AIDS [36]. We found *CUL5* under strong selection in the Biaka; previous genotyping efforts had included an allele associated with delayed AIDS progression, which we found to be present in 100% of Biaka chromosomes, and 96% of Mbuti chromosomes (Table 1) [28].

The largest alternative splicing protein isoform of *TRIM5*, TRIM5-alpha, is essential for primate retroviral capsid recognition and anti-HIV-1 activity [62]. TRIM5-alpha is a RING domain-E3 ubiquitin ligase that specifically recognizes and prematurely de-coats the HIV-1 capsid to deactivate the virus [40]. It has been demonstrated to have a secondary function of promoting innate immunity signaling after detection of the HIV-1 capsid particle [63]. TRIM5-alpha, in conjunction with the UBC13-UEV1A heterodimer, catalyzes the synthesis of unattached K63-linked ubiquitin chains to activate TAK1 kinase and stimulate AP-1 and NFκ-B signaling. Interaction with the HIV-1 capsid lattice enhances the UBC13-UEV1A-dependent E3 activity of TRIM5-alpha [63]. Interestingly, a rare allele of *TRIM5* has previously been detected in the Baka Western Pygmies of southeastern Cameroon (the current study examined the Biaka Western Pygmies of the Central African Republic). That allele, found as a heterozygote in 4% of the Baka Pygmies results in a truncation of the TRIM5-alpha peptide lacking the functionally important SPRY domain, which would have detrimental effects for individuals infected by HIV-1 [64]. By contrast, in our survey of Pygmies we found that a protective mis-sense mutation in *TRIM5* (rs10838525) [39], which would have beneficial effects for individuals infected by HIV-1, was in the highest frequency in Biaka compared to other African populations (Table 1).

It should be noted that, due to elevated recombination around some important immune response genes, such as *HLA* or *KIR*, our method may not have detected selection in these genes even if it had occurred*.* Additionally, when we examined the HGDP SNP data for SNPs reported as protective against HIV-1, we found that the Biaka had higher frequencies of the protective SNP than the Mbuti for 7 of the 8 genes with protective SNPs (Table 1). Although *APOBEC3G* was not detected as being under selection, an allele that affects the coding region of *APOBEC3G* and is protective against HIV-1 was found to have the highest frequency in Biaka among African populations (Table 1). The protein product of *APOBEC3G* hypermutates the HIV-1 cDNA transcript in the absence of the HIV-1 accessory factor vif. The H186R codon-changing variant has been associated with decreased susceptibility and reduced rate of progression of HIV-1 in African Americans [46]. A higher frequency of protective alleles was found in the Biaka when compared to the Mbuti for three other HGAHs: *APOBEC3H*, *CXCR6*, and *HLA-C* (Table 1). The K121E codon-changing variant of the gene *APOBEC3H*, which encodes a protein that hypermutates HIV-1 transcripts, has been reported to be more effective at restricting HIV-1 *in vitro*[47]. The E3K codon-changing polymorphism in the gene *CXCR6*, which encodes a chemokine that is the primary coreceptor for SIV, has been associated with increased survival time in African Americans with HIV-1 [48]. The protective rs9264942 allele in the major histocompatibility complex gene *HLA-C*, has been associated with decreased viral load in African Americans [7,65].

Several previous studies have reported that African Pygmies carry protective copies of other host genes involved with HIV disease. The CC chemokine ligand 3-like 1 (CCL3L1) protein binds to the HIV coreceptor CCR5 [1,57]. Copy number variation of the *CCL3L1* gene is present across human populations. Higher copy numbers within African-Americans and within European-Americans for the *CCL3L1* gene have been associated with protection against HIV-1, possibly due to competition with the CCR5 receptor used by HIV-1 to enter cells [57]. The Biaka have the second highest copy number of *CCL3L1*, and the Mbuti the fourth highest copy number, among 57 human populations examined across the world [57]. Additionally, the *CCR5* haplotype most commonly found in African Pygmies is associated with delayed HIV-1 disease progression [66]. Models have suggested that among Pygmies, with both high *CCL3L1* copy number and protective *CCR5* alleles, the modern spread of HIV-1 might be minimal due to protective genotypes present within their populations [67]. If selection for resistance to immunodeficiency viruses has affected some human populations in Central Africa, this may have been one factor leading to the low prevalence of HIV-1 in the region relative to other parts of Africa [68].

## Conclusions

In summary, despite small numbers in some studied populations, we found evidence for signatures of recent selection in the Biaka Western Pygmies in genomic regions including *CUL5*, *TRIM5*, and *TSG101* all of which have a functional role in HIV restriction; and for old selection in the genomic region containing *PARD3B,* a gene identified by a GWAS. We also found that among 8 SNPs associated with HIV, the Biaka had the highest frequency of protective alleles for *APOBEC3G*, *CUL5* and *TRIM5* among sub-Saharan Africans, and also had a higher frequency of protective alleles than the Mbuti for 7 of the 8 genes (Table 1). We established that a *CCR2*-64I variant associated with a delay in AIDS progression is carried by some pygmies. Previous researchers have reported a high copy number for *CCL3L1* (associated with favorable HIV outcome) in Pygmies, while Pygmies have high frequencies of the protective, ancestral *CCR5* haplotype [67]. Given these findings, the hypothesis that immunodeficiency viruses may have shaped the genomes of west central African human populations appears to merit further investigation.

## Methods

### Ethical approval

Performed with the approval of the University of Illinois Institutional Review Board (IRB Protocol Number: 09455) and the permission of the University of Illinois Division of Research Safety, the Coriell Institute for Medical Research and the National Institute of General Medical Sciences.

### Human populations

We examined two human populations that have historically resided in the African tropical forest: the Biaka of the Central African Republic are a Western Pygmy population; the Mbuti of the Democratic Republic of Congo are an Eastern Pygmy population. Both groups were part of the Human Genome Diversity Project (HGDP)-*Centre d'Etude du Polymorphisme Humain* (CEPH) Panel, a collection of lymphoblastoid cell lines from 52 geographically diverse human populations [28,37]. In addition to the two populations residing in African tropical forests, we also examined, for comparative purposes, three other human populations within the HGDP from Africa south of the Sahara. These populations, like the Pygmies, exhibit high levels of genetic diversity and low levels of linkage disequilibrium, relative to the non-African populations that have been affected by ancestral founder effect(s) during migration out of Africa [69] The three other sub-Saharan African populations examined were Bantu in Kenya, Mandenka in Senegal, and Yoruba in Nigeria (Figure 1A). Data from the HGDP-CEPH panel were not examined for Bantu outside of Kenya or for the San from Namibia, since sample sizes for these groups were small. Individuals identified as relatives [70] were removed from the dataset; the final dataset contained 91 individuals (2*N* = 182 for each autosome), including Biaka (2*N* = 46), Mbuti (2*N* = 26), Bantu (2*N* = 22) from Kenya, Mandenka (2*N* = 44) and Yoruba (2*N* = 44).

### SNP genotypes

We used the SNP data for the HGDP-CEPH Panel, a dataset containing 938 individuals genotyped on the Illumina 650 K platform [28]. Using the standardized subset of the HGDP data, genotypes for 644,258 autosomal SNPs were available [70]. Chromosomal positions for the SNPs were provided by the HGDP release for NCBI Human Genome build 36.1 and map distances in centimorgans (cM) were calculated using those positions and recombination estimates provided by the HapMap project phase I + II [71].

### Multi-locus test of selection

To examine the genomes for signatures of selection, we applied a previously validated method (developed using datasets unrelated to the current study) [19] that examined regions displaying low heterozygosity within populations and/or high variance in *F*_*ST*_ between populations. By favoring one or few haplotypes at the expense of others, selection reduces the overall level of heterozygosity around a beneficial allele. Thus low heterozygosity in the SNPs surrounding an allele may be a signature of selection. Furthermore, within a population, as haplotype frequencies shift at a genomic region, some alleles will increase and others will decrease in frequency. In the population undergoing selection, some allele frequencies will become more similar, and other allele frequencies will become less similar, to allele frequencies present in a second population not undergoing selection. Thus between two populations relatively high variance of *F*_*ST*_ for alleles at a genomic region may represent a signature of selection [19].

An algorithm that scanned the genome for regions of low heterozygosity within populations and high variance in *F*_*ST*_ between populations [19] was run for each possible pair of African populations. Many iterations of the program were run, each using windows of tandem SNPs varying in size from 5 to 65 SNP loci (2 to 32 SNPs on each flank) [19]. Sparse windows extending more than 1 cM were found not to be present in the genomes, consistent with previous analyses of the HGDP genomes [50]. Applying the method of Oleksyk et al. [19], three values were calculated for each window: median multilocus heterozygosity for each of two populations and the multilocus variance of *F*_*ST*_ between them. The distributions of multilocus values were then evaluated against distributions of ten million multilocus values created by the unrestricted random sampling of SNP windows within the same chromosome, for each size of the sampling window. The quantiles resulting from the comparison with the resampled distribution were calculated for each of the 33 multilocus window sizes, and the most extreme quantile value across windows of different sizes centered on each SNP was reported (*λ*), along with the corresponding window size, as described elsewhere in detail (for datasets unrelated to this study) [19]. Only genomic regions with heterozygosity or *F*_*ST*_ in the most extreme 2.5% tail of their randomized distributions were further examined [19].

The multilocus windows of different sizes were placed in the candidate list of selection if two of the three *λ* scores for a window exceeded the 2.5% cutoff. Windows centered on SNPs where at least two of the three scores were in the top 2.5% were concatenated with overlapping or adjacent (≤ 1 cM) windows fulfilling the same criteria [19] (Additional file 1: Figure S2). The type of selection was inferred as follows [19]: if median heterozygosity in one population and variance of *F*_*ST*_ were both in the top 2.5%, then a signature of “new” selection (post-dating the split between the two populations) was inferred for the population. If the threshold of being in the top 2.5% of genomic values was exceeded by median heterozygosity in both populations, and was exceeded also for the variance in *F*_*ST*_, then a signature of “new” selection was inferred for both populations. If the threshold of being in the top 2.5% of genomic values was exceeded by median heterozygosity in both populations, but was not exceeded by the variance in *F*_*ST*_, then a signature of “old” selection (pre-dating the split between the two populations) was inferred. Since factors other than selection can sometimes affect these calculations [20], and since the history of African pygmies is not well characterized (especially going back more than several thousand years ago), we did not exclude genes identified as under “old” selection, although the focus was on genes under “new” selection in the Biaka.

### Host genes associated with HIV, and HIV dependency factors

Previous studies have identified a set of host (human) genes as being associated with an HIV phenotype (e.g. viral load, progression to AIDS, or CD4 decline) [1,2]. A total of 45 genes clustering at 26 loci have been identified as human genes associated with HIV-1 (HGAHs) in published research reports; these are listed in Additional file 1: Table S2. These 45 genes had been found using candidate-gene or GWAS studies. For GWAS studies, only those with genome wide significance of *p* < 5 × 10^-8^ were further considered, in order to minimize the number of false positives, as suggested by [38]. HIV dependency factors (HDFs) were identified based on published results of siRNA gene knock-down panels designed to uncover genes whose depletion significantly reduces the infection and/or replication ability of HIV [10-12]; or based on a published NIH listing of human host genes that may interact with HIV [72]. We identified HGAHs or HDFs that overlapped completely or partially with the candidate regions identified by our genomic scans comparing pairs of African populations as displaying signatures of selection. Each HGAH and HDF was matched to its chromosomal location using the University of California at Santa Cruz (UCSC) genome browser [73].

We ran a macro written in Visual Basic in Microsoft Excel that identified and calculated allele frequencies for SNPs genotyped in HGAHs from Li et al. [28], Jakobsson et al. [74], and Lopez Herraez et al. [51]. Fisher's exact test (two-tailed; http://www.graphpad.com/quickcalcs/contingency1.cfm) was used to analyze a 2 × 2 contingency table to test whether protective alleles were significantly different between Biaka and Mbuti.

### Permutation tests using randomly chosen genes

Using the R statistical software package [75], we tested how often 26 genes at randomly chosen loci would be found in regions displaying signals of selection, across the ten pair-wise comparisons of populations. We used the list of known and putative genes from the NCBI human genome build 36.3 and sampled 26 genes at random from the list without replacement. For each random sample, the number of genes that overlapped a region with signatures of selection involving the populations was recorded, and this was repeated for 1,000 trials. The number of trials where 7 (the same number as found by this study) or more signals of selection of any type involved the same population was recorded. The number of trials in which 4 (the same number as found by this study) or more of the genes were in a signal of selection between any one pair of populations was also recorded. Although the number of host genes associated with HIV-1 examined by our study was 45, many were tightly linked and they formed 26 separate loci. Since our scan determined which distinct genomic regions were under selection, we considered that the appropriate number of randomly chosen genes for the permutation test should be equal to the number of independent loci, or 26, rather than the full number of genes (including tightly linked genes) of 45. Nonetheless, we did also run a permutation test using 45 randomly chosen genes, within 10% of the size of the 45 HGAHs, in which the number of trials in which 3 or more of the genes overlapped a signal of selection between any one pair of populations was determined, finding also that *p* < 0.05 when 45 randomly drawn genes were used rather than 26.

### Plots for signatures of selection around individual genes

We wrote a program in the R statistical software package [75] to find HGAHs and HDFs with one or more base pairs that overlapped a region with a signature of selection. For individual genes of interest, plots of within-population heterozygosity and between-population variance in *F*_*ST*_ around individual loci were constructed, centering on the *x*-axis a genomic segment that was three times the genetic (cM) size of the region found to display a signature of selection. The *y*-axis corresponded to the maximum value of the *λ* score across all window sizes. The genes of interest were mapped onto the figure using positions based on the UCSC genome browser build hg18 [73].

### Plot comparing regions with signatures of selection

A plot comparing the number of SNPs contained in each region under putative selection and the length in kb of the region under putative selection was constructed using the R software package [75]. For each region under putative selection, genes overlapping with the region were counted, using gene positions provided by the UCSC human genome build 18 [73]. The count of genes was listed in the plot.

### Other genomic scans for selection

We incorporated the results of other selection scans that had examined SNP genotypes among the Biaka Pygmy population. Pickrell et al. (2009) had conducted genomic scans of the HGDP SNP dataset, using integrated haplotype score (iHS) and cross population extended haplotype homozygosity (XP-EHH) tests that relied on a sliding window size of 200 kb to identify genes under regions showing signatures of selection, with increments of 100 kb or 200 kb used for alternative analyses [50]. We identified HGAHs and HDFs among genes identified by Pickrell et al. as under potential selection in the Biaka [50]. Additionally, Lopez Herraez et al. [51] had genotyped five individuals from each HGDP population, including five Biaka, using the Affymetrix GeneChip Human Mapping 500 K array set, concatenating this dataset with that of the Illumina chip [51]. Signatures of selection had been inferred from this data using a modified lnRsb approach, which is similar to the XP-EHH method [51]. We identified HGAHs and HDFs among the genes previously reported by Lopez Herraez et al. as displaying signatures of selection in the Biaka [51].

### PCR and sequencing of genes

We also examined sequence diversity in Pygmies for several human genes associated with HIV-1 (*CCR2*, *CCR5*, *CUL5*, *TRIM5* and *TSG101*), as well as two HDFs (*ITGAX* and *OPRM1*) in 5 Biaka Pygmy and 5 Mbuti Pygmy DNA samples (Coriell Institute for Medical Research, Camden NJ). Sequences and SNPs of each gene were searched and retrieved from NCBI (nucleotide and SNP search) entries and the UCSC Genome Browser [73]. The mutation *CCR2*-64V to *CCR2*-64I delays the progression of AIDS in HIV-1-infected individuals [76]. Thus exon 2 that includes this region was sequenced in *CCR2*. For *CCR5*, exon 4 contains the open reading frame and was sequenced. For *CUL5*, primers were designed to include the putative regions of interaction with HIV-1 vif or with elongins (exons 2–5, and 15–19) [36,77]. Mutation analysis has suggested that both the N-terminal RING and C-terminal SPRY domains of rhesus TRIM5-alpha contribute to its HIV-1 inhibitory activity, thus the regions that code for these domains were sequenced in *TRIM5*[40]. The ubiquitin enzyme-2 variant (UEV) domain [78] in *TSG101* was sequenced since it binds to the p6 domain of the structural Gag protein of HIV-1 [79]. ITGAX (CD11C) is reported to be progressively depleted in HIV-1 infection, and the loss of ITGAX in HIV infection may contribute to AIDS progression [80]. *OPRM1* was sequenced since through the activation of OPRM1, opiate drugs are known to increase HIV-1 replication in macrophages [81]. For all seven genes, promoter regions were also sequenced to examine transcription factor binding sites.

PCR and sequencing primers (Additional file 1: Table S7) were designed using Primer 3.0 [82]. PCR amplifications were performed using 0.4 μM final concentration of each forward and reverse oligonucleotide primer in 1.5 mM MgCl_2_, 200 μM of each dNTP (Life Technologies, Carlsbad CA) with AmpliTaq Gold DNA Polymerase (ABI). The algorithm consisted of an initial 95 °C for 9:45 min; with cycles of 20 sec at 94°C; followed by 30 sec at 60°C (3 cycles); 58°C, 56°C, 54°C, or 52°C (5 cycles each temperature); or 50°C (last 22 cycles); followed by 1 min 30 sec extension at 72°C; with a final extension of 7 min at 72°C. Extension time was reduced if the expected amplicon was small. Amplified fragments were examined on a 1% ethidium bromide stained agarose gel, and purified with Exonuclease I (Life Technologies) and shrimp alkaline phosphatase (Affymetrix Corporation, Santa Clara CA) to remove primers and unincorporated dNTPs prior to sequencing. In some cases (listed in Additional file 1: Table S7), the M13 forward (TGTAAAACGACGGCCAGT) or the M13 reverse sequence (CAGGAAACAGCTATGAC) was added to the 5’ end of PCR primers, to permit the use of M13 forward or reverse primer in sequencing reactions [83]. Sequencing was performed using the BigDye Terminator v3.1 Cycle Sequencing Kit (Life Technologies) with 0.12 μM of primer (PCR and sequencing primers are listed in Additional file 1: Table S7), and the ABI 3730XL capillary sequencer at the University of Illinois Core DNA Sequencing Facility. The software Sequencher 4.5 (Gene Codes Corp., Ann Arbor MI) was used to examine and edit chromatograms. Sequences were deposited in Genbank (accession numbers KC248070-KC248139).

PCR-amplified DNA fragments of the *TSG101*, *CUL5* and *TRIM5* promoter regions were cloned using the TOPO TA Cloning Kit (Life Technologies Corp.) according to the manufacturer’s instructions. Four colonies from each plate were picked, PCR-amplified and sequenced as specified above. For the promoter region and intron 1 of *CUL5* and the promoter region of *TRIM5*, fragment analysis to examine the repeat element size differences was also conducted. For fragment analysis, 2 mM final concentration of MgCl_2_ was used for PCR reaction. PCR products were examined on an agarose gel with ethidium bromide, and electrophoresed on the ABI 3730XL capillary sequencer and analyzed with Genemapper Version 3.7 software (Life Technologies Corp.).

### Transcription factor and rare codon analyses

Transcription factor binding sites in promoter regions were examined using TFSEARCH (http://www.cbrc.jp/research/db/TFSEARCH.html), which uses the TRANSFAC database [84]. The tRNA effect of the nucleotide substitutions was examined by calculating the rare codon using the Rare Codon Caltor from the University of California (http://people.mbi.ucla.edu/sumchan/caltor.html).

## Competing interests

The authors have declared that no competing interests exist.

## Authors’ contributions

ALR conceived of the study. ALR, KZ, TKO and CAW designed the study. KZ and CAW identified genes of interest. YI conducted sequencing and sequence analyses. KZ, TKO and ALR contributed to bioinformatics searches and statistical analyses. All authors contributed to writing the manuscript and approved of the final manuscript.

## Funding

Funded by a grant from the Bill & Melinda Gates Foundation through the Grand Challenges Exploration Initiative. The content of this publication does not necessarily reflect the views or policies of the Department of Health and Human Services, nor does mention of trade names, commercial products, or organizations imply endorsement by the U.S. Government. This project has been funded in part with federal funds from the National Cancer Institute, National Institutes of Health, under contract HHSN26120080001E.

## Supplementary Material

Additional file 1 **Table S1.** Proportion of autosomes showing signatures of selection for pairs of populations. Description: “New” selection indicates selection after the divergence of the two populations. “Old” selection occurred prior to the divergence of the two populations. Proportions are based on physical size (bp not cM) of the genome. The method of Oleksyk and colleagues (2008) [19] was applied to the human genome diversity panel African populations. **Table S2**. Locations and descriptions of HIV associated host genes. Description: Genomic locations listed are based on UCSC Genome Browser build hg18 [73]. **Table S3**. Human genes associated with HIV-1 (HGAHs) and HIV dependency factors (HDFs) under potential selection in Biaka, when Biaka and Mbuti genomes are compared. Description: HDFs and HGAHs that overlapped with genomic regions found under putative selection by applying the method of Oleksyk et al. [19] were sorted by the type of selection (recent in Biaka, recent in both Biaka and Mbuti, or old in Biaka and Mbuti) then ranked by the strength of selection measured by the product of the *λ* values that were used to assign the type of selection (as noted in methods). Genes that are HGAHs are in boldface. **Table S4**. Genes from GWAS studies in regions of selection. **Table S5**. Human genes associated with HIV-1 (HGAHs) and HIV dependency factors (HDFs) among genes previously reported as under putative selection in the Biaka. **Table S6**. SNPs in genes sequenced in Pygmies. **Table S7**. Oligonucleotide primers used for PCR or sequencing of genes. **Figure S1**. Geographic distribution of chimpanzee subspecies and phylogenetic relationship of strains of immunodeficiency viruses. **Figure S2**. Identification of types of selection based on genomic patterns. **Figure S3**. Length in kb of genomic regions under putative selection, with number of SNPs and genes within each region. **Figure S4**. List of all HGAHs and HDFs found in regions with signatures of selection for all pairwise comparisons. (PDF 2979 kb) Click here for file
