# Supplementary material for: Evidence for selection at HIV host susceptibility genes in a West Central African human population
Source: BMC Evol Biol. 2012 Dec 6;12:237. doi: 10.1186/1471-2148-12-237 (PMC3537702; doi:10.1186/1471-2148-12-237)
Supplement: Additional file 1 — Table S1. Proportion of autosomes showing signatures of selection for pairs of populations. Description: “New” selection indicates selection after the divergence of the two populations. “Old” selection occurred prior to the divergence of the two populations. Proportions are based on physical size (bp not cM) of the genome. The method of Oleksyk and colleagues (2008) [19] was applied to the human genome diversity panel African populations. Table S2. Locations and descriptions of HIV associated host genes. Description: Genomic locations listed are based on UCSC Genome Browser build hg18 [73]. Table S3. Human genes associated with HIV-1 (HGAHs) and HIV dependency factors (HDFs) under potential selection in Biaka, when Biaka and Mbuti genomes are compared. Description: HDFs and HGAHs that overlapped with genomic regions found under putative selection by applying the method of Oleksyk et al. [19] were sorted by the type of selection (recent in Biaka, recent in both Biaka and Mbuti, or old in Biaka and Mbuti) then ranked by the strength of selection measured by the product of the λ values that were used to assign the type of selection (as noted in methods). Genes that are HGAHs are in boldface. Table S4. Genes from GWAS studies in regions of selection. Table S5. Human genes associated with HIV-1 (HGAHs) and HIV dependency factors (HDFs) among genes previously reported as under putative selection in the Biaka. Table S6. SNPs in genes sequenced in Pygmies. Table S7. Oligonucleotide primers used for PCR or sequencing of genes. Figure S1. Geographic distribution of chimpanzee subspecies and phylogenetic relationship of strains of immunodeficiency viruses. Figure S2. Identification of types of selection based on genomic patterns. Figure S3. Length in kb of genomic regions under putative selection, with number of SNPs and genes within each region. Figure S4. List of all HGAHs and HDFs found in regions with signatures of selection for all pairwise comparisons. (PDF 2979 kb) [file 1471-2148-12-237-S1.pdf]

Evidence for selection at HIV host susceptibility genes in a West Central African human population

Kai Zhao (1), Yasuko Ishida (1), Taras K. Oleksyk (2), Cheryl A. Winkler (3), Alfred L. Roca (1,4)

(1) Department of Animal Sciences, University of Illinois at Urbana-Champaign, Urbana, Illinois 61801 USA
(2) Department of Biology, University of Puerto Rico at Mayaguez, Mayaguez, Puerto Rico 00681
(3) Basic Research Laboratory, Center for Cancer Research, NCI, SAIC-Frederick, Frederick National Laboratory for Cancer Research, Frederick, Maryland 21702 USA
(4) Institute for Genomic Biology, University of Illinois at Urbana-Champaign, Urbana, Illinois 61801 USA

Additional file 1

Supplementary Table S1. Proportion of autosomes showing signatures of selection for pairs of populations.

Genomes compared		New selection in				
Population 1	Population 2	Population 1	Population 2	Both 1 and 2	Old selection	Total
Biaka	Mbuti	0.33%	0.40%	0.22%	0.63%	1.58%
Biaka	Bantu	0.30%	0.39%	0.48%	0.61%	1.77%
Biaka	Mandenka	0.42%	0.35%	0.80%	0.58%	2.15%
Biaka	Yoruba	0.49%	0.46%	0.67%	0.57%	2.19%
Mbuti	Bantu	0.39%	0.48%	0.61%	0.37%	1.86%
Mbuti	Mandenka	0.33%	1.28%	0.35%	0.37%	2.32%
Mbuti	Yoruba	0.76%	0.37%	0.33%	0.37%	1.82%
Bantu	Mandenka	0.26%	0.28%	0.45%	1.33%	2.32%
Bantu	Yoruba	0.31%	0.45%	0.19%	0.99%	1.94%
Mandenka	Yoruba	0.43%	0.39%	0.21%	1.52%	2.55%

“New” selection indicates selection after the divergence of the two populations. “Old” selection occurred prior to the divergence of the two populations. Proportions are based on physical size (bp not cM) of the genome. The method of Oleksyk and colleagues (2008) [1] was applied to the human genome diversity panel African populations.

REFERENCE

1. Oleksyk TK, Zhao K, Vega FMDL, Gilbert DA, O'Brien SJ, Smith MW: **Identifying selected regions from heterozygosity and divergence using a light-coverage genomic dataset from two human populations.** *PLoS One* 2008, **3:**e1712.

Supplementary Table S2. Locations and descriptions of HIV associated host genes.

	Gene	Location	Function	References
1	*APOBEC3B*	22:37708350-37718729	A homozygous deletion of the *APOBEC3B* gene is associated with increased risk of HIV-1 acquisition, progression to AIDS, and increased viral set point.	[1]
2	*APOBEC3G*	22:37803081-37813694	A codon-changing variant of *APOBEC3G* increases the rate of AIDS progression.	[2, 3]
3	*APOBEC3H*	22:37823235-37830016	Several SNPs in *APOBEC3H* are correlated with increased Vif-resistant anti-HIV activity.	[4]
4	*CCL2*	17:29606408-29608333	Haplotypes of the *CCL2-CCL7-CCL11* gene cluster are associated with resistance to HIV-1 infection. Additionally, a coding variant in *CCL2* delays the onset of AIDS.	[5, 6]
	CCL7	17:29621352-29623369		
	CCL11	17:29636799-29639312		
5	*CCL3*	17:31439715-31441619	Haplotypes of the *CCL3-CCL4-CCL18* gene cluster are associated with accelerated AIDS progression.	[7]
	CCL4	17:31455332-31457127		
	CCL18	17:31415755-31422954		
6	*CCL3L1*	17:31546381-31548269<br>17:31647955-31649843	Increased copy number of *CCL3L1* has been shown to be protective against HIV-1 infection and AIDS progression, although the association has been disputed. The human genome reference sequence has two copies of the gene; individuals have been genotyped with zero to ten copies of this gene.	[8-10]

7	*CCL5*	17:31222608-31231490	Variants in *CCL5* have been shown to increase susceptibility to HIV-1, or to increase or decrease the rate of AIDS progression.	[11, 12]
8	*CCR2*	3:46370238-46377429	The *CCR2*-64I coding variant has a protective effect against AIDS progression	[13, 14]
9	*CCR5*	3:46386636-46392701	A 32 base-pair deletion results in a truncated HIV-1 coreceptor, which prevents HIV-1 acquisition and delays AIDS. SNPs in *CCR5* and its promoter regions are associated with both protection against AIDS and increased susceptibility.	[15, 16]
10	*CUL5*	11:107384617-107483698	The CUL5 protein is recruited by HIV-1 as part of a complex to disable the human *APOBEC3G* antiviral mechanism. SNPs in *CUL5* have been associated with both accelerated and delayed AIDS progression.	[17]
11	*CXCL12*	10:44185610-44200548	A polymorphism in the *CXCL12* 3' UTR increases the level of *CXCL12* mRNA and enhances mRNA stability of *CXCL12*. The protein is protective against AIDS.	[18, 19]
12	*CXCR6*	3:45959976-45964849	A codon-changing variant in *CXCR6* accelerates AIDS progression.	[20, 21]
13	*DC-SIGN (CD209)*	19:7710881-7718406	A SNP in *DC-SIGN* has been associated with increased risk of perinatal transfer of HIV-1.	[22]
14	*HLA-A*	6:29963507-30085130	Several variants in the *HLA* region have been shown to affect rates of AIDS progression and HIV transmission.	[23-26]
	HLA-B	6:31429627-31432968		
	HLA-C	6:31344507-31347834		
15	*IFNG*	12:66834816-66839788	A SNP in *INFG* is associated with accelerated AIDS progression.	[27]

16	*IL10*	1: 205007570-205012659	A SNP in the 5' promoter region of *IL10* is linked to enhanced HIV-1 infection and accelerated AIDS progression.	[28]
17	*IL10RA*	11:117362318-117377404	A SNP in *IL10RA* is associated with changes in the rate of AIDS progression.	[29]
18	*IL10RB*	22:33542883-33591390	Several SNPs in *IL10RB* are associated with changes in HIV-1 infection and AIDS progression.	[29]
19	*KIR2DL1*	19:59973076-59987590	The *KIR* locus on chromosome 19 comprises of a family of homologous genes that are receptors for HLA ligands. Interactions between *KIR* and *HLA* genotypes affect HIV infectivity and AIDS progression.<br><br>The human genome reference sequence does not contain some *KIR* genes and thus their positions are not available.	[30, 31]
	KIR2DL2	19:59942764-59987590		
	KIR2DL3	19:59927795-59956316		
	KIR2DL4	19:59989604-60017784		
	KIR2DL5			
	KIR2DL5B			
	KIR2DP1			
	KIR2DS2	19:59927795-60051835		
	KIR2DS3			
	KIR2DS5			
	KIR3DL1	19:59989604-60034045		
	KIR3DL3	19:59927795-60001550		

	KIR3DP1	19:59927795-60001550		
	KIR3DS1			
20	*LEDGF*	9:15454064-15501003	Haplotypes of *LEDGF* are associated with variation in HIV-1 infection and AIDS progression.	[32]
21	*PARD3B*	2:205118760-206188782	SNPs in *PARD3B* have been associated with variance in AIDS progression in a genome-wide association study.	[33]
22	*PP1A*	11:66922227-66925651	Several SNPs found in *PP1A* are associated with accelerated AIDS progression.	[34]
23	*PROX1*	1:212228482-212276385	A haplotype in *PROX1* is associated with delayed AIDS progression.	[35]
24	*RPA12 (ZNRD1)*	6:30137014-30140665	A genome-wide association study found significant association between *ZNRD1* and variation in AIDS progression.	[36]
25	*TRIM5*	11:5641363-5916425	Several SNPs in *TRIM5* are associated with differential HIV-1 infection rates. Additionally, a null allele has been found in Baka pygmies that lacks the crucial SPRY domain in the encoded TRIM5-alpha protein.	[37, 38]
26	*TSG101*	11:18458433-18505065	Three haplotypes identified in *TSG101* have been associated with different rates of AIDS progression.	[39]

References

1. An P, Johnson R, Phair J, Kirk GD, Yu XF, Donfield S, Buchbinder S, Goedert JJ, Winkler CA: ***APOBEC3B* deletion and risk of HIV-1 acquisition.** *J Infect Dis* 2009, **200:**1054-1058.
2. An P, Bleiber G, Duggal P, Nelson G, May M, Mangeat B, Alobwede I, Trono D, Vlahov D, Donfield S, et al: ***APOBEC3G* genetic variants and their influence on the progression to AIDS.** *J Virol* 2004, **78:**11070-11076.
3. Malim MH: **APOBEC proteins and intrinsic resistance to HIV-1 infection.** *Philos Trans R Soc Lond B Biol Sci* 2009, **364:**675-687.
4. Harari A, Ooms M, Mulder LCF, Simon V: **Polymorphisms and splice variants influence the antiretroviral activity of human *APOBEC3H*.** *J Virol* 2009, **83:**295-303.
5. Modi WS, Goedert JJ, Strathdee S, Buchbinder S, Detels R, Donfield S, O'Brien SJ, Winkler C: ***MCP-1-MCP-3-Eotaxin* gene cluster influences HIV-1 transmission.** *AIDS* 2003, **17:**2357-2365.
6. Smith M, Carrington M, Winkler C, Lomb D: ***CCR2* chemokine receptor and AIDS progression.** *Nat Med* 1997, **3:**1052-1053.
7. Modi WS, Lautenberger J, An P, Scott K, Goedert JJ, Kirk GD, Buchbinder S, Phair J, Donfield S, O'Brien SJ, Winkler C: **Genetic variation in the *CCL18-CCL3-CCL4* chemokine gene cluster influences HIV Type 1 transmission and AIDS disease progression.** *Am J Hum Genet* 2006, **79:**120-128.
8. Gonzalez E, Kulkarni H, Bolivar H, Mangano A, Sanchez R, Catano G, Nibbs RJ, Freedman BI, Quinones MP, Bamshad MJ, et al: **The influence of *CCL3L1* gene-containing segmental duplications on HIV-1/AIDS susceptibility.** *Science* 2005, **307:**1434-1440.
9. Kulkarni H, Marconi VC, Agan BK, McArthur C, Crawford G, Clark RA, Dolan MJ, Ahuja SK: **Role of *CCL3L1-CCR5* genotypes in the epidemic spread of HIV-1 and evaluation of vaccine efficacy.** *PloS one* 2008, **3:**e3671.
10. Bhattacharya T, Stanton J, Kim EY, Kunstman KJ, Phair JP, Jacobson LP, Wolinsky SM: ***CCL3L1* and HIV/AIDS susceptibility.** *Nat Med* 2009, **15:**1112-1115.
11. An P, Nelson GW, Wang L, Donfield S, Goedert JJ, Phair J, Vlahov D, Buchbinder S, Farrar WL, Modi W, et al: **Modulating influence on HIV/AIDS by interacting *RANTES* gene variants.** *Proc Natl Acad Sci U S A* 2002, **99:**10002-10007.
12. Liu H, Chao D, Nakayama EE, Taguchi H, Goto M, Xin X, Takamatsu JK, Saito H, Ishikawa Y, Akaza T, et al: **Polymorphism in *RANTES* chemokine promoter affects HIV-1 disease progression.** *Proc Natl Acad Sci U S A* 1999, **96:**4581-4585.
13. Smith MW: **Contrasting genetic influence of *CCR2* and *CCR5* variants on HIV-1 infection and disease progression.** *Science* 1997, **277:**959-965.
14. Mariani R, Wong S, Mulder LC, Wilkinson DA, Reinhart AL, LaRosa G, Nibbs R, O'Brien TR, Michael NL, Connor RI, et al: **CCR2-64I polymorphism is not associated with altered CCR5 expression or coreceptor function.** *Journal of virology* 1999, **73:**2450-2459.

15. An P, Martin MP, Nelson GW, Carrington M, Smith MW, Gong K, Vlahov D, O'Brien SJ, Winkler Ca: **Influence of *CCR5* promoter haplotypes on AIDS progression in African-Americans.** *AIDS* 2000, **14:**2117-2122.
16. Dean M, Carrington M, Winkler C, Huttley GA, Smith MW, Allikmets R, Goedert JJ, Buchbinder SP, Vittinghoff E, Gomperts E, et al: **Genetic restriction of HIV-1 infection and progression to AIDS by a deletion allele of the *CKR5* structural gene. Hemophilia Growth and Development Study, Multicenter AIDS Cohort Study, Multicenter Hemophilia Cohort Study, San Francisco City Cohort, ALIVE Study.** *Science* 1996, **273:**1856-1862.
17. An P, Duggal P, Wang LH, O'Brien SJ, Donfield S, Goedert JJ, Phair J, Buchbinder S, Kirk GD, Winkler CA: **Polymorphisms of *CUL5* are associated with CD4+ T cell loss in HIV-1 infected individuals.** *PLoS Genet* 2007, **3:**e19.
18. Garcia-Moruja C, Rueda P, Torres C, Alcamí J, Luque F, Caruz A: **Molecular phenotype of *CXCL12beta* 3'UTR G801A polymorphism (rs1801157) associated to HIV-1 disease progression.** *Curr HIV Res* 2009:384-389.
19. Winkler C, Modi WD, Smith MW, Nelson GW, Wu X, Carrington M, Dean M, Honjo T, Tashiro K, Yabe D, et al: **Genetic restriction of AIDS pathogenesis by an *SDF-1* chemokine gene variant.** *Science* 1998, **279:**389-393.
20. Manfredi R, Calza L, Chiodo F: **The role of a novel antiretroviral class (fusion inhibitors) in the treatment of advanced HIV infection. Preliminary experience with enfuvirtide.** *Recenti Prog Med* 2005, **96:**124-130.
21. Duggal P, An P, Beaty TH, Strathdee Sa, Farzadegan H, Markham RB, Johnson L, O'Brien SJ, Vlahov D, Winkler Ca: **Genetic influence of *CXCR6* chemokine receptor alleles on PCP-mediated AIDS progression among African Americans.** *Genes Immun* 2003, **4:**245-250.
22. Martin MP, Lederman MM, Hutcheson HB, Goedert JJ, Nelson GW, Kooyk Yv, Detels R, Buchbinder S, Hoots K, Vlahov D, et al: **Association of *DC-SIGN* promoter polymorphism with increased risk for parenteral, but not mucosal, acquisition of human immunodeficiency virus type 1 infection.** *J Virol* 2004, **78:**14053-14056.
23. Carrington M: ***HLA* and HIV-1: Heterozygote advantage and B*35-Cw*04 disadvantage.** *Science* 1999, **283:**1748-1752.
24. Gao X, Nelson GW, Karacki P, Martin MP, Phair J, Kaslow R, Goedert JJ, Buchbinder S, Hoots K, Vlahov D, et al: **Effect of a single amino acid change in MHC class I molecules on the rate of progression to AIDS.** *N Engl J Med* 2001, **344:**1668-1675.
25. Mackelprang RD, John-Stewart G, Carrington M, Richardson B, Rowland-jones S, Gao X, Mbori-ngacha D, Mabuka J, Lohman-payne B, Farquhar C: **Maternal HLA homozygosity and mother-Child *HLA c*oncordance increase the risk of vertical transmission of HIV-1.** *J Infect Dis* 2008, **197:**1156-1161.
26. Tang J, Shao W, Yoo YJ, Brill I, Mulenga J, Hunter E, Kaslow RA: **Human leukocyte antigen class I genotypes in relation to heterosexual HIV type 1 transmission within discordant couples.** *J Immunol* 2008, **181:**2626-2635.
27. An P, Vlahov D, Margolick JB, Phair J, O'Brien TR, Lautenberger J, O'Brien SJ, Winkler Ca: **A tumor necrosis factor-alpha-inducible promoter variant of interferon-gamma accelerates CD4+ T cell depletion in human immunodeficiency virus-1-infected individuals.** *J Infect Dis* 2003, **188:**228-231.

28. Shin HD, Winkler C, Stephens JC, Bream J, Young H, Goedert JJ, O'Brien TR, Vlahov D, Buchbinder S, Giorgi J, et al: **Genetic restriction of HIV-1 pathogenesis to AIDS by promoter alleles of *IL10*.** *Proc Natl Acad Sci U S A* 2000, **97:**14467-14472.
29. Shrestha S, Wiener HW, Aissani B, Song W, Shendre A, Wilson CM, Kaslow Ra, Tang J: **Interleukin-10 (*IL-10*) pathway: genetic variants and outcomes of HIV-1 infection in African American adolescents.** *PLoS ONE* 2010, **5:**e13384.
30. Carrington M, Martin MP, Bergen Jv: ***KIR-HLA* intercourse in HIV disease.** *Trends Microbiol* 2008, **16:**620-627.
31. Martin MP, Carrington M: ***KIR* locus polymorphisms: genotyping and disease association analysis.** *Methods Mol Biol* 2008, **415:**49-64.
32. Madlala P, Gijsbers R, Christ F, Hombrouck A, Werner L, Mlisana K, An P, Karim SSA, Winkler CA, Debyser Z, others: **Association of polymorphisms in the *LEDGF*/p75 gene (*PSIP1*) with susceptibility to HIV-1 infection and disease progression.** *AIDS* 2011.
33. Troyer JL, Nelson GW, Lautenberger Ja, Chinn L, McIntosh C, Johnson RC, Sezgin E, Kessing B, Malasky M, Hendrickson SL, et al: **Genome-wide association study implicates *PARD3B*-based AIDS restriction.** *J Infect Dis* 2011, **203:**1491-1502.
34. An P, Wang LH, Hutcheson-Dilks H, Nelson G, Donfield S, Goedert JJ, Rinaldo CR, Buchbinder S, Kirk GD, O'Brien SJ, Winkler Ca: **Regulatory polymorphisms in the cyclophilin A gene, *PPIA*, accelerate progression to AIDS.** *PLoS Pathog* 2007, **3:**e88.
35. Herbeck JT, Gottlieb GS, Winkler CA, Nelson GW, An P, Maust BS, Wong KG, Troyer JL, Goedert JJ, Kessing BD, others: **Multistage genomewide association study identifies a locus at 1q41 associated with rate of HIV-1 disease progression to clinical AIDS.** *J Infect Dis* 2010, **201:**618.
36. Fellay J, Shianna KV, Ge D, Colombo S, Ledergerber B, Weale M, Zhang K, Gumbs C, Castagna A, Cossarizza A, others: **A whole-genome association study of major determinants for host control of HIV-1.** *Science* 2007, **317:**944.
37. Javanbakht H, An P, Gold B, Petersen DC, O'Huigin C, Nelson GW, O'Brien SJ, Kirk GD, Detels R, Buchbinder S, et al: **Effects of human TRIM5-alpha polymorphisms on antiretroviral function and susceptibility to human immunodeficiency virus infection.** *Virology* 2006, **354:**15-27.
38. Torimiro JN, Javanbakht H, Diaz-Griffero F, Kim J, Carr JK, Carrington M, Sawitzke J, Burke DS, Wolfe ND, Dean M, Sodroski J: **A rare null allele potentially encoding a dominant-negative TRIM5alpha protein in Baka pygmies.** *Virology* 2009, **391:**140-147.
39. Bashirova AA, Bleiber G, Qi Y, Hutcheson H, Yamashita T, Johnson RC, Cheng J, Alter G, Goedert JJ, Buchbinder S, et al: **Consistent effects of *TSG101* genetic variability on multiple outcomes of exposure to human immunodeficiency virus type 1.** *J Virol* 2006, **80:**6757-6763.

Supplementary Table S3. Human genes associated with HIV-1 (HGAHs) and HIV dependency factors (HDFs) under potential selection in Biaka, when Biaka and Mbuti genomes are compared.

Gene	Position	Rank	Type of Selection
HLA-DOB	6:32874832-32906709	1	Recent in Biaka
ATM	11:107122467-107851313	2	Recent in Biaka
CUL5	11:107122467-107851313	2	Recent in Biaka
FGD6	12:93915516-93996220	4	Recent in Biaka
KPNA3	13:48904591-49229993	5	Recent in Biaka
SESTD1	2:179590507-179848028	6	Recent in Biaka
CHST10	2:100307191-100387056	6	Recent in Biaka
PTPRB	12:69270162-69270286	8	Recent in Biaka
TRIM22	11:5712786-5712786	9	Recent in Biaka
TRIM5	11:5712786-5712786	9	Recent in Biaka
DOCK10	2:225501079-225511799	11	Recent in Biaka
AGBL5	2:27113973-27201533	12	Recent in Biaka
CGREF1	2:27113973-27201533	12	Recent in Biaka
DHX33	17:5292515-5307671	14	Recent in Biaka
CHST9	18:22941322-23017276	15	Recent in Biaka
TMEM163	2:135048825-135151858	16	Recent in Biaka
ATG16L2	11:72131498-72483251	1	Recent in Biaka & Mbuti
LAMC1	1:181319208-181444077	2	Recent in Biaka & Mbuti
LAMC2	1:181319208-181444077	2	Recent in Biaka & Mbuti
GPX5	6:28541114-28808331	3	Recent in Biaka & Mbuti
GPX6	6:28541114-28808331	3	Recent in Biaka & Mbuti
IL2	4:123320561-123721113	5	Recent in Biaka & Mbuti
ITGAX	16:31273517-31535883	6	Recent in Biaka & Mbuti
SNRPD1	19:50748340-50902205	8	Old in Biaka & Mbuti
SNRPD2	19:50748340-50902205	8	Old in Biaka & Mbuti
BRCA1	17:38465179-38497526	10	Old in Biaka & Mbuti
POLR2J	7:101695725-102411428	11	Old in Biaka & Mbuti
COG4	16:68486638-69167724	12	Old in Biaka & Mbuti
ST3GAL2	16:68486638-69167724	12	Old in Biaka & Mbuti
KCNMB2	3:179681525-179743880	14	Old in Biaka & Mbuti
HMGCR	5:74482560-74844173	15	Old in Biaka & Mbuti
PDE4B	1:66387855-66456040	16	Old in Biaka & Mbuti
EGF	4:111029826-111105755	17	Old in Biaka & Mbuti
FBXO18	10:5984449-6002641	18	Old in Biaka & Mbuti
SLC1A2	11:35349177-35384951	19	Old in Biaka & Mbuti
RNF10	12:119491112-119570267	20	Old in Biaka & Mbuti
PLCB1	20:8531414-8555393	21	Old in Biaka & Mbuti
IL8	4:74807862-74943784	22	Old in Biaka & Mbuti

Gene	Position	Rank	Type of Selection
TAT	16:70148170-70227125	23	Old in Biaka & Mbuti
NCOR2	12:123549569-123549878	24	Old in Biaka & Mbuti
CLOCK	4:56018354-56033550	25	Old in Biaka & Mbuti
OPRM1	6:154403712-154456266	26	Old in Biaka & Mbuti
PDE3A	12:20525183-20534841	27	Old in Biaka & Mbuti
COPB1	11:14361401-14560124	28	Old in Biaka & Mbuti
PSMA1	11:14361401-14560124	28	Old in Biaka & Mbuti
CLNS1A	11:76670312-77272002	30	Old in Biaka & Mbuti
PAK1	11:76670312-77272002	30	Old in Biaka & Mbuti
PSMB2	1:35743595-35940043	32	Old in Biaka & Mbuti
KCNMA1	10:78894205-78905202	33	Old in Biaka & Mbuti
RPL5	1:92738557-93076039	34	Old in Biaka & Mbuti
GABRP	5:170173072-170173072	35	Old in Biaka & Mbuti
PSMD13	11:227087-232112	36	Old in Biaka & Mbuti
VAV3	1:107901413-107916668	37	Old in Biaka & Mbuti
ITGB1	10:33228108-33282407	38	Old in Biaka & Mbuti
PARD3B	2:205683626-205683626	39	Old in Biaka & Mbuti

HDFs and HGAHs that overlapped with genomic regions found under putative selection by applying the method of Oleksyk *et al.* [1] were sorted by the type of selection (recent in Biaka, recent in both Biaka and Mbuti, or old in Biaka and Mbuti) then ranked by the strength of selection measured by the product of the λ values that were used to assign the type of selection (as noted in methods). Genes that are HGAHs are in boldface.

REFERENCE

1. Oleksyk TK, Zhao K, Vega FMDL, Gilbert DA, O'Brien SJ, Smith MW: **Identifying selected regions from heterozygosity and divergence using a light-coverage genomic dataset from two human populations.** *PLoS One* 2008, **3:**e1712.

Supplementary Table S4. Genes from GWAS studies in regions of selection. Genes associated with HIV-1 susceptibility, infection, control and viral set-point as well as AIDS progression from 9 genome-wide association studies (GWAS) are listed. Comparisons between two populations are paired in columns. An "N" indicates that the gene overlaps with a putative signature of new selection in that population and an "O" indicates that the gene overlaps with a putative signature of old selection.

Gene	Biaka	Mbuti	Biaka	Bantu	Mbuti	Bantu	Biaka	Yoruba	Mbuti	Yoruba	Biaka	Mandenka	Mbuti	Mandenka	Bantu	Mandenka	Bantu	Yoruba	Mandenka	Yoruba	Reference(s)
AC023798.16																					[1]
ACTR3BP6																					[2]
ADAM18																					[2]
ADH5P4																					[2]
AOAH																					[1]
BTNL2															N	N					[5]
BUD13																					[3]
CCRL2																					[4]
CDSN																					[5]
CMTM8																					[2]
CYP7B1																					[2]
DDR1									N	N			N	N							[5]
DGKI																					[4]
DNAJC5B																					[2]
DYRK1A																					[6]
EPHA5																N					[2]
EVI5L																					[1]
FAM174B																					[3]
GALNT14																					[2]
GLRX3																					[3]
GLTSCR1																					[2]
GNPDA2																					[2]
GPC5																		N			[1]
GRIN21																					[2]
H2AFY																					[4]
HCP5																					[7], [5]
HCRTR2																					[5]
HIST1H4A																	O	O			[2]
HLA-B																					[7], [1]
HLA-C																					[7], [5]
IL32																					[2]
LRRC58																					[2]
LTF																					[4]
MAD2L1																					[2]
MCM8																					[1]
MEPE																					[2]
MICA																					[7]
NAV2															O	O	O	O			[2]
NBPF14																					[4]
NEDD9																					[2]
ODZ4																				N	[1]
PARD3B	O	O					O	O													[4]
PC					O	O															[2]
PPP3CC																					[1]
PRKG2															N	N			O	O	[2]
PSORS1C3																					[7]
RP11-100A16.1																					[1]
RPH3AL																					[4]

	Biaka Mbuti	Biaka Bantu	Mbuti Bantu	Biaka Yoruba	Mbuti Yoruba	Biaka Mandenka	Mbuti Mandenka	Bantu Mandenka	Bantu Yoruba	Mandenka Yoruba	
RPL21P126											[3]
RPL4P5											[3]
RPS20P23											[8]
RXRG					N		N				[8]
SLCO5A1									N		[5]
SNORD52											[5]
SORBS3											[1]
SOX5		N	N						N		[8]
TGFBRAP1											[8]
TNXB											[5]
TRIM10											[5]
VEGFC			N								[2]
WASF5P											[9]
ZDHHC19											[3]
ZFP90											[2]
ZNRD1											[5], [9]

REFERENCES:

1. Pelak K, Goldstein DB, Walley NM, Fellay J, Ge D, Shianna KV, Gumbs C, Gao X, Maia JM, Cronin KD, et al: **Host determinants of HIV-1 control in African Americans.** *J Infect Dis 2010* , **201**:1141-1149.
2. Lingappa JR, Dumitrescu L, Zimmer SM, Lynfield R, McNicholl JM, Messonnier NE, Whitney CG, Crawford DC: **Identifying host genetic risk factors in the context of public health surveillance for invasive pneumococcal disease.** *PLoS ONE* 2011, **6**:e23413.
3. Petrovski S, Fellay J, Shianna KV, Carpenetti N, Kumwenda J, Kamanga G, Kamwendo DD, Letvin NL, McMichael AJ, Haynes BF, et al: **Common human genetic variants and HIV-1 susceptibility: a genome-wide survey in a homogeneous African population.** *AIDS* 2011, **25**:513-518.
4. Troyer JL, Nelson GW, Lautenberger J, Chinn L, McIntosh C, Johnson RC, Sezgin E, Kessing B, Malasky M, Hendrickson SL, et al: **Genome-wide association study implicates *PARD3B* -based AIDS restriction.** *J Infect Dis* 2011, **203**:1491-1502.
5. Fellay J, Ge D, Shianna KV, Colombo S, Ledergerber B, Cirulli ET, Urban TJ, Zhang K, Gumbs CE, Smith JP, et al: **Common genetic variation and the control of HIV-1 in humans.** *PLoS Genet* 2009, **5**:e1000791.
6. Bol SM, Moerland PD, Limou S, van Remmerden Y, Coulonges C, van Manen D, Herbeck JT, Fellay J, Sieberer M, Sietzema JG, et al: **Genome-wide association study identifies single nucleotide polymorphism in *DYRK1A* associated with replication of HIV-1 in monocyte-derived macrophages.** *PLoS ONE* 2011, **6**:e17190.
7. Pelak K, Goldstein DB, Walley NM, Fellay J, Ge D, Shianna KV, Gumbs C, Gao X, Maia JM, Cronin KD, et al: **Host determinants of HIV-1 control in African Americans.** *J Infect Dis* 2010, **201**:1141-1149.
8. Le Clerc S, Limou S, Coulonges C, Carpentier W, Dina C, Taing L, Delaneau O, Labib T, Sladek R, Deveau C, et al: **Genomewide association study of a rapid progression cohort identifies new susceptibility alleles for AIDS (ANRS Genomewide Association Study 03).** *J Infect Dis 2009* , **200**:1194-1201.
9. Limou S, Le Clerc S, Coulonges C, Carpentier W, Dina C, Delaneau O, Labib T, Taing L, Sladek R, Deveau C, et al: **Genomewide association study of an AIDS-nonprogression cohort emphasizes the role played by HLA genes (ANRS Genomewide Association Study 02)**. *J Infect Dis* 2009, **199**:419-426.

Supplementary Table S5. Human genes associated with HIV-1 (HGAHs) and HIV dependency factors (HDFs) among genes previously reported as under putative selection in the Biaka.

Gene	Position	Rank	Type of Selection
EPAS1	2:46400000-46600000	52	iHS (a)
FUT8	14:64800000-65000000	83	iHS (a)
ITGA6	2:173000000-173200000	25	iHS (a)
KAT2B	3:20000000-20200000	42	iHS (a)
MAN2A1	5:109000000-109200000	4	iHS (a)
MAP2	2:209800000-210000000	67	iHS (a)
PAK1	11:76600000-76800000	40	iHS (a)
POMC	2:25200000-25400000	86	iHS (a)
SLC4A7	3:27200000-27400000	29	iHS (a)
SOD2	6:159800000-160200000	63	iHS (a)
ZAP70	2:97600000-97800000	3	iHS (a)
PIK3CG	7:106200000-106300000	5	iHS (b)
TNFSF13B	13:107700000-107800000	9	iHS (b)
ACE	17:58800000-59000000	31	XP-EHH (a)
AP2S1	19:52000000-52400000	10	XP-EHH (a)
BSG	19:400000-600000	123	XP-EHH (a)
CAMK1D	10:12400000-12600000	58	XP-EHH (a)
CDK5R1	17:27800000-28000000	80	XP-EHH (a)
CEL	9:134800000-135000000	44	XP-EHH (a)
CPN2	3:195400000-195600000	53	XP-EHH (a)
DICER1	14:94600000-94800000	41	XP-EHH (a)
EEF1A1	9:134800000-135000000	44	XP-EHH (a)
FEN1	11:61200000-61400000	52	XP-EHH (a)
FPR1	19:56800000-57000000	42	XP-EHH (a)
FPR2	19:56800000-57000000	42	XP-EHH (a)
GPX4	19:1000000-1200000	95	XP-EHH (a)
GTF2E1	3:121800000-122000000	56	XP-EHH (a)
GTF3C5	9:134800000-135000000	44	XP-EHH (a)
IRF7	11:600000-800000	77	XP-EHH (a)
ITGAM	16:31200000-31600000	75	XP-EHH (a)
ITGAX	16:31200000-31600000	75	XP-EHH (a)
LAMC3	9:132800000-133000000	54	XP-EHH (a)
LMNB1	5:126200000-126400000	112	XP-EHH (a)
LRP1	12:55800000-56000000	109	XP-EHH (a)
MME	3:156200000-156400000	61	XP-EHH (a)
NCKAP1	2:183400000-183600000	69	XP-EHH (a)
NFKB2	10:104000000-104200000	49	XP-EHH (a)
NTN1	17:9000000-9200000	88	XP-EHH (a)

Gene	Position	Rank	Type of Selection
NUP214	9:132800000-133000000	54	XP-EHH (a)
PIK3CG	7:106200000-106400000	8	XP-EHH (a)
POLR2E	19:1000000-1200000	95	XP-EHH (a)
PRKCZ	1:1800000-2200000	67	XP-EHH (a)
PSMD11	17:27800000-28000000	80	XP-EHH (a)
RAB28	4:13000000-13200000	90	XP-EHH (a)
SLC7A5	16:86400000-86600000	7	XP-EHH (a)
SPN	11:600000-800000	77	XP-EHH (a)
SPTBN4	19:45600000-45800000	59	XP-EHH (a)
STAT6	12:55800000-56000000	109	XP-EHH (a)
TATDN1	8:125400000-125600000	35	XP-EHH (a)
TIAM2	6:155000000-155200000	115	XP-EHH (a)
TMTC1	12:29800000-30000000	91	XP-EHH (a)
TNFSF13B	13:107600000-107800000	14	XP-EHH (a)
TOM1	22:34000000-34200000	66	XP-EHH (a)
WT1	11:32400000-32600000	28	XP-EHH (a)
PIK3CG	7:106100000-106400000	1	XP-EHH (b)
SLC7A5	16:86300000-86600000	10	XP-EHH (b)
TSG101	11:18500000-18800000	8	XP-EHH (b)
VPS53	17:400000-900000	3	XP-EHH (b)
CYP46A1	14:99085798-99455094	14	lnRSB
FABP1	2:88148061-88676466	23	lnRSB
MYH11	16:15438209-15913069	3	lnRSB

Methods of selection are as follows: iHS (a) regions are from a listing of the top 1% of iHS signals in the Biaka using a 200 kb sliding window and the integrated haplotype score (iHS) method. iHS (b) regions are from a listing of the top 10 iHS signals in the Biaka using a 100 kb sliding window and the iHS method. XP-EHH (a) regions are from a listing of top 1% of XP-EHH signals in the Biaka using a 200 kb sliding window and the cross-population extended haplotype homozygosity (XP-EHH) method. XP-EHH (b) regions are from a listing of the top 10 XP-EHH signals using a 200 kb sliding window moved at intervals of 100 kb. The regions under putative selection using the iHS and XP-EHH methods are from tables generated by Pickrell and colleagues [1]. lnRSB regions are from a listing of candidate regions for recent positive selection published by Lopez-Herraez and colleagues [2]. HGAHs are in boldface; the other genes are HDFs.

REFERENCES

1. Pickrell JK, Coop G, Novembre J, Kudaravalli S, Li JZ, et al. (2009) Signals of recent positive selection in a worldwide sample of human populations. Genome Res 19: 826-837.

2. Lopez Herraez D, Bauchet M, Tang K, Theunert C, Pugach I, et al. (2009) Genetic variation and recent positive selection in worldwide human populations: evidence from nearly 1 million SNPs. PLoS One 4: e7888.

Supplementary Table S6. SNPs in genes sequenced in Pygmies.

Locus	position			Consensus	Biaka (NA104-)					Mbuti (NA104-)					Trans factor (for promoter) change, amino acid (for cds) or rare codon (for synonymous substitution)
					69	70	71	72	73	92	93	94	95	96	
CCR2	Promoter (-1007 to -1)		-816	CC	CA*	.	CA*	.	A*A*	.	CA*	CA*	CA*	.	-> c-Ets-1(p54) (40 selected binding sequences)
			-636	GG	.	.	.	GA	.	GA	GA	.	.	GA	
			-555	TT	.	.	.	TA	.	.	.	.	.	.	
			-495	TT	.	TΔ*	TΔ*	TΔ*	.	TΔ*	TΔ*	.	.	TΔ*	CdxA (19 selected binding sequences)-> S8 (59 selected binding sequences), Nkx-2.5 (5 selected binding sequences belonging to the TAAT consensus)
			-488	A (n)	A (n)	A (n)	A (n)	A (n)	A (n)	A (n)	A (n)	A (n)	A (n)	A (n)	
	Exon 1	5'UTR (1 to 25)		fixed	.	.	.	.	.	.	.	.	.	.	
	Exon 2	5'UTR (467 to 485)		fixed	.	.	.	.	.	.	.	.	.	.	
		486 to 1568	675	GG	GA	.	.	.	.	.	.	GA	.	.	V -> I (Smith et al. 1997 [1], 64I delays HIV progression)
			1265	CC	CT	.	CT	.	TT	.	CT	CT	CT	.	
		3'UTR (1569 to 1653)		fixed	.	.	.	.	.	.	.	.	.	.	
CCR5	Promoter (-1001 to -1)		-697	GG	.	.	.	GT*	.	.	.	.	.	.	-> RREB-1 (11 selected binding sequences)
			-91	AA	.	.	.	.	G*G*	.	AG*	.	AG*	.	-> c-Myb (9 c-Myb binding sites in SV40 DNA + 51 selected single binding sites)
	Exon 1	5'UTR (1 to 17)		fixed	.	.	.	.	.	.	.	.	.	.	
	Exon 4	5'UTR (348 to 357)		fixed	.	.	.	.	.	.	.	.	.	.	
		348 to 1419	462	GG	.	.	.	.	GA	.	.	.	.	.	
			1353	C‡C‡	.	.	.	.	C‡T	.	.	.	.	.	CC**C** rare codon (not CC**T**)
			1361	CC	.	.	.	.	.	.	.	.	CT	.	A -> V

Locus	position			Consensus	Biaka (NA104-) 69	Biaka (NA104-) 70	Biaka (NA104-) 71	Biaka (NA104-) 72	Biaka (NA104-) 73	Mbuti (NA104-) 92	Mbuti (NA104-) 93	Mbuti (NA104-) 94	Mbuti (NA104-) 95	Mbuti (NA104-) 96	Trans factor (for promoter) change, amino acid (for cds) or rare codon (for synonymous substitution)
CUL5	Promoter (-1076 to -1)		-900	T (n)	T (n)	T (n)	T (n)	T (n)	T (n)	T (n)	T (n)	T (n)	T (n)	T (n)	
			-697	CC	.	CT	.	.	.	.	.	.	.	CT	
			-698	GG	.	.	.	.	.	.	GA	GA	.	.	
			-693	CC	.	.	.	CT	.	.	CT	.	.	.	
			-571	CC	.	.	.	.	CA	.	.	.	.	.	
			-532	TT	.	.	.	.	.	.	TA*	TA*	.	.	-> Sox-5 (23 selected binding sequences), SRY (29 selected binding sequences)
			-366	CTTACTTA	CTTAΔ*	CTTAΔ*	.	.	CTTAΔ*	.	.	.	.	.	
			-267	AA	AG*	.	.	.	.	.	.	.	.	.	-> Evi-1 (16 sites selected from random oligonucleotides)
			-137	T (n)	181 181	.	.	180 181	171 180	.	180 181	.	.	.	
	Exon 1	5'UTR (1 to 667)	314	ΔΔ	.	.	.	ΔA	.	.	.	.	ΔA	.	
		668 to 691		fixed	.	.	.	.	.	.	.	.	.	.	
	Intron 1	1 to 44		fixed	.	.	.	.	.	.	.	.	.	.	
		24373 to 24429	24415	T (n)	T (n)	T (n)	T (n)	T (n)	T (n)	T (n)	T (n)	T (n)	T (n)	T (n)	
	Exon 2	692 to 801		fixed	.	.	.	.	.	.	.	.	.	.	
	Intron 2	1 to 26	9	CC	CT	.	CT	CT	CT	TT	TT	TT	.	.	
		12325 to 12359		fixed	.	.	.	.	.	.	.	.	.	.	
	Exon 3	802 to 901	892	AA	AG	.	AG	AG	AG	GG	GG	GG	.	.	
	Intron 3	1 to 110		fixed	.	.	.	.	.	.	.	.	.	.	
		3498 to 3520		fixed	.	.	.	.	.	.	.	.	.	.	
	Exon 4	802 to 1078		fixed	.	.	.	.	.	.	.	.	.	.	
	Intron 4	1 to 45		fixed	.	.	.	.	.	.	.	.	.	.	
		2546 to 2593		fixed	.	.	.	.	.	.	.	.	.	.	
	Exon 5	1079 to 1220		fixed	.	.	.	.	.	.	.	.	.	.	
	Intron 5	1 to 29		fixed	.	.	.	.	.	.	.	.	.	.	
	Intron 14	259 to 297		fixed	.	.	.	.	.	.	.	.	.	.	
	Exon 15	2235 to 2410		fixed	.	.	.	.	.	.	.	.	.	.	
	Intron 15	1 to 542		fixed	.	.	.	.	.	.	.	.	.	.	
	Exon 16	2411 to 2572	2515	TT	TC	.	.	.	.	.	.	.	.	.	
	Intron 16	1 to 28		fixed	.	.	.	.	.	.	.	.	.	.	
		1893 to 1944		fixed	.	.	.	.	.	.	.	.	.	.	
	Exon 17	2573 to 2691		fixed	.	.	.	.	.	.	.	.	.	.	
	Intron 17	1 to 651		fixed	.	.	.	.	.	.	.	.	.	.	
	Exon 18	2692 to 2815		fixed	.	.	.	.	.	.	.	.	.	.	
	Intron 18	1 to 45	45	T (n)	T (n)	T (n)	T (n)	T (n)	T (n)	T (n)	T (n)	T (n)	T (n)	T (n)	
		5604 to 5660		fixed	.	.	.	.	.	.	.	.	.	.	
	Exon 19	2816 to 3010		fixed	.	.	.	.	.	.	.	.	.	.	
		3'UTR (3011 to 3051)		fixed	.	.	.	.	.	.	.	.	.	.	

Locus	position			Consensus	Biaka (NA104-) 69	70	71	72	73	Mbuti (NA104-) 92	93	94	95	96	Trans factor (for promoter) change, amino acid (for cds) or rare codon (for synonymous substitution)
TRIM5	Promoter (-1010 to -10)		-1000	TT	.	.	.	.	.	.	.	TC	TC	.	
			-980	AA	.	AG	.	.	.	.	.	.	.	.	
			-785	T (n)	T (n)	T (n)	T (n)	T (n)	T (n)	T (n)	T (n)	T (n)	T (n)	T (n)	
			-742	CC	.	.	.	**T*T***	.	.	.	.	.	.	GATA-3 (63 selected binding sequences) -> AML-1a (56 suggested binding sites contained in 38 selected sequences)
			-734	CC	.	.	CT*	CT*	.	.	.	.	.	.	GATA-3 (63 selected binding sequences) -> none
			-717	CC	.	.	.	.	.	.	.	.	CT	.	
			-646	TT	.	TC*	.	.	.	.	.	.	.	.	-> Sp1 (11 sites selected by TDA)
			-595	ΔΔ	Δ†*	**†*†***	.	.	.	**†*†***	**†*†***	Δ†*	**†*†***	Δ†*	Add extra Evi-1 (8 selected binding sequences)
			-588	GG	.	.	.	**A*A***	.	.	.	.	.	.	-> GATA-1 (53 selected binding sequences)
			-510	AA	.	AG	.	GG	.	.	.	.	.	.	
			-184	GG	.	GA	.	AA	GA	.	.	GA	.	GA	
			-66	GG	.	.	.	.	GC	.	.	.	.	.	
	Exon 1	5'UTR (1 to 19)	29	TT	.	TC	.	CC	TC	.	.	TC	.	TC	
			57	TT	.	TC	.	CC	.	.	.	.	.	.	
	Exon 2	278 to 463		fixed	.	.	.	.	.	.	.	.	.	.	
	Intron 6	268 to 298		fixed	.	.	.	.	.	.	.	.	.	.	
	Exon 7	1126 to 1152		fixed	.	.	.	.	.	.	.	.	.	.	
	Intron 7	1 to 63		fixed	.	.	.	.	.	.	.	.	.	.	
		214 to 260		fixed	.	.	.	.	.	.	.	.	.	.	
	Exon 8	1153 to 1363		fixed	.	.	.	.	.	.	.	.	.	.	
		3'UTR (1364 to 1767)		fixed	.	.	.	.	.	.	.	.	.	.	

Locus	position			Consensus	Biaka (NA104-) 69	70	71	72	73	Mbuti (NA104-) 92	93	94	95	96	Trans factor (for promoter) change, amino acid (for cds) or rare codon (for synonymous substitution)
TSG101	Promoter (-1006 to -1)		-861	CC	.	.	.	.	CG	.	.	.	.	.	
			-831	CC	CG*	CG*	.	.	.	.	.	.	CG*	.	-> Tst-1 (6 binding sites in the P0 promoter)
			-475	CC	CG*	CG*	.	.	.	.	.	.	CG*	.	CdxA (19 selected binding sequences) -> SRY (23 selected binding sequences), deltaEF1 (41 selected binding sequences)
			-469	ΔΔ	.	.	.	.	ΔA*	ΔA*	ΔA*	.	.	.	CdxA (19 selected binding sequences) -> none
			-422	GG	.	.	.	.	GC	GC	GC	GC	.	.	
			-396	GG	.	.	.	.	GA*	GA*	GA*	GA*	.	.	-> Ik-2 (36 selected binding sequences)
			-392	CC	.	.	.	.	.	.	.	.	CT	.	
			-291	CC	.	.	.	.	CG	CG	CG	CG	.	.	
			-152	AA	.	.	AG	.	.	AG	.	.	.	AG	
			-104	GG	GΔ	GΔ	.	.	.	.	.	.	.	.	
			-57	TT	.	.	.	.	.	TC	.	TC	.	.	
	Exon 1	5'UTR (1 to 126)		fixed	.	.	.	.	.	.	.	.	.	.	
		126 to 168		fixed	.	.	.	.	.	.	.	.	.	.	
	Intron 1	1 to 112		fixed	.	.	.	.	.	.	.	.	.	.	
		7136 to 7171		fixed	.	.	.	.	.	.	.	.	.	.	
	Exon 2	169 to 253		fixed	.	.	.	.	.	.	.	.	.	.	
	Intron 2	1 to 71		fixed	.	.	.	.	.	.	.	.	.	.	
		3310 to 3354		fixed	.	.	.	.	.	.	.	.	.	.	
	Exon 3	254 to 319		fixed	.	.	.	.	.	.	.	.	.	.	
	Intron 3	1 to 87		fixed	.	.	.	.	.	.	.	.	.	.	
		1212 to 1257		fixed	.	.	.	.	.	.	.	.	.	.	
	Exon 4	320 to 483		fixed	.	.	.	.	.	.	.	.	.	.	
	Intron 4	1 to 43	10	CC	.	.	.	.	AC	AC	AA	AA	.	.	
		4989 to 5012		fixed	.	.	.	.	.	.	.	.	.	.	
	Exon 5	484 to 607		fixed	.	.	.	.	.	.	.	.	.	.	
	Intron 5	1 to 36		fixed	.	.	.	.	.	.	.	.	.	.	
ITGAX	Promoter (-994 to -5)		-493	AA	AG*	AG*	.	.	G*G*	.	.	.	.	AG*	CdxA (18 selected synthetic and genomic binding sequences), HNF-3beta (24 binding sites [14 genomic sites from 11 genes, and 10 synthetic sites]) -> SRY (23 selected binding sequences)
			-374	AA	.	.	.	.	.	.	.	AG*	.	.	-> MZF1 (20 selected binding sequences)
			-320	CC	CA	CA	.	.	.	.	.	.	.	.	
			-144	TT	TC	TC	.	TC	.	.	.	.	.	.	
OPRM1	Promoter (-929 to -1)			fixed	.	.	.	.	.	.	.	.	.	.	
	Exon 1	5'UTR (1 to 20)	131	AA	.	AC	.	.	.	.	.	.	.	.	

Genotypes of individuals at SNPs that are identical to the NCBI dbSNP consensus are represented by period. Nucleotides are listed for individuals with genotypes that differ from the consensus. Novel SNPs are listed in boldface. SNPs that encode for a non-synonymous substitution are highlighted in yellow. SNPs that encode a rare codon are highlighted in blue and marked with "‡". SNPs that alter the transcription binding factor are marked with "*". † indicates the nucleotides TTTATT. Δ indicates a deletion.

1. Smith MW (1997) Contrasting genetic influence of *CCR2* and *CCR5* variants on HIV-1 infection and disease progression. Science 277: 959-965.

Supplementary Table S7. Oligonucleotide primers used for PCR or sequencing of genes.

Primer*	Primer Sequence	Region	Utility
CCR2			
CCR2-PF_M13F	TGTAAAACGACGGCCAGTCTTGCCAAGTCCACAGCAT	Promoter	PCR
CCR2-PR_M13R	CAGGAAACAGCTATGACTTGCATGATATGAGCACAACAG	Promoter	PCR
CCR2-PR1	TCTCTGGTAACCACTGCTCTACTG	Promoter	Sequencing
CCR2-PF1	TCTGGGTGACAGAATGAAACC	Promoter	Sequencing
CCR2EX2.1F_M13F	TGTAAAACGACGGCCAGTGGATTGAACAAGGACGCATT	Exon 2	PCR
CCR2EX2.1R_M13R	CAGGAAACAGCTATGACGGTTTTCAGGATTCCCGAGT	Exon 2	PCR
CCR2EX2.2F_M13F	TGTAAAACGACGGCCAGTGAACATTTTGGGGCTGGTC	Exon 2	PCR
CCR2EX2.2R_M13R	CAGGAAACAGCTATGACGGCACCTGCTTTACAGGTTT	Exon 2	PCR
CCR5			
CCR5-PF_M13F	TGTAAAACGACGGCCAGTGGTGCCCAAAAGGCTCTAC	Promoter	PCR
CCR5-PR_M13R	CAGGAAACAGCTATGACGGCAGGATTCTTCACTCCAG	Promoter	PCR
CCR5-PR1	TGCACTCAGTAAACATCAAACTCTT	Promoter	Sequencing
CCR5-PF1	GACTCCGGTGAACCAATTTT	Promoter	Sequencing
CCR5EX4.1F_M13F	TGTAAAACGACGGCCAGTAAGATCACTTTTTATTTATGCACAGG	Exon 4	PCR
CCR5EX4.1R_M13R	CAGGAAACAGCTATGACCAGAGTTTTTAGGATTCCCGAGT	Exon 4	PCR
CCR5EX4.2F_M13F	TGTAAAACGACGGCCAGTTAGTCATCTTGGGGCTGGTC	Exon 4	PCR
CCR5EX4.2R_M13R	CAGGAAACAGCTATGACGCCATGTGCACAACTCTGAC	Exon 4	PCR
CUL5			
CUL5-PF_M13F	TGTAAAACGACGGCCAGTATTGGAATGCTTCGGTTGAC	Promoter	PCR
CUL5-PR_M13R	CAGGAAACAGCTATGACTGCGCCCAGTAAATATGTGT	Promoter	PCR
CUL5-PR1	GGCACAGGAAATACAGGATTAAA	Promoter	Sequencing
CUL5-PF1	GCCATCCTTAACATTTGGCTA	Promoter	Sequencing
CUL5-PF2	AGTCTCGCTCTGTCGTCCA	Promoter	Sequencing
CUL5-PR2	TTCGATTTTCTGAAGGCACA	Promoter	Sequencing
CUL5-PF4	AATGACGAGGGTGTTGAACTG	Promoter	Sequencing
CUL5-PR4	CCTAATCTCCATCTACCCAAGC	Promoter	Sequencing
CUL5P-u2F-M13F	TGTAAAACGACGGCCAGTTTGGGTAGATGGAGATTAGGATAC	Promoter	Fragment analysis
CUL5P-u2R	GAGATCGCGCTATTGCACTC	Promoter	Fragment analysis
CUL5Ex1F_M13F	TGTAAAACGACGGCCAGTGCCAAGCATTCCTGAATAGAA	Exon 1	PCR

Primer*	Primer Sequence	Region	Utility
CUL5Ex1R_M13R	CAGGAAACAGCTATGACCTTTCCCAAAGAGCCGAAC	Exon 1	PCR
CUL5Ex2F_M13F	TGTAAAACGACGGCCAGTTCTAGCTGTCTCATAAAAATTACAGGA	Exon 2	PCR
CUL5Ex2R_M13R	CAGGAAACAGCTATGACGACATCACTAAAATTTCCTTTATGCTA	Exon 2	PCR
CUL5Ex3F_M13F	TGTAAAACGACGGCCAGTGATGTTTTGTTCAGTTCTTGTTTCA	Exon 3	PCR
CUL5Ex3R_M13R	CAGGAAACAGCTATGACAAATGTGTCAAAAGTCAATGCAA	Exon 3	PCR
CUL5Ex4F	CAAAGCAACTGCATTTATTTCAA	Exon 4	PCR, Sequencing
CUL5Ex4R	TCAAATGCTTCCAAATTACCC	Exon 4	PCR, Sequencing
CUL5Ex5F	TTTTGGTGTTTGTACATGATACTTTG	Exon 5	PCR, Sequencing
CUL5Ex5R	TGTTCTACAATTTCCATGAAACAGA	Exon 5	PCR, Sequencing
CUL5Ex15&16F	CCTGTATTTATTTTGAATTTTATCCAT	Eon 15 & 16	PCR, Sequencing
CUL5Ex15&16R	TTCCTGCTACATGAGAGGATACAG	Eon 15 & 16	PCR, Sequencing
CUL5Ex15&16F2	GGAAGACAGCAACCTTTTTCA	Eon 15 & 16	PCR, Sequencing
CUL5Ex15&16R2	AATTCTGCGGAGTCCTGCTA	Eon 15 & 16	PCR, Sequencing
CUL5Ex17&18F	TGATTATGTGGGAGAATTGATCTT	Exon 17 & 18	PCR, Sequencing
CUL5Ex17&18R	GCACTTAGCCCCTACATGCT	Exon 17 & 18	PCR, Sequencing
CUL5Ex17&18-2R	AAACTTCAGACATTCTGTCAACATT	Exon 17 & 18	Sequencing
CUL5Ex17&18R2	CAAAGTGCTAAAATTCAAACAAGG	Exon 17 & 18	Sequencing
CUL5Ex17&18F2	TCTTTTCTCTCAGACATTGTTGC	Exon 17 & 18	Sequencing
CUL5Ex19F	GCATAAAAAGTCATTTTTCAGTTTG	Exon 19	PCR, Sequencing
CUL5Ex19R	ACAACTTTCTGCCCAAGCAC	Exon 19	PCR, Sequencing
TRIM5			
TRIM5-PF	ACCACAGTTCAGCCTTTTG	Promoter	PCR
TRIM5-PFN	GGACCAGTTTTGCATAGTAACCA	Promoter	Sequencing
TRIM5-PR	GCCTGATCTGCACAAAGGA	Promoter	PCR, Sequencing
TRIM5-PR1	TGCAGTAGATTTCTGGTCTGGA	Promoter	Sequencing
TRIM5-PF1	CGGCCCTCTCCAGATAAAT	Promoter	Sequencing
TRIM5-PR3	AGAAATACCACATTATTTCTTTCAACT	Promoter	Sequencing
TRIM5-RD-F-M13F	TGTAAAACGACGGCCAGTATGGCTTCTGGAATCCTGGT	Exon 2 (ring domain)	PCR
TRIM5-RD-R-M13R	CAGGAAACAGCTATGACTGTTGGCTACATGCCGATTA	Exon 2 (ring domain)	PCR
TRIM5-SD1-F-M13F	TGTAAAACGACGGCCAGTCAGTGCTGACTCCTTTGTTTG	Exon 7 (SPRY domain)	PCR

Primer*	Primer Sequence	Region	Utility
TRIM5-SD1-R-M13R	CAGGAAACAGCTATGACTTAAAACATGAGCCTAATAGAGAACA	Exon 7 (SPRY domain)	PCR
TRIM5-SD2-F-M13F	TGTAAAACGACGGCCAGTTTTATCAAGATTTCTCTCATATCACAA	Exon 8 (SPRY domain)	PCR
TRIM5-SD2-R-M13R	CAGGAAACAGCTATGACACCTGGACAAGAGGTGCTGT	Exon 8 (SPRY domain)	PCR
TSG101			
TSG101-PF_M13F	TGTAAAACGACGGCCAGTAGCCTTTATTTGTGCGTGTT	Promoter	PCR
TSG101-PR_M13R	CAGGAAACAGCTATGACGAGCTGGCTCTCCGACAC	Promoter	PCR
TSG101-PF1	TGGCTTCTTGTGAAACAAAGG	Promoter	Sequencing
TSG101-PR1	GATGCCCAGTGGTCCTCTAA	Promoter	Sequencing
TSG101-PF2	CCAGGCCCCTCTCAATCC	Promoter	Sequencing
TSG101-PR2	TCACCGTCAGTTTTGTAAGCA	Promoter	Sequencing
TSG101EX1F_M13F	TGTAAAACGACGGCCAGTTTGTGTGGGACGGTCTGG	Exon 1	PCR
TSG101EX1R_M13R	CAGGAAACAGCTATGACGAGGTCGCTAAGGACTGCAC	Exon 1	PCR
TSG101EX2F_M13F	TGTAAAACGACGGCCAGTCCAGCTGTAAAATATATGCATTGG	Exon 2	PCR
TSG101EX2R_M13R	CAGGAAACAGCTATGACTCCACAAACCTCAAATGGAA	Exon 2	PCR
TSG101EX3F_M13F	TGTAAAACGACGGCCAGTTTTCTGAAAGAATAACTTATGGGAGT	Exon 3	PCR
TSG101EX3R_M13R	CAGGAAACAGCTATGACGCATCAAAGCCCTGAGAAAG	Exon 3	PCR
TSG101EX4F_M13F	TGTAAAACGACGGCCAGTTGTTCCTTTAACCATTCTTCAATTT	Exon 4	PCR
TSG101EX4R_M13R	CAGGAAACAGCTATGACAAGCAGATGCTAGTGAGCAAAA	Exon 4	PCR
TSG101EX5F_M13F	TGTAAAACGACGGCCAGTTGAAAAGCAACTCTGTAGCTGA	Exon 5	PCR
TSG101EX5R_M13R	CAGGAAACAGCTATGACCAAAGGTTTCTGTTCTCTTTTGTTT	Exon 5	PCR
ITGAX			
ITGAX-PF_M13F	TGTAAAACGACGGCCAGTATTCAGGTGCAGTGGTGCAA	Promoter	PCR
ITGAX-PR_M13R	CAGGAAACAGCTATGACGGAAGAGCTGGACCAAGGTA	Promoter	PCR
ITGAX-PR1	TCTGCTAGGTGGGGAAGAAA	Promoter	Sequencing
ITGAX-PF1	CATCTGCCTCTCCACTGACC	Promoter	Sequencing
ITGAX-PFN	GTGCAGTGGTGCAATCCTAT	Promoter	Sequencing
ITGAX-PRN	GCTGGACCAAGGTACTGAGG	Promoter	Sequencing
OPRM1			
OPRM1-PF_M13F	TGTAAAACGACGGCCAGTTTTTCAAGACCAACTGAGGACA	Promoter	PCR
OPRM1-PR_M13R	CAGGAAACAGCTATGACTATAGCCCCCTCCCACCTTA	Promoter	PCR

Primer*	Primer Sequence	Region	Utility
OPRM1-PR1	GGGAACAACAGCGATCCTT	Promoter	Sequencing
OPRM1-PF1	TGAGGAACAGGTTTTCTGCAC	Promoter	Sequencing
All genes			
M13F	TGTAAAACGACGGCCAGT		Sequencing
M13R	CAGGAAACAGCTATGAC		Sequencing

*Primers with names that include "_M13F" or "_M13R" have the M13 foward sequence or M13 reverse sequence, respectively, as part of their primer sequence at the 5' end. PCR amplicons generated by these primers could be sequenced using just the M13F or M13R primers listed at the bottom of the table under the category "all genes".

Supplementary Figure S1. Geographic distribution of chimpanzee subspecies and phylogenetic relationship of strains of immunodeficiency viruses. Description: (A) Map of west and central Africa showing the geographic ranges of subspecies of the common chimpanzee (*Pan troglodytes troglodytes*), based on Keele et al. 2006 [1]. HIV-1 derives from strains of SIV carried only by *P. t. troglodytes*, while *P. t. schweinfurthii* carries SIV strains that are unrelated to HIV-1. SIV is not present in the other chimpanzee subspecies. (B) Phylogenetic relationship among immunodeficiency viruses, based on Sharp & Hahn 2010 [2]. Strains of SIV from chimpanzees (SIVcpz) are carried by the subspecies *P. t. troglodytes* (red) and *P. t. schweinfurthii* (grey); gorilla (*Gorilla gorilla*) strains are labeled SIVgor (blue); human HIV-1 strains are in black. A red X marks the four branches on which independent cross-species transmission to humans occurred; the two blue crosses indicate alternative possible branches on which a chimpanzee-to-gorilla transmission occurred. The HIV-1 strains fall into four groups (M, N and O and RBF168).

REFERENCES

1. Keele BF, Van Heuverswyn F, Li Y, Bailes E, Takehisa J, Santiago ML, Bibollet-Ruche F, Chen Y, Wain LV, Liegeois F, et al: **Chimpanzee reservoirs of pandemic and nonpandemic HIV-1.** *Science* 2006, **313:**523-526.
2. Sharp PM, Hahn BH: **The evolution of HIV-1 and the origin of AIDS.** *Philos Trans R Soc Lond B Biol Sci* 2010, **365:**2487-2494.

Supplementary Figure S1a. Geographic distribution of chimpanzee subspecies.

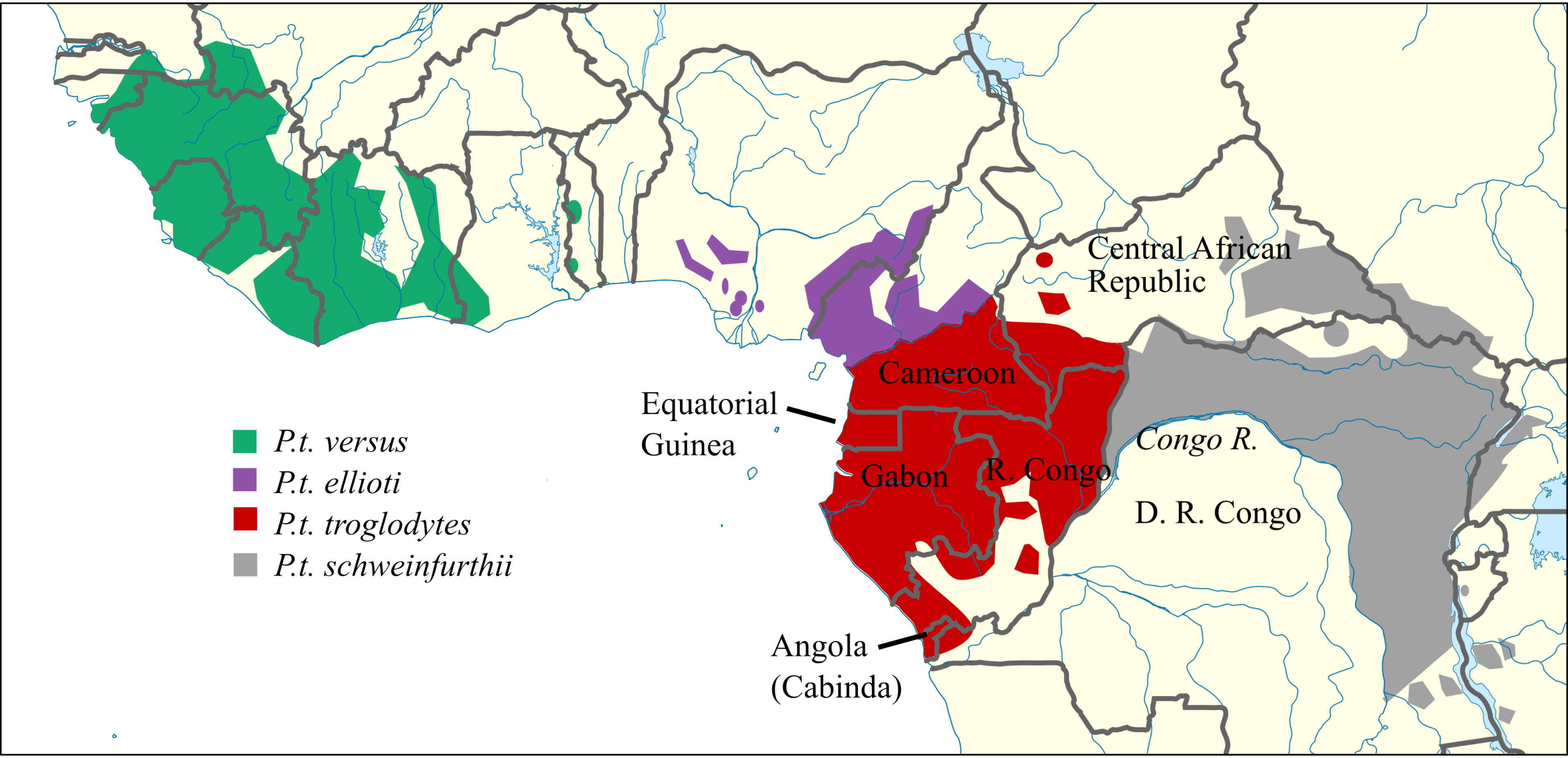

Supplementary Figure S1b. Phylogenetic relationship of strains of immunodeficiency viruses.

Virus strain indicated by color:

SIV from *Pan troglodytes troglodytes*

SIV from *Pan troglodytes schweinfurthii*

SIV from *Gorilla gorilla*

HIV-1 in *Homo sapiens*

X potential cross-species transmission

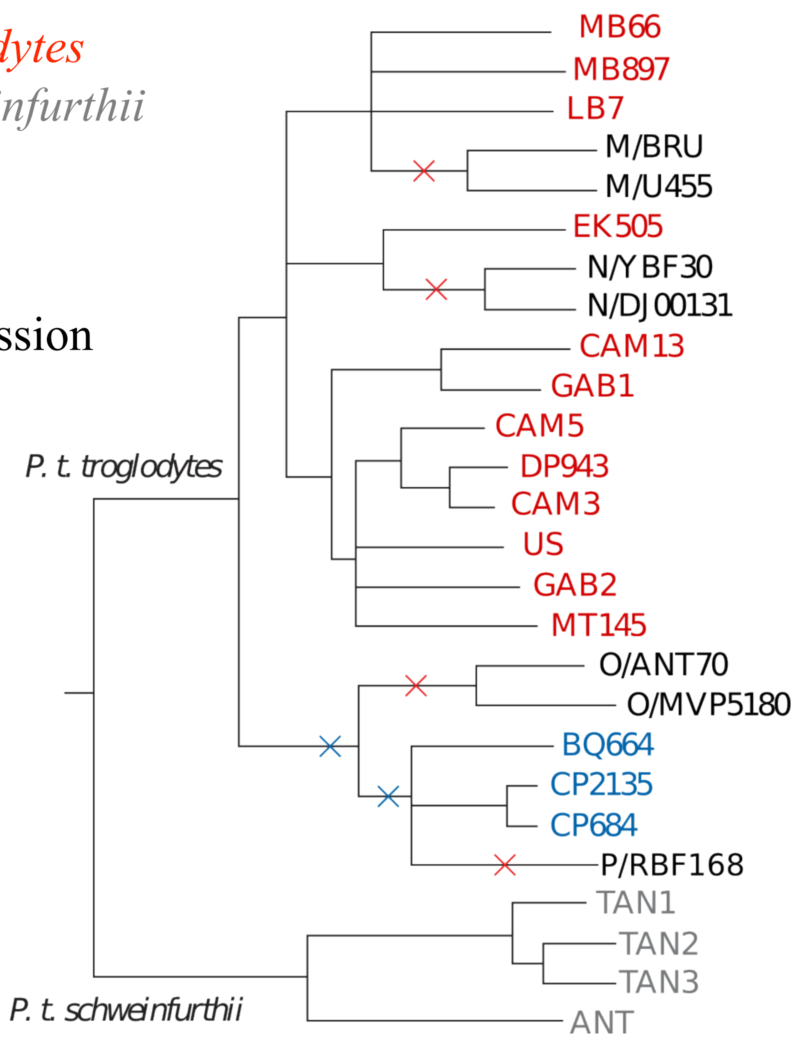

Supplementary Figure S2. Identification of types of selection based on genomic patterns. (Top panel:) The *x*-axis represents simulated SNPs across a chromosome. The *y*-axis in the first two graphs shows scores based on levels of heterozygosity ($\hat{H}$) around SNPs for each of two populations being compared, where reduced heterozygosity is shown as a higher score (λ value). The *y*-axis in the third graph shows scores calculated for variance in F_{ST} (S^2F_{ST}), with higher variance in F_{ST} indicated by a higher score. (Bottom panel:) The type of selection was inferred as follows [1]: low heterozygosity (high peak) in one population and high variance of F_{ST} indicate a signature of "new" selection (post-dating the split between the two populations) in the population with low heterozygosity. Low heterozygosity (high peaks) in both populations, along with high variance in F_{ST}, constitute a signature of "new" selection in both populations. If heterozygosity was low (high peaks) in both populations, but variance in F_{ST} was low, then a signature of "old" selection (pre-dating the split between the two populations) was inferred. The illustration is derived from reference [1].

REFERENCE

1. Oleksyk TK, Zhao K, Vega FMDL, Gilbert DA, O'Brien SJ, Smith MW: **Identifying selected regions from heterozygosity and divergence using a light-coverage genomic dataset from two human populations.** *PloS one* 2008, **3:**e1712.

[Supplementary Figure S2]

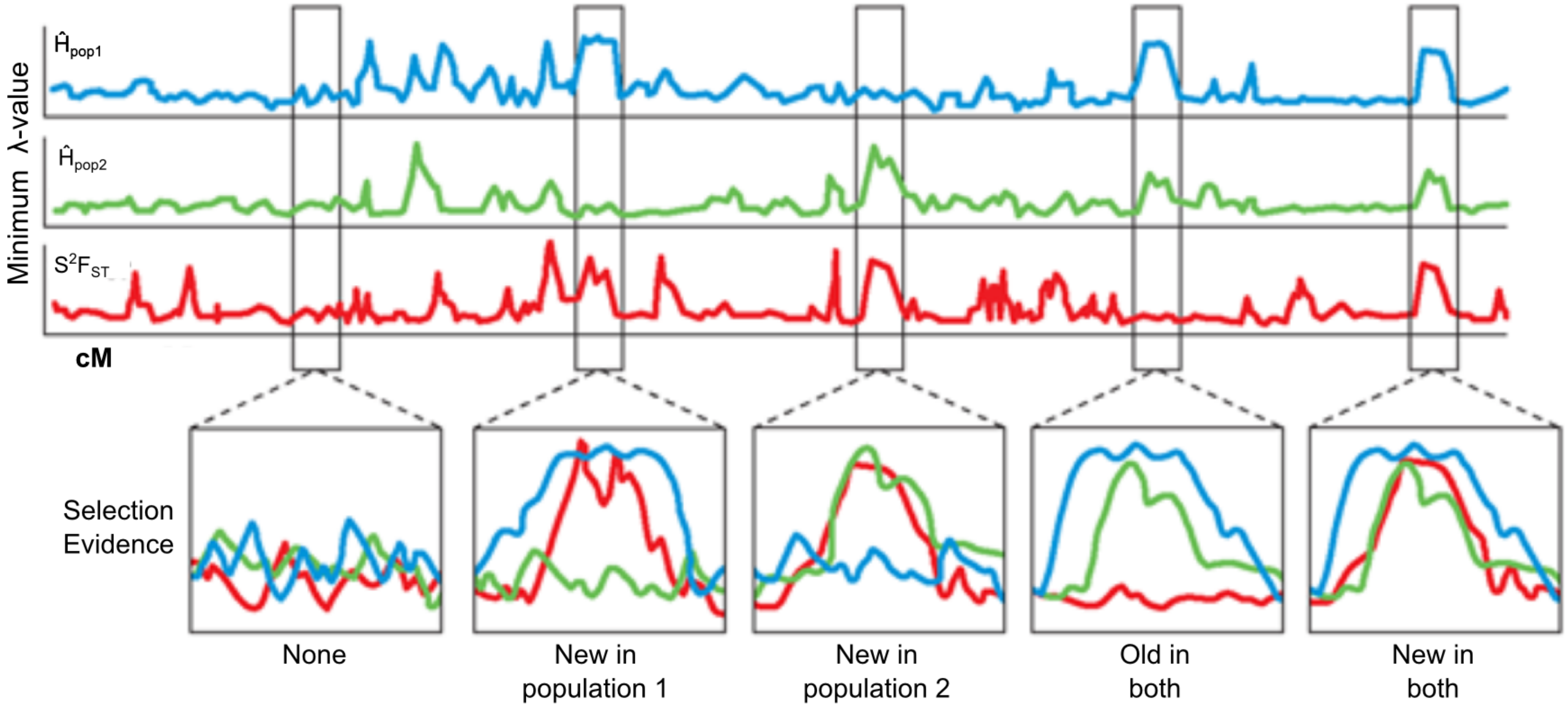

Supplementary Figure S3. Length in kb of genomic regions under putative selection, with number of SNPs and genes within each region.

Description: The *x*-axis indicates the length in kb of regions displaying signatures of new or old selection in Biaka-Mbuti comparisons. The *y*-axis shows the number of SNPs present within the regions showing signatures of selection. The values plotted within parentheses indicate the number of genes present within each genomic region that displayed a signature of selection. The regions under putative selection that include human genes associated with HIV-1 are labeled with the name of the gene. The region under putative selection around *CUL5* is one of the longest present in the genome, which may signal a stronger degree or a more recent occurrence of selection [1].

REFERENCE

1. Oleksyk TK, Smith MW, O'Brien SJ: **Genome-wide scans for footprints of natural selection.** *Philosophical transactions of the Royal Society of London Series B, Biological sciences* 2010, **365:**185-205.

[Supplementary Figure S3]

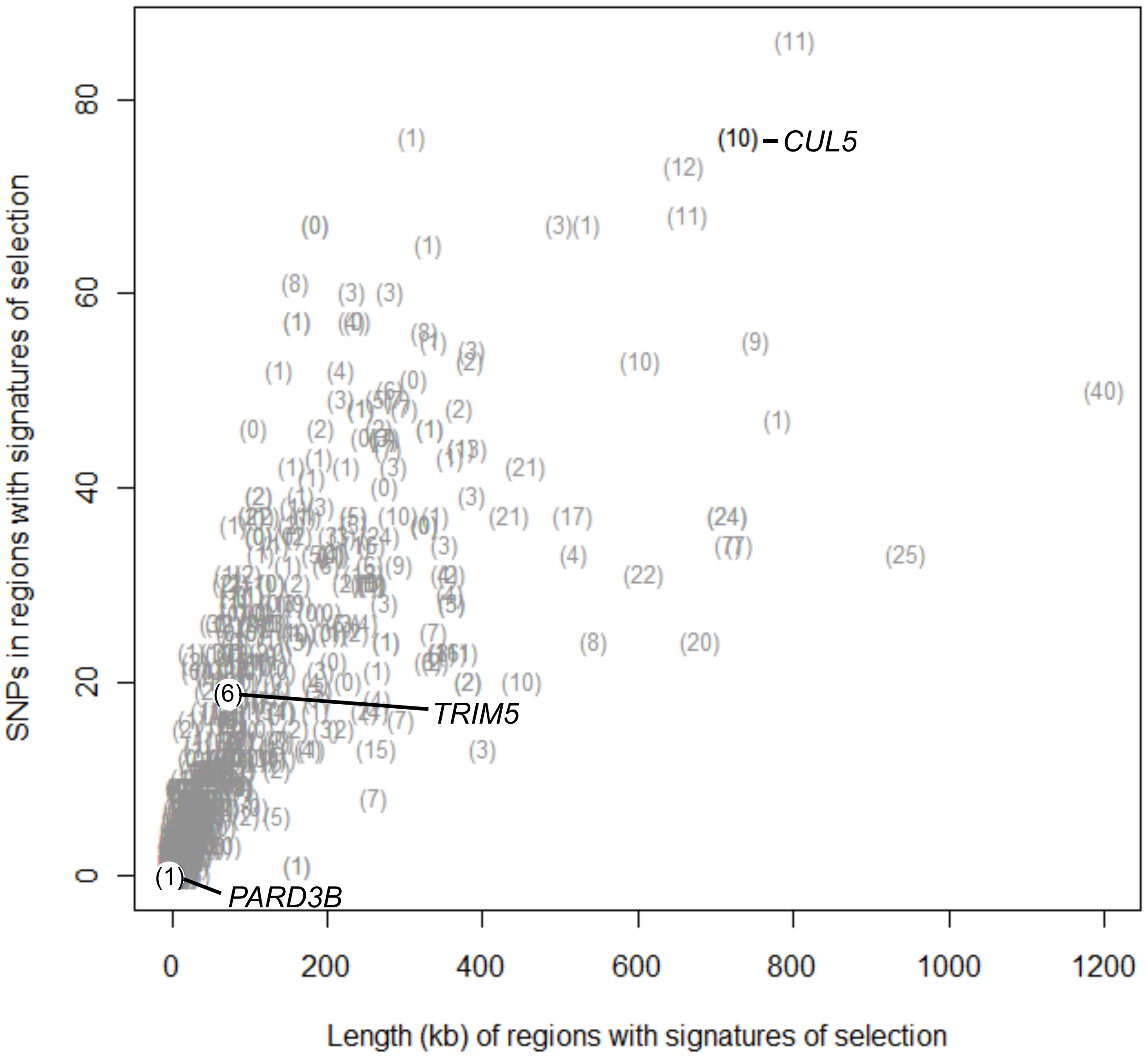

Supplementary Figure S4. List of all HGAHs and HDFs found in regions with signatures of selection for all pairwise comparisons.

Description: The lists of HGAHs and HDFs, including the NIH listing of host genes that may interact with HIV-1, were matched against each of the genomic regions displaying signatures of selection, as determined by applying the method of Oleksyk (2008) [1]. Each pair of adjacent similarly shaded populations listed along the top was compared. HGAHs and HDFs are listed in order of strength of selection; yellow indicates a greater signal of selection; red indicates a lesser signal of selection. Genes in regions with signatures of old selection are listed in cells without black borders and are listed under the columns of both populations involved. Genes in regions with signatures of new selection are listed in cells with black borders: A single cell with color indicates new selection in that population; a pair of cells with color indicates new selection in both populations. Genes listed in bold are HGAHs (and may also be HDFs); otherwise, the genes are HDFs but not HGAHs.

REFERENCE

1. Oleksyk TK, Zhao K, Vega FMDL, Gilbert DA, O'Brien SJ, Smith MW: **Identifying selected regions from heterozygosity and divergence using a light-coverage genomic dataset from two human populations.** *PloS one* 2008, **3:**e1712.

Supplementary Figure S4. List of all HGAHs and HDFs found in regions with signatures of selection for all pairwise comparisons.

Region under putative selection	Biaka	Mbuti	Biaka	Bantu	Mbuti	Bantu	Biaka	Yoruba	Mbuti	Yoruba	Biaka	Mandenka	Mbuti	Mandenka	Bantu	Mandenka	Bantu	Yoruba	Mandenka	Yoruba	Gene Name
6:32874832-32906709																					*HLA-DOB*
11:107122467-107851313																					*ATM*
11:107122467-107851313																					***CUL5***
11:72131498-72483251																					*ATG16L2*
12:93915516-93996220																					*FGD6*
6:28541114-28808331																					*GPX5*
6:28541114-28808331																					*GPX6*
13:48904591-49229993																					*KPNA3*
4:123320561-123721113																					*IL2*
6:32904663-33063954																					*HLA-DMA*
6:32904663-33063954																					*HLA-DMB*
6:32904663-33063954																					*PSMB8*
6:32904663-33063954																					*PSMB9*
6:32904663-33063954																					*TAP1*
16:31273517-31535883																					*ITGAX*
2:179590507-179848028																					*SESTD1*
10:76140605-76493170																					*MYST4*
2:100249892-100387056																					*CHST10*
12:69270162-69270286																					*PTPRB*
6:33739674-33739674																					*ITPR3*
11:124907656-125122820																					*CHEK1*
11:124907656-125122820																					*STT3A*
17:40030438-40411105																					*GFAP*
7:31754514-31835138																					*PDE1C*
7:31754514-31835138																					*PDE1C*
11:5712786-5712786																					*TRIM22*
11:5712786-5712786																					***TRIM5***
2:225501079-225511799																					*DOCK10*
2:27113298-27201533																					*AGBL5*
2:27113298-27201533																					*CGREF1*
17:5292515-5307671																					*DHX33*
18:22941322-23017276																					*CHST9*
7:31754514-31835138																					*PDE1C*

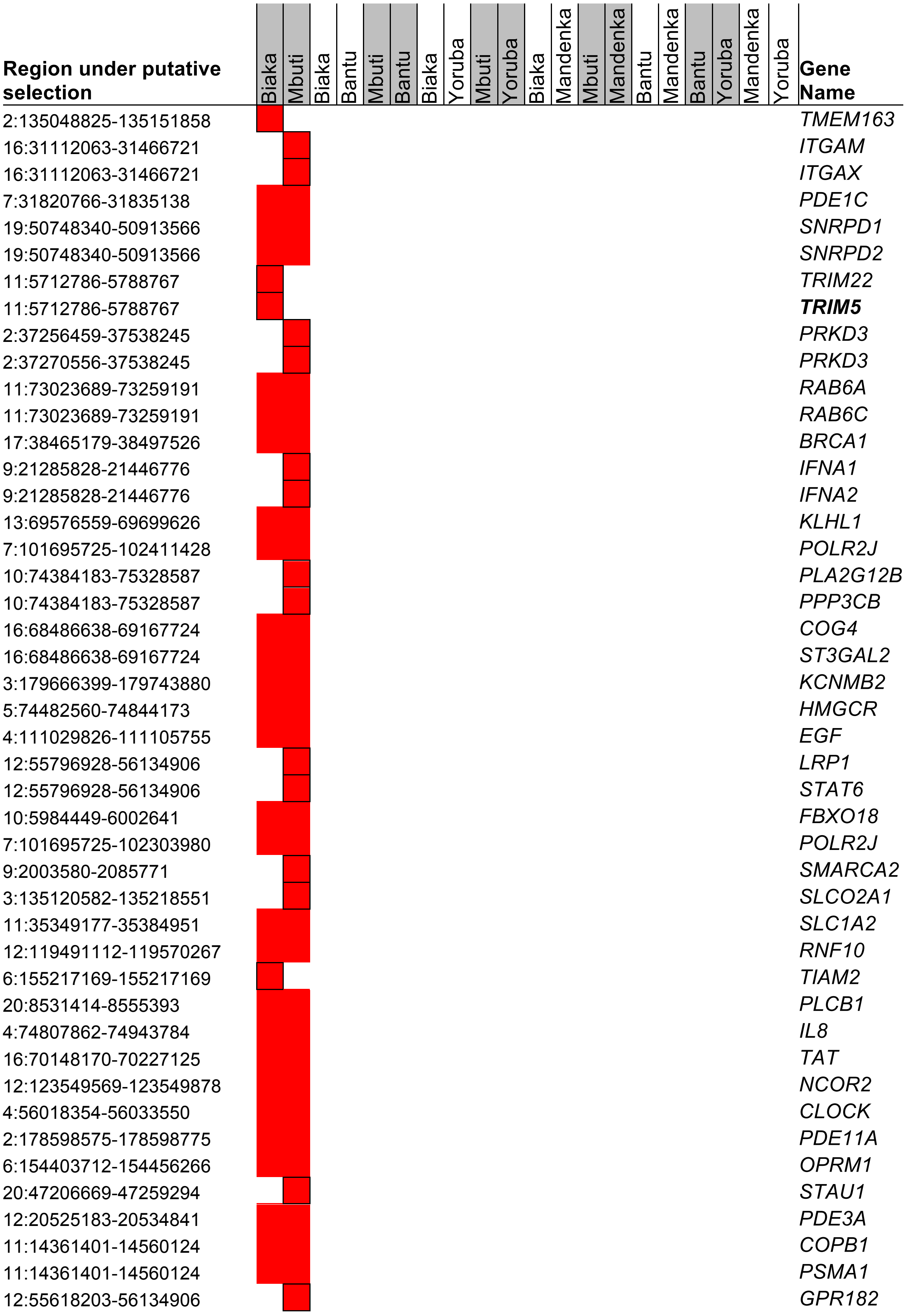

Region under putative selection
Biaka
Mbuti
Biaka
Bantu
Mbuti
Bantu
Biaka
Yoruba
Mbuti
Yoruba
Biaka
Mandenka
Mbuti
Mandenka
Bantu
Mandenka
Bantu
Yoruba
Mandenka
Yoruba
Gene Name
2:135048825-135151858
16:31112063-31466721
16:31112063-31466721
7:31820766-31835138
19:50748340-50913566
19:50748340-50913566
11:5712786-5788767
11:5712786-5788767
2:37256459-37538245
2:37270556-37538245
11:73023689-73259191
11:73023689-73259191
17:38465179-38497526
9:21285828-21446776
9:21285828-21446776
13:69576559-69699626
7:101695725-102411428
10:74384183-75328587
10:74384183-75328587
16:68486638-69167724
16:68486638-69167724
3:179666399-179743880
5:74482560-74844173
4:111029826-111105755
12:55796928-56134906
12:55796928-56134906
10:5984449-6002641
7:101695725-102303980
9:2003580-2085771
3:135120582-135218551
11:35349177-35384951
12:119491112-119570267
6:155217169-155217169
20:8531414-8555393
4:74807862-74943784
16:70148170-70227125
12:123549569-123549878
4:56018354-56033550
2:178598575-178598775
6:154403712-154456266
20:47206669-47259294
12:20525183-20534841
11:14361401-14560124
11:14361401-14560124
12:55618203-56134906
TMEM163
ITGAM
ITGAX
PDE1C
SNRPD1
SNRPD2
TRIM22
TRIM5
PRKD3
PRKD3
RAB6A
RAB6C
BRCA1
IFNA1
IFNA2
KLHL1
POLR2J
PLA2G12B
PPP3CB
COG4
ST3GAL2
KCNMB2
HMGCR
EGF
LRP1
STAT6
FBXO18
POLR2J
SMARCA2
SLCO2A1
SLC1A2
RNF10
TIAM2
PLCB1
IL8
TAT
NCOR2
CLOCK
PDE11A
OPRM1
STAU1
PDE3A
COPB1
PSMA1
GPR182

Region under putative selection	Biaka	Mbuti	Biaka	Bantu	Mbuti	Bantu	Biaka	Yoruba	Mbuti	Yoruba	Biaka	Mandenka	Mbuti	Mandenka	Bantu	Mandenka	Bantu	Yoruba	Mandenka	Yoruba	Gene Name
12:55618203-56134906																					*LRP1*
12:55618203-56134906																					*STAT6*
3:46904550-46953927																					*PTHR1*
7:101695725-102411428																					*POLR2J*
11:76670312-77272002																					*CLNS1A*
11:76670312-77272002																					*PAK1*
10:78894205-78905202																					*KCNMA1*
6:26298726-26375779																					*HIST1H2A*
6:26298726-26375779																					*HIST1H2A*
6:26298726-26375779																					*HIST1H2B*
6:26298726-26375779																					*HIST1H2B*
6:26298726-26375779																					*HIST1H2B*
6:26298726-26375779																					*HIST1H3A*
6:26298726-26375779																					*HIST1H3B*
6:26298726-26375779																					*HIST1H3C*
6:26298726-26375779																					*HIST1H3D*
6:26298726-26375779																					*HIST1H3E*
6:26298726-26375779																					*HIST1H3F*
6:26298726-26375779																					*HIST1H3G*
6:26298726-26375779																					*HIST1H3H*
6:26298726-26375779																					*HIST1H3I*
6:26298726-26375779																					*HIST1H3J*
6:26298726-26375779																					*HIST1H4A*
6:26298726-26375779																					*HIST1H4B*
6:26298726-26375779																					*HIST1H4C*
6:26298726-26375779																					*HIST1H4D*
6:26298726-26375779																					*HIST1H4E*
6:26298726-26375779																					*HIST1H4F*
6:26298726-26375779																					*HIST1H4G*
6:26298726-26375779																					*HIST1H4H*
6:26298726-26375779																					*HIST1H4I*
6:26298726-26375779																					*HIST1H4J*
6:26298726-26375779																					*HIST1H4K*
6:26298726-26375779																					*HIST1H4L*
6:26298726-26375779																					*HIST2H4A*
6:26298726-26375779																					*HIST4H4*
5:170173072-170173072																					*GABRP*
12:8648058-8905984																					*AICDA*
11:227087-232112																					*PSMD13*
12:84127662-84147479																					*LRRIQ1*
16:46053294-46215694																					*PHKB*
16:46053294-46215694																					*PHKB*
10:33228108-33282407																					*ITGB1*
17:27662317-27799874																					*PSMD11*
5:59277491-59463499																					*PDE4D*
2:205683626-205683626																					***PARD3B***

Region under putative selection	Biaka	Mbuti	Biaka	Bantu	Mbuti	Bantu	Biaka	Yoruba	Mbuti	Yoruba	Biaka	Mandenka	Mbuti	Mandenka	Bantu	Mandenka	Bantu	Yoruba	Mandenka	Yoruba	Gene Name
12:119491112-119570267																					*RNF10*
22:27886739-28177722																					*AP1B1*
12:8648058-8904596																					*AICDA*
16:31273517-34637302																					*ITGAX*
16:31273517-34637302																					*ZNF720*
10:96023756-96427689																					*PLCE1*
3:128745447-128922832																					*ABTB1*
19:43477158-43811334																					*PSMD8*
5:167783746-167985714																					*PANK3*
14:73236430-73483173																					*DNAL1*
21:46610952-46841625																					*PCNT*
2:9611326-9733217																					*YWHAQ*
17:24859695-25068227																					*TAOK1*
5:151032339-151270918																					*ATOX1*
16:69996550-70205800																					*CHST4*
16:69996550-70205800																					*TAT*
12:93905833-93995461																					*FGD6*
11:5810765-5922991																					***TRIM5***
6:32249861-32273561																					*RNF5*
20:8247380-8273418																					*PLCB1*
22:33994327-34154824																					*TOM1*
2:70558910-70564691																					*TGFA*
11:73140406-73259556																					*RAB6A*
11:73140406-73259556																					*RAB6C*
4:74843755-74969011																					*CXCL1*
2:178576047-178708537																					*PDE11A*
1:92717582-93125257																					*RPL5*
14:64992878-65015804																					*FUT8*
3:128745447-128922832																					*ABTB1*
16:23435624-23619949																					*ERN2*
16:23435624-23619949																					*PLK1*
10:6530227-6544132																					*PRKCQ*
14:23800302-23840209																					*TGM1*
5:118551008-118598071																					*DMXL1*
17:70822168-71019461																					*GRB2*
11:65943458-66316599																					*SPTBN2*
12:26670546-26670546																					*ITPR2*
5:32144879-32171356																					*GOLPH3*
19:48635339-48705100																					*ETHE1*
19:14513278-14538312																					*GPSN2*
19:14513278-14538312																					*NDUFB7*
1:100948239-100988583																					*VCAM1*
1:107915388-107957883																					*VAV3*
13:107717404-107741035																					*TNFSF13B*
10:5984449-6000636																					*FBXO18*
12:38637192-38779683																					*SLC2A13*

Region under putative selection	Biaka	Mbuti	Biaka	Bantu	Mbuti	Bantu	Biaka	Yoruba	Mbuti	Yoruba	Biaka	Mandenka	Mbuti	Mandenka	Bantu	Mandenka	Bantu	Yoruba	Mandenka	Yoruba	Gene Name
8:11409494-11409494			■	■																	*BLK*
5:59269104-59308563			■																		*PDE4D*
11:11556970-11587617			■	■																	*GALNTL4*
8:134325741-134332892			■	■																	*NDRG1*
6:12194812-12194812			■	■																	*HIVEP1*
17:544990-565912			■	■																	*VPS53*
15:53952815-54096833			■	■																	*NEDD4*
7:31826228-31859481			■	■																	*PDE1C*
10:12478789-12486578			■	■																	*CAMK1D*
12:119324905-119553584			■	■																	*RNF10*
5:179857956-179876561			■	■																	*CCR4*
3:42766127-42841126			■	■																	*CCBP2*
11:14441221-14468636			■	■																	*COPB1*
7:102347004-102794835			■	■																	*PSMC2*
7:7647656-7648700			■	■																	*RPA3*
19:2049015-2060019			■																		*AP3D1*
22:30678239-30701492			■	■																	*YWHAH*
6:155054005-155224256			■																		*TIAM2*
11:226871-233268			■	■																	*PSMD13*
11:226871-233268			■	■																	*PSMD13*
2:37164843-37207653			■	■																	*EIF2AK2*
12:15706025-15707557				■																	*EPS8*
18:65551119-65551119			■	■																	*DOK6*
6:154383069-154420845			■	■																	*OPRM1*
15:56783117-56784541			■	■																	*ADAM10*
1:180377811-180826133						■															*RNASEL*
10:74363276-76043910						■															*AP3M1*
10:74363276-76043910						■															*PLA2G12B*
10:74363276-76043910						■															*PLAU*
10:74363276-76043910						■															*PPP3CB*
10:74363276-76043910						■															*VCL*
7:100639893-100857977						■															*PLOD3*
2:197241610-197585174						■															*GTF3C3*
1:184890093-185136886						■															*PLA2G4A*
1:184890093-185136886						■															*PTGS2*
21:46655271-46676181						■															*PCNT*
16:34132640-45212171					■	■															*SHCBP1*
5:109016922-109136881						■															*MAN2A1*
15:63548169-64159966					■	■															*RAB11A*
15:63548169-64159966					■	■															*SLC24A1*
18:17043299-17610456						■															*SNRPD1*
15:40769632-41848175						■															*MAP1A*
15:40769632-41848175						■															*PDIA3*
15:40769632-41848175						■															*TGM5*
15:40769632-41848175						■															*TGM7*
11:47301676-47655974						■															*MADD*

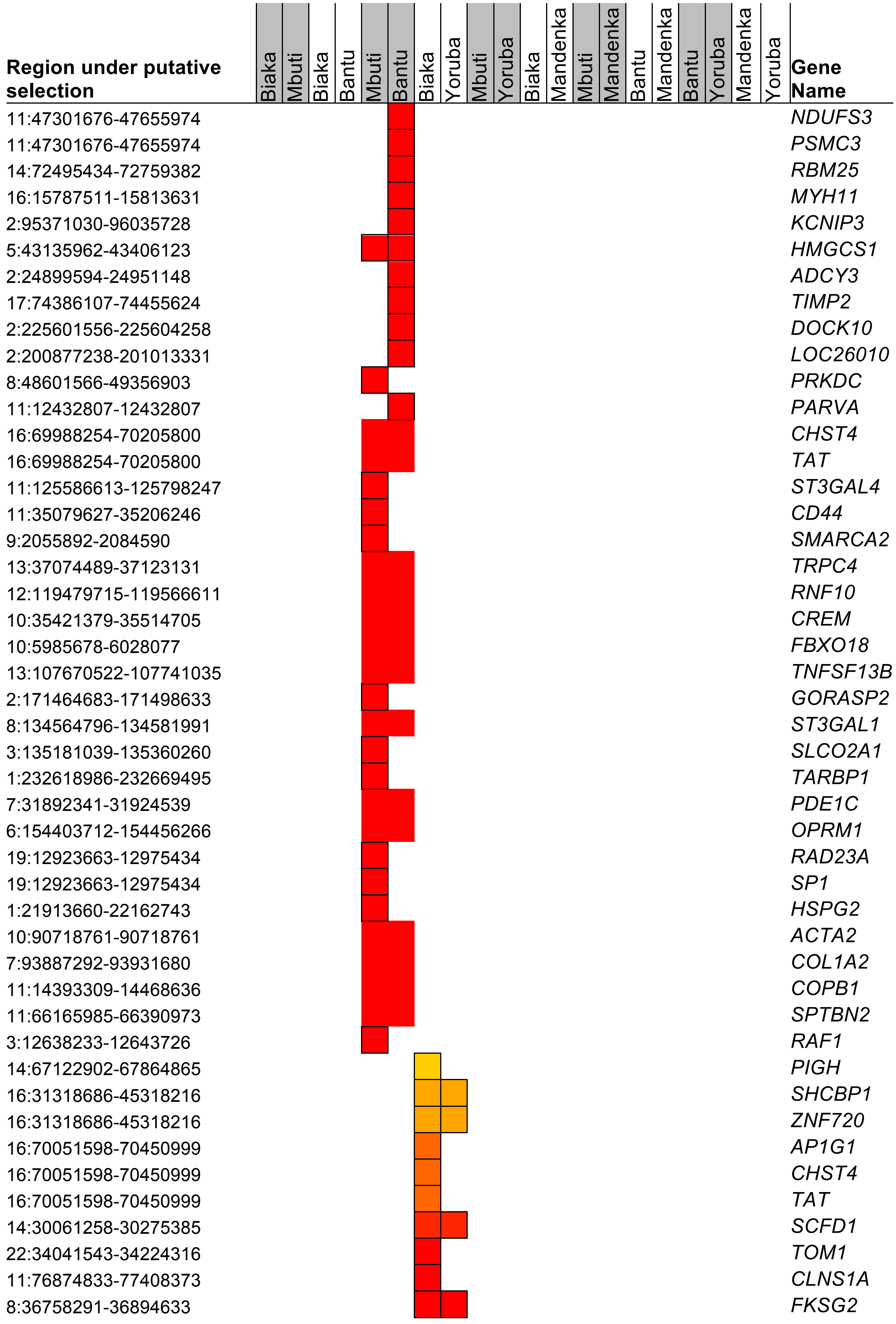
Region under putative selection
Biaka
Mbuti
Biaka
Bantu
Mbuti
Bantu
Biaka
Yoruba
Mbuti
Yoruba
Biaka
Mandenka
Mbuti
Mandenka
Bantu
Mandenka
Bantu
Yoruba
Mandenka
Yoruba
Gene Name
11:47301676-47655974 NDUFS3
11:47301676-47655974 PSMC3
14:72495434-72759382 RBM25
16:15787511-15813631 MYH11
2:95371030-96035728 KCNIP3
5:43135962-43406123 HMGCS1
2:24899594-24951148 ADCY3
17:74386107-74455624 TIMP2
2:225601556-225604258 DOCK10
2:200877238-201013331 LOC26010
8:48601566-49356903 PRKDC
11:12432807-12432807 PARVA
16:69988254-70205800 CHST4
16:69988254-70205800 TAT
11:125586613-125798247 ST3GAL4
11:35079627-35206246 CD44
9:2055892-2084590 SMARCA2
13:37074489-37123131 TRPC4
12:119479715-119566611 RNF10
10:35421379-35514705 CREM
10:5985678-6028077 FBXO18
13:107670522-107741035 TNFSF13B
2:171464683-171498633 GORASP2
8:134564796-134581991 ST3GAL1
3:135181039-135360260 SLCO2A1
1:232618986-232669495 TARBP1
7:31892341-31924539 PDE1C
6:154403712-154456266 OPRM1
19:12923663-12975434 RAD23A
19:12923663-12975434 SP1
1:21913660-22162743 HSPG2
10:90718761-90718761 ACTA2
7:93887292-93931680 COL1A2
11:14393309-14468636 COPB1
11:66165985-66390973 SPTBN2
3:12638233-12643726 RAF1
14:67122902-67864865 PIGH
16:31318686-45318216 SHCBP1
16:31318686-45318216 ZNF720
16:70051598-70450999 AP1G1
16:70051598-70450999 CHST4
16:70051598-70450999 TAT
14:30061258-30275385 SCFD1
22:34041543-34224316 TOM1
11:76874833-77408373 CLNS1A
8:36758291-36894633 FKSG2

Region under putative selection	Biaka	Mbuti	Biaka	Bantu	Mbuti	Bantu	Biaka	Yoruba	Mbuti	Yoruba	Biaka	Mandenka	Mbuti	Mandenka	Bantu	Mandenka	Bantu	Yoruba	Mandenka	Yoruba	Gene Name
7:31774864-31859481							■														*PDE1C*
12:93928789-93995461							■	■													*FGD6*
13:107634383-107767871							■	■													*TNFSF13B*
19:10613968-10755922							■	■													*DNM2*
19:10613968-10755922							■	■													*ILF3*
10:32708723-33305561							■	■													*ITGB1*
15:27693153-27833209							■	■													*TJP1*
1:153675259-154530564							■														*DAP3*
1:153675259-154530564							■														*LMNA*
1:153675259-154530564							■														*MAPBPIP*
1:153675259-154530564							■														*UBQLN4*
2:95035474-96047105								■													*KCNIP3*
2:183086558-183234573							■														*PDE1A*
10:90737149-90792904							■														*ACTA2*
10:90737149-90792904							■														*FAS*
20:31912990-32247488							■														*EIF2S2*
5:74654888-75024125							■														*HMGCR*
1:20281961-20317060							■														*PLA2G2D*
1:20281961-20317060							■														*PLA2G5*
2:24617313-24982223								■													*ADCY3*
2:24617313-24982223								■													*NCOA1*
1:181456056-181538918							■														*LAMC2*
20:29324345-29817803							■	■													*BCL2L1*
20:29324345-29817803							■	■													*ID1*
17:26151077-26676582							■														*EVI2A*
17:26151077-26676582							■														*EVI2A*
17:26151077-26676582							■														*EVI2A*
6:96104083-96142637							■														*MANEA*
9:74250072-74378110							■	■													*TMC1*
11:9885582-10118803								■													*SBF2*
1:23587252-23709352							■	■													*TCEA3*
21:33709182-33848130							■	■													*IFNGR2*
8:22842941-22942763							■	■													*TNFRSF1*
10:5984449-6002641							■	■													*FBXO18*
8:19857956-19869148							■														*LPL*
11:11518582-11608164							■	■													*GALNTL4*
1:66146592-66161685								■													*PDE4B*
20:39028374-39154787							■	■													*TOP1*
6:33205592-33656372								■													*B3GALT4*
6:33205592-33656372								■													*PHF1*
18:22940363-23004144							■														*CHST9*
2:178502285-178619264							■	■													*PDE11A*
7:140946965-141125269								■													*SSBP1*
4:87361272-87632448								■													*MAPK10*
11:226871-233268							■	■													*PSMD13*
5:179857956-179876561							■	■													*CCR4*

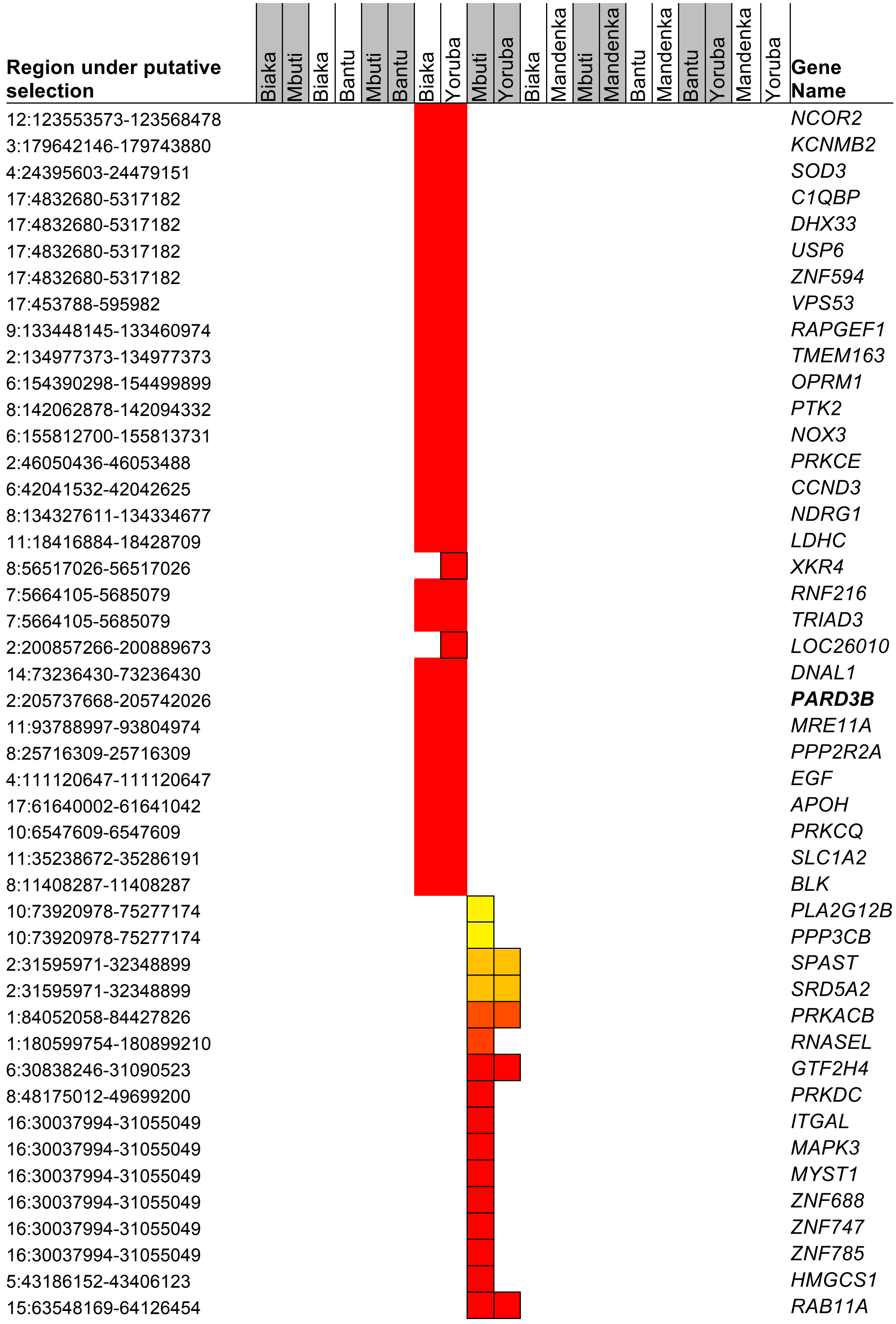
Region under putative selection
Biaka
Mbuti
Biaka
Bantu
Mbuti
Bantu
Biaka
Yoruba
Mbuti
Yoruba
Biaka
Mandenka
Mbuti
Mandenka
Bantu
Mandenka
Bantu
Yoruba
Mandenka
Yoruba
Gene Name
12:123553573-123568478 NCOR2
3:179642146-179743880 KCNMB2
4:24395603-24479151 SOD3
17:4832680-5317182 C1QBP
17:4832680-5317182 DHX33
17:4832680-5317182 USP6
17:4832680-5317182 ZNF594
17:453788-595982 VPS53
9:133448145-133460974 RAPGEF1
2:134977373-134977373 TMEM163
6:154390298-154499899 OPRM1
8:142062878-142094332 PTK2
6:155812700-155813731 NOX3
2:46050436-46053488 PRKCE
6:42041532-42042625 CCND3
8:134327611-134334677 NDRG1
11:18416884-18428709 LDHC
8:56517026-56517026 XKR4
7:5664105-5685079 RNF216
7:5664105-5685079 TRIAD3
2:200857266-200889673 LOC26010
14:73236430-73236430 DNAL1
2:205737668-205742026 PARD3B
11:93788997-93804974 MRE11A
8:25716309-25716309 PPP2R2A
4:111120647-111120647 EGF
17:61640002-61641042 APOH
10:6547609-6547609 PRKCQ
11:35238672-35286191 SLC1A2
8:11408287-11408287 BLK
10:73920978-75277174 PLA2G12B
10:73920978-75277174 PPP3CB
2:31595971-32348899 SPAST
2:31595971-32348899 SRD5A2
1:84052058-84427826 PRKACB
1:180599754-180899210 RNASEL
6:30838246-31090523 GTF2H4
8:48175012-49699200 PRKDC
16:30037994-31055049 ITGAL
16:30037994-31055049 MAPK3
16:30037994-31055049 MYST1
16:30037994-31055049 ZNF688
16:30037994-31055049 ZNF747
16:30037994-31055049 ZNF785
5:43186152-43406123 HMGCS1
15:63548169-64126454 RAB11A

Region under putative selection	Biaka	Mbuti	Biaka	Bantu	Mbuti	Bantu	Biaka	Yoruba	Mbuti	Yoruba	Biaka	Mandenka	Mbuti	Mandenka	Bantu	Mandenka	Bantu	Yoruba	Mandenka	Yoruba	Gene Name
15:63548169-64126454									■	■											*SLC24A1*
15:63548169-64126454									■	■											*RAB11A*
15:63548169-64126454									■	■											*SLC24A1*
6:33143855-33204252									■												*HLA-DPA1*
6:33143855-33204252									■												*HLA-DPB1*
12:111145487-111407744									■												*PTPN11*
2:94978884-96222641										■											*KCNIP3*
2:94978884-96222641										■											*LOC15076*
3:23902030-24094861									■												*UBE2E1*
13:49040596-49184219									■												*KPNA3*
12:55880161-56137449									■	■											*LRP1*
20:47080475-47422312									■												*STAU1*
6:90228257-90327535									■												*ANKRD6*
1:232618634-232739896									■												*TARBP1*
2:24725967-25165041									■												*ADCY3*
2:24725967-25165041									■												*NCOA1*
10:12875389-12882926									■												*CAMK1D*
2:171526992-171764496									■												*GORASP2*
3:170525673-170680718										■											*EVI1*
14:72511945-72861024										■											*RBM25*
5:74449469-74959712									■	■											*HMGCR*
5:74449469-74959712									■	■											*HMGCR*
11:9860202-10310293										■											*SBF2*
12:84078462-84240895									■	■											*ALX1*
12:84078462-84240895									■	■											*LRRIQ1*
16:74131925-74131925									■												*CHST5*
1:66059788-66283992										■											*PDE4B*
20:29698963-29872241									■	■											*BCL2L1*
20:29698963-29872241									■	■											*MYLK2*
9:2063839-2084590									■												*SMARCA2*
10:5984421-6042374									■	■											*FBXO18*
2:37372220-37372220									■	■											*PRKD3*
15:65187544-65200891									■												*SMAD3*
11:76932954-77168498									■	■											*CLNS1A*
10:95923791-95948321									■												*PLCE1*
19:10747206-10880543										■											*DNM2*
19:10747206-10880543										■											*TMED1*
4:74807802-74865179									■	■											*IL8*
6:154403712-154456266									■	■											*OPRM1*
17:3477153-3582394										■											*P2RX5*
1:116827132-116954482									■	■											*CD58*
17:40344589-40382401									■	■											*GFAP*
3:135213622-135213622									■												*SLCO2A1*
2:182045129-182346202										■											*ITGA4*
2:197199091-197423323										■											*GTF3C3*
10:12347969-12486578									■	■											*CAMK1D*

Region under putative selection	Biaka	Mbuti	Biaka	Bantu	Mbuti	Bantu	Biaka	Yoruba	Mbuti	Yoruba	Biaka	Mandenka	Mbuti	Mandenka	Bantu	Mandenka	Bantu	Yoruba	Mandenka	Yoruba	Gene Name
21:46597605-46610952																					*PCNT*
10:32896752-33276311																					*ITGB1*
7:81187348-81209555																					*HGF*
19:16439881-16462154																					*MED26*
3:133615389-133864830																					*CCBP2*
3:133615389-133864830																					*NPHP3*
19:50850483-50894012																					*SNRPD1*
19:50850483-50894012																					*SNRPD2*
4:110975660-111093375																					*EGF*
9:94481854-94638021																					*BICD2*
21:42390338-42390509																					*UMODL1*
7:98577167-98626525																					*SMURF1*
12:116179703-116179703																					*NOS1*
1:245111315-245125328																					*ELYS*
8:56518052-56518052																					*XKR4*
17:23086489-23226350																					*NOS2*
8:141775502-142083719																					*PTK2*
11:226871-232112																					*PSMD13*
11:226871-232112																					*PSMD13*
5:43206411-44084648																					*HMGCS1*
11:72149984-72565260																					*ATG16L2*
6:32873108-32920894																					*HLA-DOB*
6:32873108-32920894																					*PSMB8*
22:34061175-34252550																					*TOM1*
14:67125637-67884400																					*PIGH*
1:181275819-181527282																					*LAMC1*
1:181275819-181527282																					*LAMC2*
16:31318686-45409739																					*SHCBP1*
16:31318686-45409739																					*ZNF720*
5:118303768-118636074																					*DMXL1*
7:31799992-31863766																					*PDE1C*
11:5819108-5862649																					***TRIM5***
2:95252161-96621255																					*ARID5A*
2:95252161-96621255																					*KCNIP3*
2:95252161-96621255																					*LOC15076*
4:123237584-123721113																					*IL2*
2:162428347-162907534																					*DPP4*
10:6529779-6577462																					*PRKCQ*
19:46903147-47797213																					*ARHGEF1*
19:46903147-47797213																					*LYPD4*
19:46903147-47797213																					*RABAC1*
5:59264373-59418081																					*PDE4D*
1:153675259-154009934																					*DAP3*
7:140941527-141251553																					*SSBP1*
13:47482941-47979890																					*MED4*
13:47482941-47979890																					*RB1*

Region under putative selection	Biaka	Mbuti	Biaka	Bantu	Mbuti	Bantu	Biaka	Yoruba	Mbuti	Yoruba	Biaka	Mandenka	Mbuti	Mandenka	Bantu	Mandenka	Bantu	Yoruba	Mandenka	Yoruba	Gene Name
7:5664105-5687974																					*RNF216*
7:5664105-5687974																					*TRIAD3*
15:56693437-56790047																					*ADAM10*
17:5336107-5390389																					*NLRP1*
1:117694341-117831407																					*MAN1A2*
5:74656133-74984799																					*HMGCR*
11:108044814-108432036																					*DDX10*
14:30080368-30167096																					*SCFD1*
5:179200555-179254263																					*LOC51149*
22:30671957-30679063																					*YWHAH*
10:74564381-75086795																					*PPP3CB*
3:27420975-27565426																					*SLC4A7*
16:85993964-86143037																					*MAP1LC3*
9:133438117-133482300																					*RAPGEF1*
10:12475385-12486578																					*CAMK1D*
10:17785130-17931828																					*MRC1*
4:74825239-74977303																					*CXCL1*
4:74825239-74977303																					*IL8*
3:42830769-42841126																					*CCBP2*
8:56520828-56529289																					*XKR4*
10:35399625-35566580																					*CREM*
2:178476929-178541360																					*PDE11A*
5:131980304-132071250																					*IL13*
5:131980304-132071250																					*IL4*
5:131980304-132071250																					*KIF3A*
3:179769634-179775047																					*KCNMB2*
4:24396002-24511681																					*SOD3*
8:22853665-23075599																					*TNFRSF1*
8:22853665-23075599																					*TNFRSF1*
8:22853665-23075599																					*TNFRSF1*
5:167913103-168026100																					*PANK3*
11:18092622-18607834																					*GTF2H1*
11:18092622-18607834																					*LDHA*
11:18092622-18607834																					*LDHC*
11:18092622-18607834																					***TSG101***
11:128366132-128772691																					*RICS*
7:24826487-24835388																					*OSBPL3*
10:33247503-33289139																					*ITGB1*
15:82488383-83142863																					*GOLGA9P*
17:63980785-64144160																					*PRKAR1A*
11:35772348-35827583																					*TRIM44*
8:142072236-142082043																					*PTK2*
18:72273927-72311963																					*ZNF516*
8:134322120-134332892																					*NDRG1*
4:111105192-111173358																					*EGF*
6:154365602-154499899																					*OPRM1*

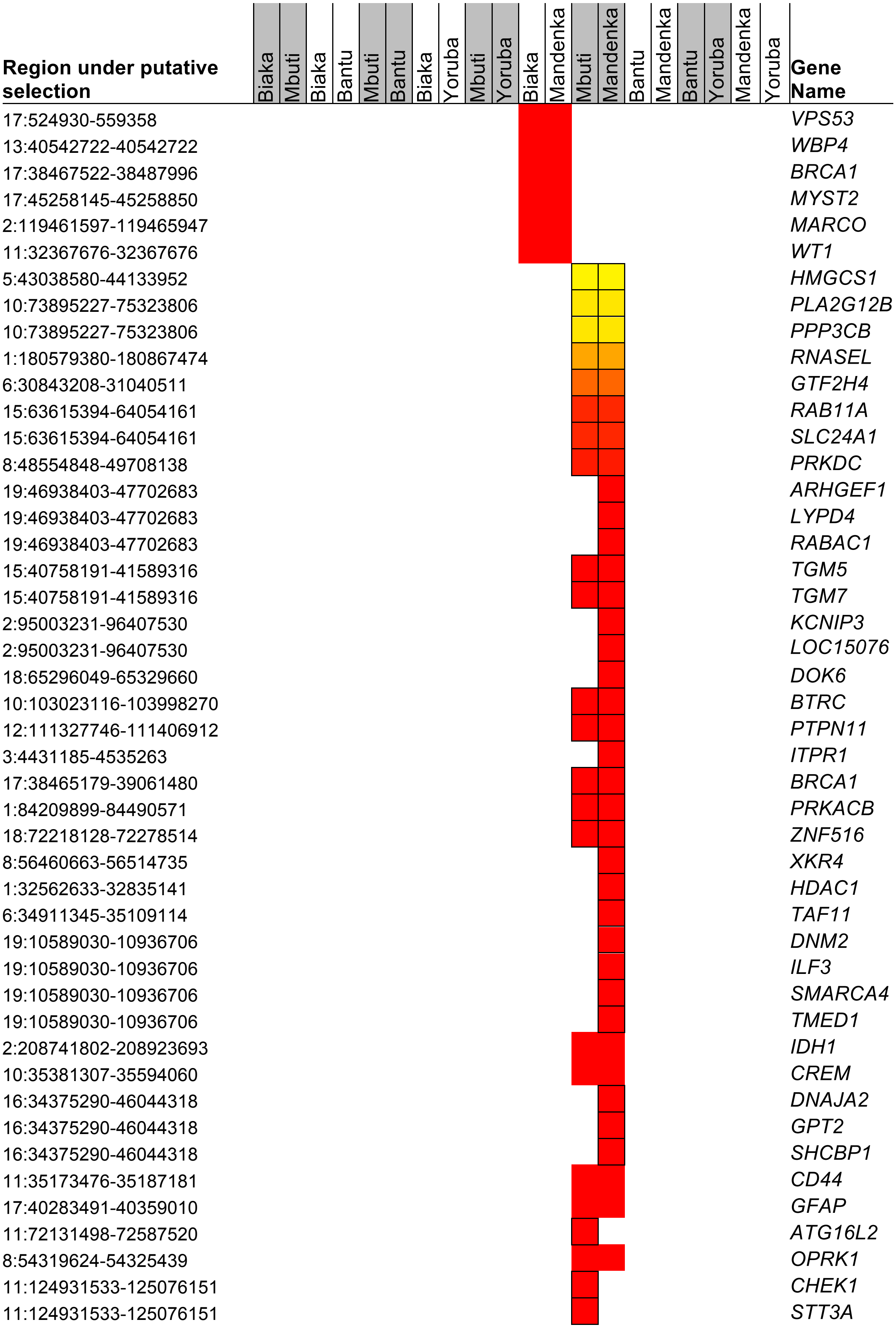
Region under putative selection
Biaka
Mbuti
Biaka
Bantu
Mbuti
Bantu
Biaka
Yoruba
Mbuti
Yoruba
Biaka
Mandenka
Mbuti
Mandenka
Bantu
Mandenka
Bantu
Yoruba
Mandenka
Yoruba
Gene Name
17:524930-559358 VPS53
13:40542722-40542722 WBP4
17:38467522-38487996 BRCA1
17:45258145-45258850 MYST2
2:119461597-119465947 MARCO
11:32367676-32367676 WT1
5:43038580-44133952 HMGCS1
10:73895227-75323806 PLA2G12B
10:73895227-75323806 PPP3CB
1:180579380-180867474 RNASEL
6:30843208-31040511 GTF2H4
15:63615394-64054161 RAB11A
15:63615394-64054161 SLC24A1
8:48554848-49708138 PRKDC
19:46938403-47702683 ARHGEF1
19:46938403-47702683 LYPD4
19:46938403-47702683 RABAC1
15:40758191-41589316 TGM5
15:40758191-41589316 TGM7
2:95003231-96407530 KCNIP3
2:95003231-96407530 LOC15076
18:65296049-65329660 DOK6
10:103023116-103998270 BTRC
12:111327746-111406912 PTPN11
3:4431185-4535263 ITPR1
17:38465179-39061480 BRCA1
1:84209899-84490571 PRKACB
18:72218128-72278514 ZNF516
8:56460663-56514735 XKR4
1:32562633-32835141 HDAC1
6:34911345-35109114 TAF11
19:10589030-10936706 DNM2
19:10589030-10936706 ILF3
19:10589030-10936706 SMARCA4
19:10589030-10936706 TMED1
2:208741802-208923693 IDH1
10:35381307-35594060 CREM
16:34375290-46044318 DNAJA2
16:34375290-46044318 GPT2
16:34375290-46044318 SHCBP1
11:35173476-35187181 CD44
17:40283491-40359010 GFAP
11:72131498-72587520 ATG16L2
8:54319624-54325439 OPRK1
11:124931533-125076151 CHEK1
11:124931533-125076151 STT3A

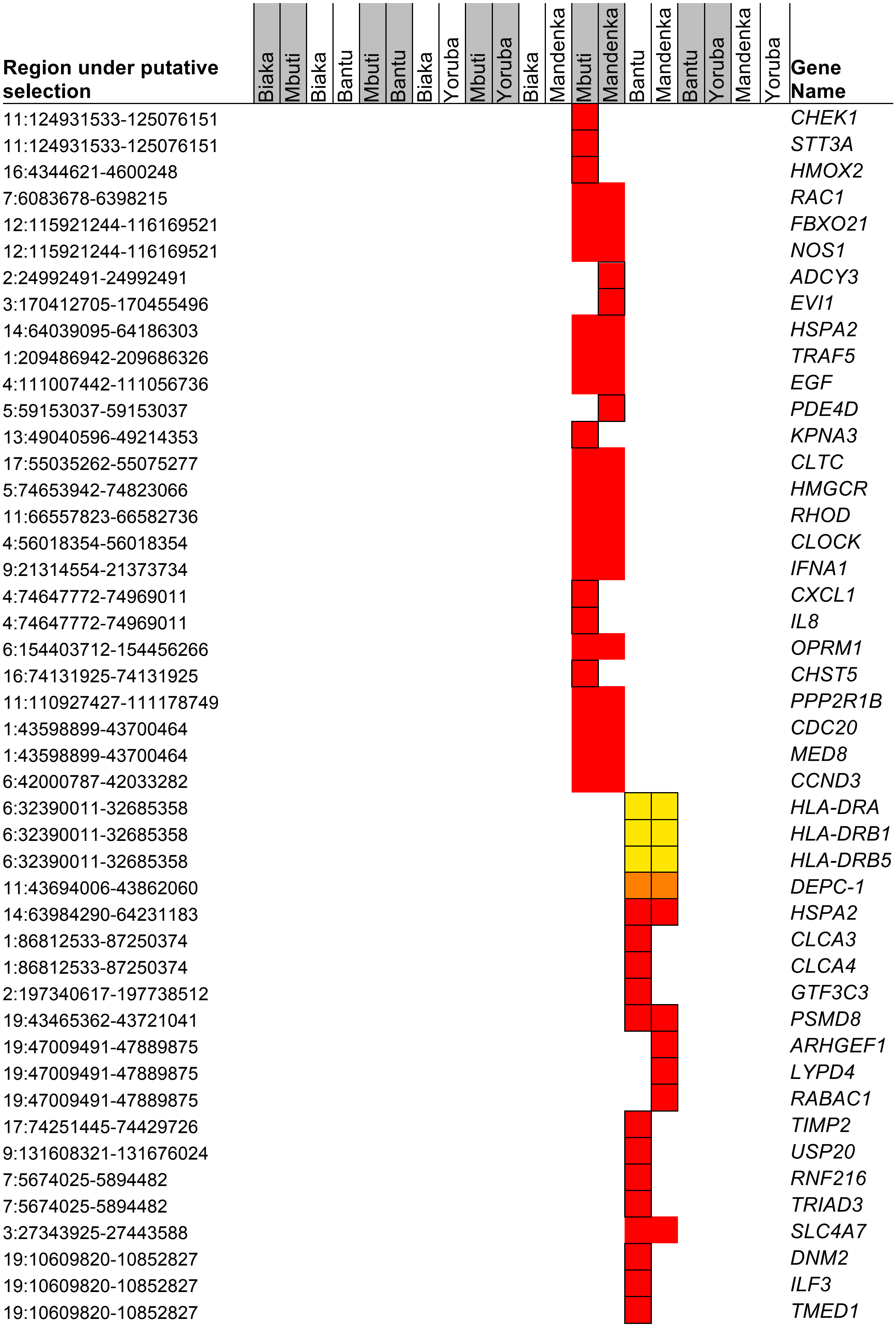
Region under putative selection
Biaka
Mbuti
Biaka
Bantu
Mbuti
Bantu
Biaka
Yoruba
Mbuti
Yoruba
Biaka
Mandenka
Mbuti
Mandenka
Bantu
Mandenka
Bantu
Yoruba
Mandenka
Yoruba
Gene Name
11:124931533-125076151 CHEK1
11:124931533-125076151 STT3A
16:4344621-4600248 HMOX2
7:6083678-6398215 RAC1
12:115921244-116169521 FBXO21
12:115921244-116169521 NOS1
2:24992491-24992491 ADCY3
3:170412705-170455496 EVI1
14:64039095-64186303 HSPA2
1:209486942-209686326 TRAF5
4:111007442-111056736 EGF
5:59153037-59153037 PDE4D
13:49040596-49214353 KPNA3
17:55035262-55075277 CLTC
5:74653942-74823066 HMGCR
11:66557823-66582736 RHOD
4:56018354-56018354 CLOCK
9:21314554-21373734 IFNA1
4:74647772-74969011 CXCL1
4:74647772-74969011 IL8
6:154403712-154456266 OPRM1
16:74131925-74131925 CHST5
11:110927427-111178749 PPP2R1B
1:43598899-43700464 CDC20
1:43598899-43700464 MED8
6:42000787-42033282 CCND3
6:32390011-32685358 HLA-DRA
6:32390011-32685358 HLA-DRB1
6:32390011-32685358 HLA-DRB5
11:43694006-43862060 DEPC-1
14:63984290-64231183 HSPA2
1:86812533-87250374 CLCA3
1:86812533-87250374 CLCA4
2:197340617-197738512 GTF3C3
19:43465362-43721041 PSMD8
19:47009491-47889875 ARHGEF1
19:47009491-47889875 LYPD4
19:47009491-47889875 RABAC1
17:74251445-74429726 TIMP2
9:131608321-131676024 USP20
7:5674025-5894482 RNF216
7:5674025-5894482 TRIAD3
3:27343925-27443588 SLC4A7
19:10609820-10852827 DNM2
19:10609820-10852827 ILF3
19:10609820-10852827 TMED1

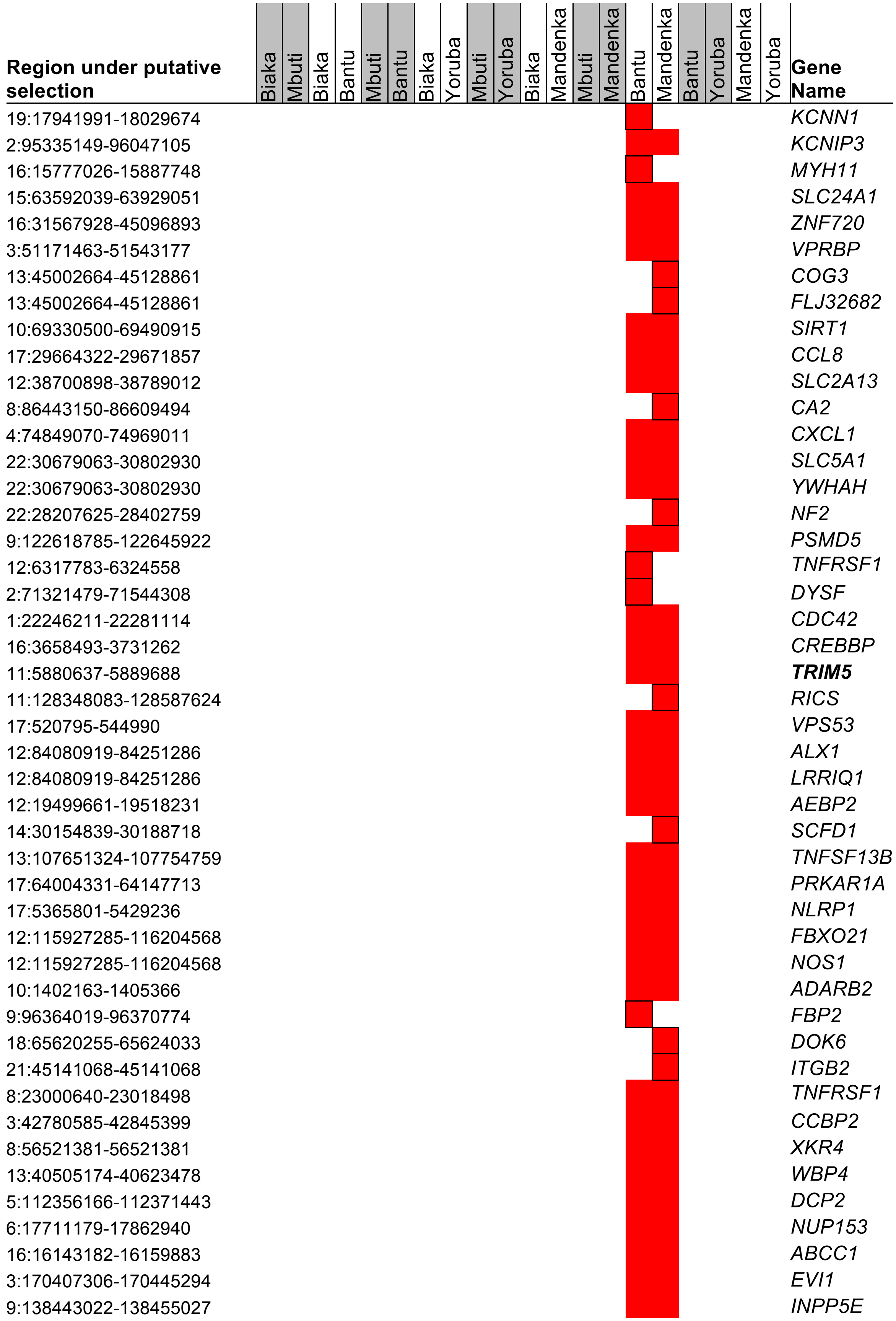
Region under putative selection
Biaka
Mbuti
Biaka
Bantu
Mbuti
Bantu
Biaka
Yoruba
Mbuti
Yoruba
Biaka
Mandenka
Mbuti
Mandenka
Bantu
Mandenka
Bantu
Yoruba
Mandenka
Yoruba
Gene Name
19:17941991-18029674
KCNN1
2:95335149-96047105
KCNIP3
16:15777026-15887748
MYH11
15:63592039-63929051
SLC24A1
16:31567928-45096893
ZNF720
3:51171463-51543177
VPRBP
13:45002664-45128861
COG3
13:45002664-45128861
FLJ32682
10:69330500-69490915
SIRT1
17:29664322-29671857
CCL8
12:38700898-38789012
SLC2A13
8:86443150-86609494
CA2
4:74849070-74969011
CXCL1
22:30679063-30802930
SLC5A1
22:30679063-30802930
YWHAH
22:28207625-28402759
NF2
9:122618785-122645922
PSMD5
12:6317783-6324558
TNFRSF1
2:71321479-71544308
DYSF
1:22246211-22281114
CDC42
16:3658493-3731262
CREBBP
11:5880637-5889688
TRIM5
11:128348083-128587624
RICS
17:520795-544990
VPS53
12:84080919-84251286
ALX1
12:84080919-84251286
LRRIQ1
12:19499661-19518231
AEBP2
14:30154839-30188718
SCFD1
13:107651324-107754759
TNFSF13B
17:64004331-64147713
PRKAR1A
17:5365801-5429236
NLRP1
12:115927285-116204568
FBXO21
12:115927285-116204568
NOS1
10:1402163-1405366
ADARB2
9:96364019-96370774
FBP2
18:65620255-65624033
DOK6
21:45141068-45141068
ITGB2
8:23000640-23018498
TNFRSF1
3:42780585-42845399
CCBP2
8:56521381-56521381
XKR4
13:40505174-40623478
WBP4
5:112356166-112371443
DCP2
6:17711179-17862940
NUP153
16:16143182-16159883
ABCC1
3:170407306-170445294
EVI1
9:138443022-138455027
INPP5E

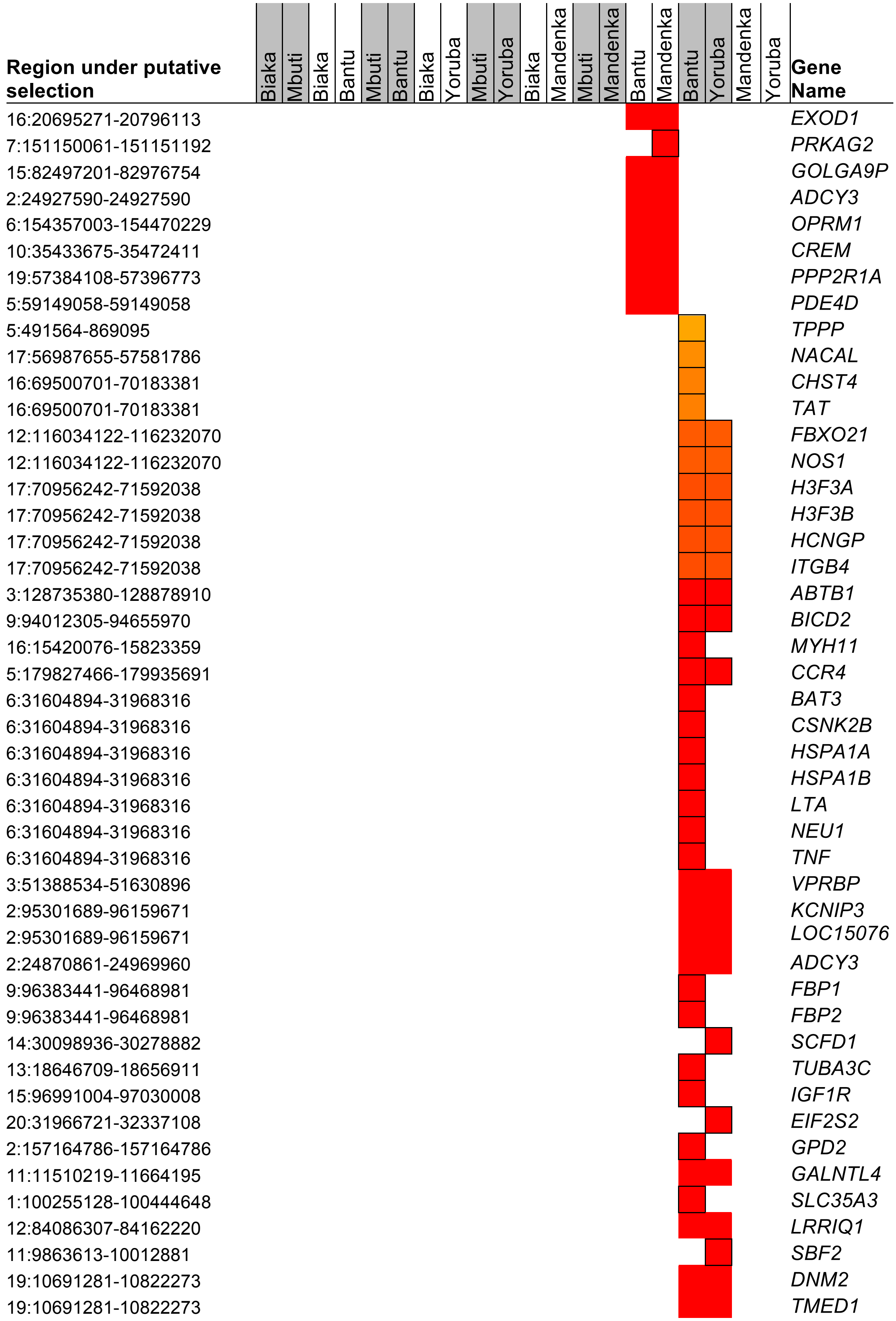
Region under putative selection
Biaka
Mbuti
Biaka
Bantu
Mbuti
Bantu
Biaka
Yoruba
Mbuti
Yoruba
Biaka
Mandenka
Mbuti
Mandenka
Bantu
Mandenka
Bantu
Yoruba
Mandenka
Yoruba
Gene Name
16:20695271-20796113 EXOD1
7:151150061-151151192 PRKAG2
15:82497201-82976754 GOLGA9P
2:24927590-24927590 ADCY3
6:154357003-154470229 OPRM1
10:35433675-35472411 CREM
19:57384108-57396773 PPP2R1A
5:59149058-59149058 PDE4D
5:491564-869095 TPPP
17:56987655-57581786 NACAL
16:69500701-70183381 CHST4
16:69500701-70183381 TAT
12:116034122-116232070 FBXO21
12:116034122-116232070 NOS1
17:70956242-71592038 H3F3A
17:70956242-71592038 H3F3B
17:70956242-71592038 HCNGP
17:70956242-71592038 ITGB4
3:128735380-128878910 ABTB1
9:94012305-94655970 BICD2
16:15420076-15823359 MYH11
5:179827466-179935691 CCR4
6:31604894-31968316 BAT3
6:31604894-31968316 CSNK2B
6:31604894-31968316 HSPA1A
6:31604894-31968316 HSPA1B
6:31604894-31968316 LTA
6:31604894-31968316 NEU1
6:31604894-31968316 TNF
3:51388534-51630896 VPRBP
2:95301689-96159671 KCNIP3
2:95301689-96159671 LOC15076
2:24870861-24969960 ADCY3
9:96383441-96468981 FBP1
9:96383441-96468981 FBP2
14:30098936-30278882 SCFD1
13:18646709-18656911 TUBA3C
15:96991004-97030008 IGF1R
20:31966721-32337108 EIF2S2
2:157164786-157164786 GPD2
11:11510219-11664195 GALNTL4
1:100255128-100444648 SLC35A3
12:84086307-84162220 LRRIQ1
11:9863613-10012881 SBF2
19:10691281-10822273 DNM2
19:10691281-10822273 TMED1

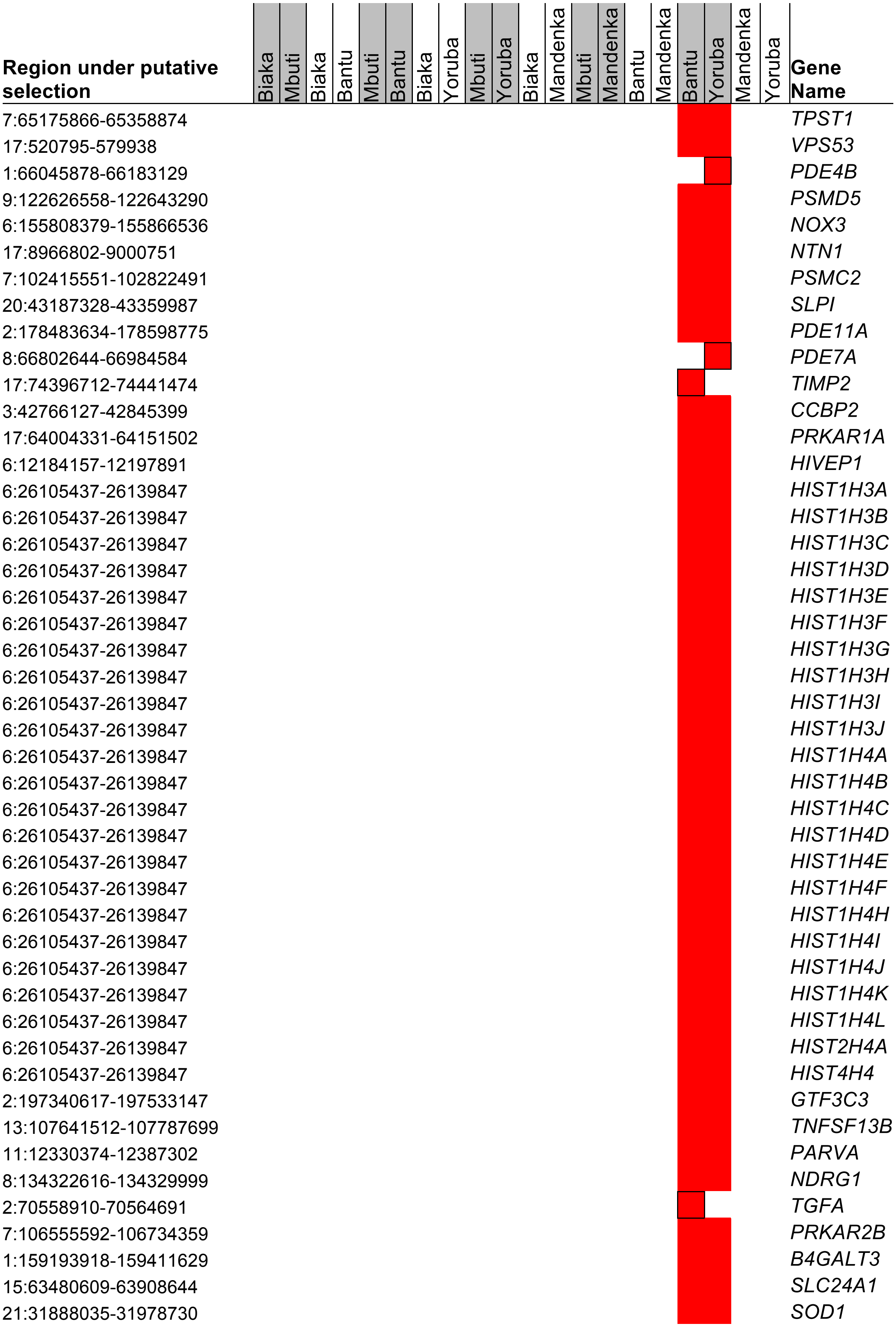
Region under putative selection
Biaka
Mbuti
Biaka
Bantu
Mbuti
Bantu
Biaka
Yoruba
Mbuti
Yoruba
Biaka
Mandenka
Mbuti
Mandenka
Bantu
Mandenka
Bantu
Yoruba
Mandenka
Yoruba
Gene Name
7:65175866-65358874 TPST1
17:520795-579938 VPS53
1:66045878-66183129 PDE4B
9:122626558-122643290 PSMD5
6:155808379-155866536 NOX3
17:8966802-9000751 NTN1
7:102415551-102822491 PSMC2
20:43187328-43359987 SLPI
2:178483634-178598775 PDE11A
8:66802644-66984584 PDE7A
17:74396712-74441474 TIMP2
3:42766127-42845399 CCBP2
17:64004331-64151502 PRKAR1A
6:12184157-12197891 HIVEP1
6:26105437-26139847 HIST1H3A
6:26105437-26139847 HIST1H3B
6:26105437-26139847 HIST1H3C
6:26105437-26139847 HIST1H3D
6:26105437-26139847 HIST1H3E
6:26105437-26139847 HIST1H3F
6:26105437-26139847 HIST1H3G
6:26105437-26139847 HIST1H3H
6:26105437-26139847 HIST1H3I
6:26105437-26139847 HIST1H3J
6:26105437-26139847 HIST1H4A
6:26105437-26139847 HIST1H4B
6:26105437-26139847 HIST1H4C
6:26105437-26139847 HIST1H4D
6:26105437-26139847 HIST1H4E
6:26105437-26139847 HIST1H4F
6:26105437-26139847 HIST1H4H
6:26105437-26139847 HIST1H4I
6:26105437-26139847 HIST1H4J
6:26105437-26139847 HIST1H4K
6:26105437-26139847 HIST1H4L
6:26105437-26139847 HIST2H4A
6:26105437-26139847 HIST4H4
2:197340617-197533147 GTF3C3
13:107641512-107787699 TNFSF13B
11:12330374-12387302 PARVA
8:134322616-134329999 NDRG1
2:70558910-70564691 TGFA
7:106555592-106734359 PRKAR2B
1:159193918-159411629 B4GALT3
15:63480609-63908644 SLC24A1
21:31888035-31978730 SOD1

Region under putative selection	Biaka	Mbuti	Biaka	Bantu	Mbuti	Bantu	Biaka	Yoruba	Mbuti	Yoruba	Biaka	Mandenka	Mbuti	Mandenka	Bantu	Mandenka	Bantu	Yoruba	Mandenka	Yoruba	Gene Name
4:87545748-87724510																					*MAPK10*
20:52109454-52115612																					*BCAS1*
8:56520828-56520828																					*XKR4*
10:88291311-88607372																					*OPN4*
13:40525677-40621779																					*WBP4*
11:214393-226871																					*PSMD13*
11:214393-226871																					*PSMD13*
6:154402201-154429999																					*OPRM1*
1:40520506-40520506																					*ZMPSTE2*
2:68244591-68366991																					*PPP3R1*
10:1718840-1729576																					*ADARB2*
1:166963238-167011760																					*DPT*
2:230748877-230767198																					*SP110*
6:152328988-152334365																					*ESR1*
17:31064899-31078012																					*AP2B1*
4:74866956-74956794																					*CXCL1*
14:23800883-23863602																					*ADCY4*
14:23800883-23863602																					*TGM1*
11:66084671-66272625																					*SPTBN2*
1:182269497-182290152																					*GLT25D2*
7:37330503-37331191																					*ELMO1*
6:167412542-167412542																					*CCR6*
6:32487714-32766602																					*HLA-DQA1*
6:32487714-32766602																					*HLA-DQB1*
6:32487714-32766602																					*HLA-DRA*
6:32487714-32766602																					*HLA-DRB1*
6:32487714-32766602																					*HLA-DRB5*
17:44183411-44439710																					*UBE2Z*
11:128312133-128619142																					*RICS*
3:170577457-170694461																					*EVI1*
5:43272945-44113129																					*HMGCS1*
5:59807480-59975475																					*PDE4D*
13:107712281-107754318																					*TNFSF13B*
11:63718728-63805488																					*BAD*
11:63718728-63805488																					*PLCB3*
11:63718728-63805488																					*STIP1*
11:63718728-63805488																					*TRPT1*
12:85235742-85966611																					*MGAT4C*
21:45136241-45141068																					*ITGB2*
11:3656040-3713262																					*NUP98*
2:95092780-96173058																					*KCNIP3*
2:95092780-96173058																					*LOC15076*
2:197969715-198351666																					*HSPD1*
2:197969715-198351666																					*HSPE1*
2:197969715-198351666																					*SF3B1*
4:22315249-22320167																					*GBA3*

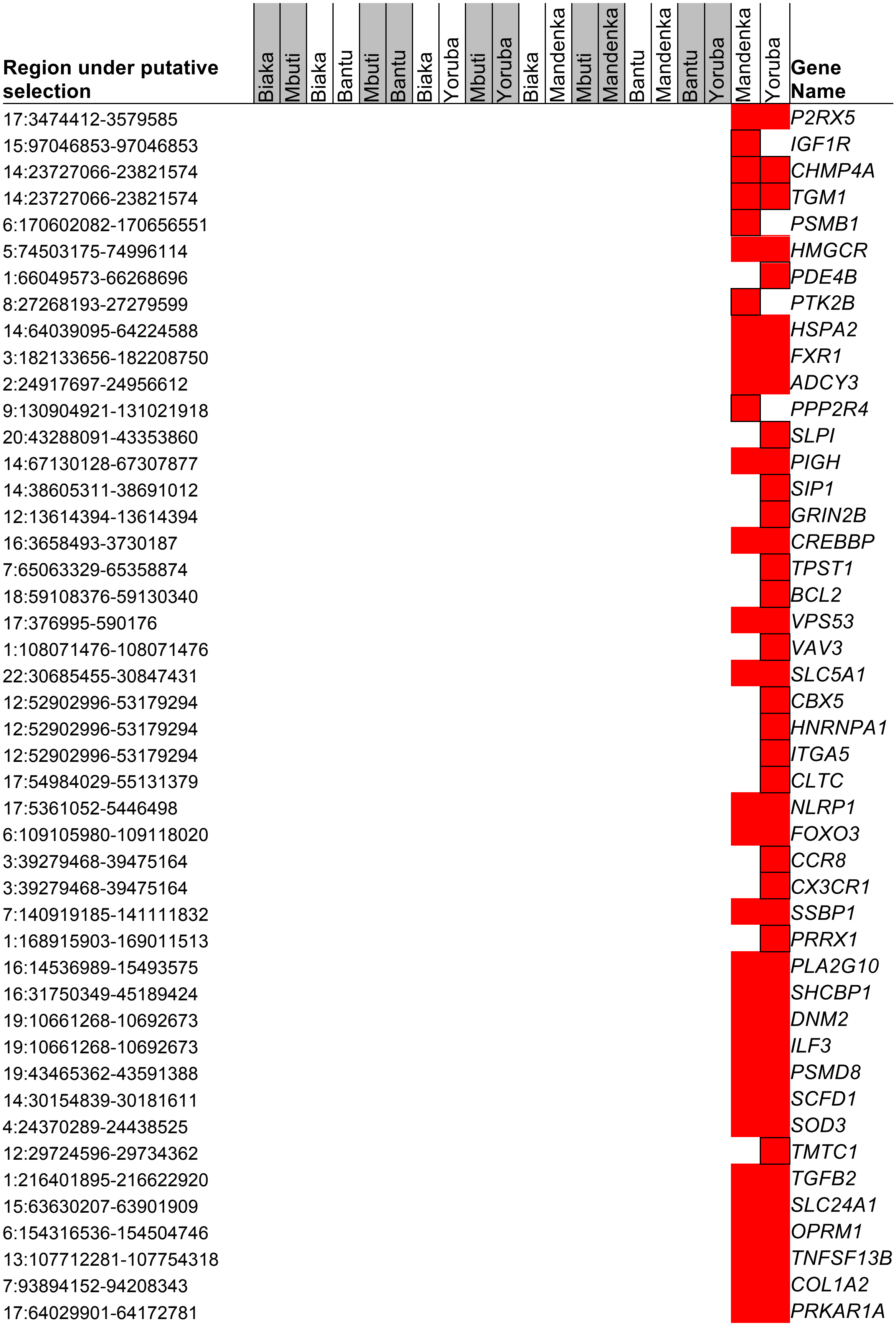
Region under putative selection
Biaka
Mbuti
Biaka
Bantu
Mbuti
Bantu
Biaka
Yoruba
Mbuti
Yoruba
Biaka
Mandenka
Mbuti
Mandenka
Bantu
Mandenka
Bantu
Yoruba
Mandenka
Yoruba
Gene Name
17:3474412-3579585
P2RX5
15:97046853-97046853
IGF1R
14:23727066-23821574
CHMP4A
14:23727066-23821574
TGM1
6:170602082-170656551
PSMB1
5:74503175-74996114
HMGCR
1:66049573-66268696
PDE4B
8:27268193-27279599
PTK2B
14:64039095-64224588
HSPA2
3:182133656-182208750
FXR1
2:24917697-24956612
ADCY3
9:130904921-131021918
PPP2R4
20:43288091-43353860
SLPI
14:67130128-67307877
PIGH
14:38605311-38691012
SIP1
12:13614394-13614394
GRIN2B
16:3658493-3730187
CREBBP
7:65063329-65358874
TPST1
18:59108376-59130340
BCL2
17:376995-590176
VPS53
1:108071476-108071476
VAV3
22:30685455-30847431
SLC5A1
12:52902996-53179294
CBX5
12:52902996-53179294
HNRNPA1
12:52902996-53179294
ITGA5
17:54984029-55131379
CLTC
17:5361052-5446498
NLRP1
6:109105980-109118020
FOXO3
3:39279468-39475164
CCR8
3:39279468-39475164
CX3CR1
7:140919185-141111832
SSBP1
1:168915903-169011513
PRRX1
16:14536989-15493575
PLA2G10
16:31750349-45189424
SHCBP1
19:10661268-10692673
DNM2
19:10661268-10692673
ILF3
19:43465362-43591388
PSMD8
14:30154839-30181611
SCFD1
4:24370289-24438525
SOD3
12:29724596-29734362
TMTC1
1:216401895-216622920
TGFB2
15:63630207-63901909
SLC24A1
6:154316536-154504746
OPRM1
13:107712281-107754318
TNFSF13B
7:93894152-94208343
COL1A2
17:64029901-64172781
PRKAR1A

Region under putative selection	Biaka	Mbuti	Biaka	Bantu	Mbuti	Bantu	Biaka	Yoruba	Mbuti	Yoruba	Biaka	Mandenka	Mbuti	Mandenka	Bantu	Mandenka	Bantu	Yoruba	Mandenka	Yoruba	Gene Name
7:6702059-6709622																			■	■	*ZNF12*
5:7850291-7885119																			■	■	*ADCY2*
12:116028796-116173554																			■	■	*FBXO21*
12:116028796-116173554																			■	■	*NOS1*
11:110974172-111139465																			■	■	*PPP2R1B*
20:57019769-57031040																				■	*TUBB1*
1:10997169-11216546																			■	■	*EXOSC10*
1:10997169-11216546																			■	■	*FRAP1*
1:10997169-11216546																			■	■	*TARDBP*
2:119447658-119459054																			■	■	*MARCO*
9:133447401-133460974																			■	■	*RAPGEF1*
18:72239551-72323185																			■	■	*ZNF516*
10:6175031-6201787																				■	*RBM17*
4:111015411-111093375																			■	■	*EGF*
8:142030361-142093418																			■	■	*PTK2*
2:182107731-182166048																				■	*ITGA4*
9:122537416-122650109																			■	■	*PSMD5*
9:119463408-119557665																			■	■	*TLR4*
3:197235139-197311602																			■	■	*TFRC*
1:22281114-22281114																			■	■	*CDC42*
1:159311752-159447136																			■	■	*B4GALT3*
5:179870080-179884180																			■	■	*CCR4*
8:56517026-56520828																			■	■	*XKR4*
13:40525677-40616108																			■	■	*WBP4*
12:15652110-15671192																				■	*EPS8*
6:152328988-152334353																				■	*ESR1*
2:178496734-178541360																			■	■	*PDE11A*
8:134331714-134332521																			■	■	*NDRG1*
6:41881300-42042625																			■	■	*CCND3*
6:41881300-42042625																			■	■	*MED20*
3:170577457-170694461																			■	■	*EVI1*
3:170577457-170694461																			■	■	*EVI1*
5:179201385-179206426																			■	■	*LOC51149*
22:38044162-38044162																			■	■	*RPL3*
